# Chiral hemicucurbit[8]uril as an anion receptor: selectivity to size, shape and charge distribution†
†Electronic supplementary information (ESI) available: MS, NMR, dynamic NMR and computational details and a DFT-based video of complexation. CCDC 1514736–1514741, 1521388. For ESI and crystallographic data in CIF or other electronic format see DOI: 10.1039/c6sc05058a



**DOI:** 10.1039/c6sc05058a

**Published:** 2016-11-30

**Authors:** Sandra Kaabel, Jasper Adamson, Filip Topić, Anniina Kiesilä, Elina Kalenius, Mario Öeren, Mart Reimund, Elena Prigorchenko, Aivar Lõokene, Hans J. Reich, Kari Rissanen, Riina Aav

**Affiliations:** a Department of Chemistry , Tallinn University of Technology , Akadeemia tee 15 , 12618 Tallinn , Estonia . Email: riina.aav@ttu.ee; b University of Jyvaskyla , Department of Chemistry , Nanoscience Center , P.O. Box. 35 , FI-40014 Jyvaskyla , Finland . Email: kari.t.rissanen@jyu.fi; c National Institute of Chemical Physics and Biophysics , Akadeemia tee 23 , 12618 Tallinn , Estonia; d Department of Chemistry , University of Wisconsin , Madison , WI 53706 , USA

## Abstract

A novel eight-membered macrocycle of the hemicucurbit[*n*]uril family, chiral (*all-R*)-cyclohexanohemicucurbit[8]uril (**cycHC[8]**)‡
‡The name cyclohexylhemicucurbituril, previously used for these macrocycles, is changed in accordance with the IUPAC nomenclature for fused cycles, as the cyclohexane substituents are fused with the parent hemicucurbituril. binds anions in a purely protic solvent with remarkable selectivity. The **cycHC[8]** portals open and close to fully encapsulate anions in a 1 : 1 ratio, resembling a molecular Pac-Man™. Comprehensive gas, solution and solid phase studies prove that the binding is governed by the size, shape and charge distribution of the bound anion. Gas phase studies show an order of SbF_6_^–^ ≈ PF_6_^–^ > ReO_4_^–^ > ClO_4_^–^ > SCN^–^ > BF_4_^–^ > HSO_4_^–^ > CF_3_SO_3_^–^ for anion complexation strength. An extensive crystallographic study reveals the preferred orientations of the anions within the octahedral cavity of **cycHC[8]** and highlights the importance of the size- and shape-matching between the anion and the receptor cavity. The solution studies show the strongest binding of the ideally fitting SbF_6_^–^ anion, with an association constant of 2.5 × 10^5^ M^–1^ in pure methanol. The symmetric, receptor cavity-matching charge distribution of the anions results in drastically stronger binding than in the case of anions with asymmetric charge distribution. Isothermal titration calorimetry (ITC) reveals the complexation to be exothermic and enthalpy-driven. The DFT calculations and VT-NMR studies confirmed that the complexation proceeds through a pre-complex formation while the exchange of methanol solvent with the anion is the rate-limiting step. The octameric **cycHC[8]** offers a unique example of template-controlled design of an electroneutral host for binding large anions in a competitive polar solvent.

‡The name cyclohexylhemicucurbituril, previously used for these macrocycles, is changed in accordance with the IUPAC nomenclature for fused cycles, as the cyclohexane substituents are fused with the parent hemicucurbituril.

## Abstract 2

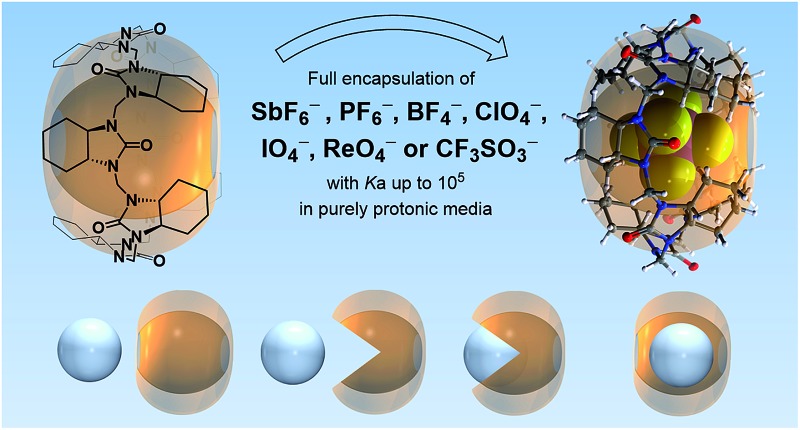
Chiral (*all-R*)-cyclohexanohemicucurbit[8]uril binds anions in a 1 : 1 ratio in pure methanol like a molecular Pac-Man™ with remarkable selectivity based on the size, shape and charge distribution of the anion.

## Introduction

The importance of ion recognition and transport in biological systems is well established, bringing about the quest for synthetic receptors capable of binding ions in physiological conditions. The syntheses of crown ethers,^1^ cryptands^2^ and cucurbiturils^3^ among others have contributed to the rich history of cation recognition whereas development of anion receptors effective in protic solvents remains challenging.^4–7^ Anion sensing motifs based on cationic species are usually effective in a narrow range of pH, plagued by low binding selectivity and counteranion competition for the binding site. Neutral anion receptors relying on hydrogen or halogen bonding are often affected by strong competition from the protic solvent with host–guest interactions having to disrupt the solvation shell of the anion.^8,9^

Exemplary studies on anion binding by cyclic hexapeptides show that introducing a confined cavity to the structure of the receptor significantly increases the anion binding ability, as abundant 2 : 1 host–guest complexes were observed with halides and SO_4_^2–^ where the anion was enclosed in the cavity formed by two cyclopeptide units.^10,11^ This led to the design of sandwich-like bis(cyclopeptide) receptors that reached association constants of up to 10^6^ M^–1^ for binding SO_4_^2–^ in a water–methanol mixture.^12,13^ Employing a hydrophobic pocket for anion binding in water has been illustrated by the Gibb's octa-acid cavitand,^14^ where the binding of partially hydrated anions yielded association constants up to 10^3^ M^–1^ at pH 11.5. Likewise, the size-dependent complexation of dodecaborate dianions within the hydrophobic cavity of γ-cyclodextrin was studied by Nau and co-workers, with the strongest association in the case of B_12_Br_12_^2–^ (*K*_a_ = 9.6 × 10^5^ M^–1^).^15^

Cucurbituril family members have been explored as ion receptors due to their well-defined hydrophobic cavity.^16,17^ Hemicucurbiturils,^18^ cyclohexanohemicucurbit[6]urils,^19^ bambus[6]urils^20^ and biotin[6]urils^21–23^ have been shown to bind anions within their electron-deficient (*i.e.* partially positively charged) hydrophobic cavities. Presently, bambus[6]urils hold the record for the strongest anion binding (*K*_a_ = 5.5 × 10^7^ M^–1^) by a neutral host in exclusively protic solvent.^24^ Based on the crystal structures of six-membered hemicucurbiturils, bambus[6]uril^25–28^ and biotin[6]uril^21^ complexes the central cavities readily accommodate halide anions. Larger anions have been shown to prefer the formation of 1 : 2 host : guest complexes with double-cone-shaped bambus[6]urils,^29,30^ with the anions bound away from the centre of the cavity.

We have previously demonstrated the synthesis of the first 8-membered hemicucurbituril, (*all-R*)-cyclohexanohemicucurbit[8]uril (**cycHC[8]**), by an approach using anion-templating.^31^ Given that larger anions such as PF_6_^–^ and CF_3_CO_2_^–^ were observed to act as effective templates, we decided to investigate the binding of other larger inorganic anions by **cycHC[8]**. Such anions are for example used in ionic liquids^32^ (BF_4_^–^, PF_6_^–^, SbF_6_^–^, CF_3_SO_3_^–^) and as oxidizing agents^33^ (ClO_4_^–^, IO_4_^–^). On the other hand, they are considered as environmental pollutants.^33,34^ Binding of these anions in protic media is important from a biological and environmental point of view.

## Results and discussion

**Fig. 1:**
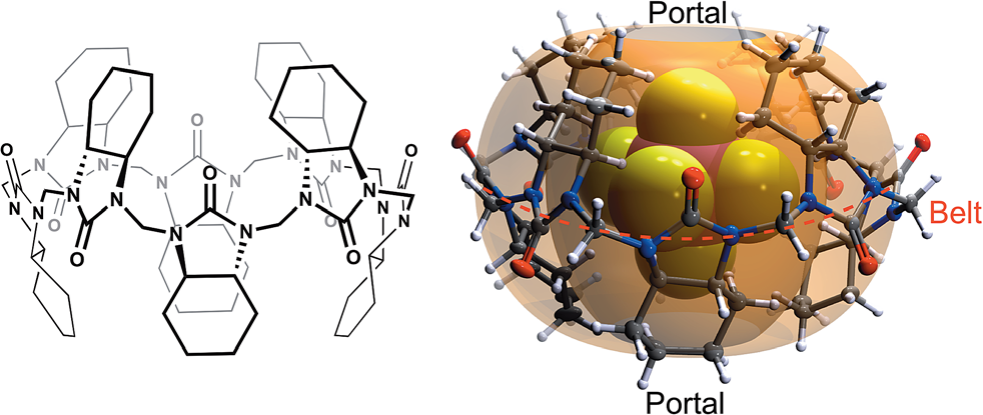
Molecular structure of (*all-R*)-cyclohexanohemicucurbit[8]uril, **cycHC[8]** (left), and the X-ray structure of an inclusion complex with SbF_6_^–^ (right).

The chiral host molecule (*all-R*)-cyclohexanohemicucurbit[8]uril^31,35^ (**cycHC[8]**) fully encapsulates certain anions forming 1 : 1 complexes (Fig. 1) with high selectivity and binding affinities of up to 2.5 × 10^5^ M^–1^ in methanol.

The scope of anionic guests that form complexes with **cycHC[8]** was determined by mass spectrometry (see ESI†). Only 1 : 1 complexes were observed in ESI-MS spectra and abundant complexes were observed with SbF_6_^–^ ≈ PF_6_^–^ > ReO_4_^–^ > ClO_4_^–^ > SCN^–^ > BF_4_^–^ > HSO_4_^–^ > CF_3_SO_3_^–^, ranked by decreasing affinity for **cycHC[8]**. The anions H_2_PO_4_^–^, AcO^–^, Br^–^, Cl^–^, I^–^, F^–^, NO_3_^–^ and NO_2_^–^ showed only weak complexation, while no complexes were formed with AuBr_4_^–^, Br_3_^–^ and CN^–^. The order of affinity was ascertained through competition experiments, performed on three-component mixtures of the host with two competing anions in 1 : 1 : 1 molar ratio (ESI Fig. S2 and S3†). Halide anions (13 to 35 Å^3^ in volume^36^), while readily forming complexes with 6-membered hemicucurbiturils,^37–41^ appear to have very low affinity towards **cycHC[8]**, presumably due to a mismatch in size with the cavity of **cycHC[8]**. As expected, the affinity towards more heavily solvated anions was found to be lower than for weakly solvated ones. On the other hand, tetrahedral and octahedral anions falling into the volume range of 50 to 80 Å^3^ form stronger complexes with **cycHC[8]**, presumably due to a better size fit. This is also in good agreement with Rebek's rule suggesting a packing coefficient (PC)^42^ of 0.55 ± 0.09 for optimal fit in the **cycHC[8]** cavity with a volume of 123 Å^3^.^31^ The MS/MS collision-induced dissociation (CID) experiments (Fig. S4†) on isolated complexes revealed the most efficient dissociation (lowest kinetic stability) for the host–guest complexes with highest PCs (>0.55). This results from the sensitive interplay of the attractive and repulsive forces between the anion and the cavity walls, and the lack of void space. More importantly, it also indicates the full encapsulation of the anions in the complexes in the gas phase. The same phenomenon has been studied in detail with cucurbiturils and azoalkanes,^43^ but is reported here for the first time with anionic complexes.

**Fig. 2:**
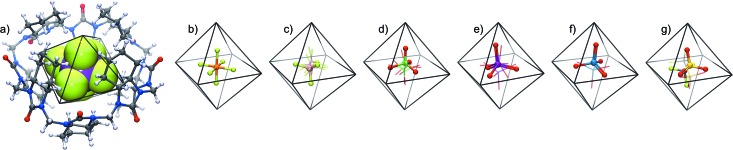
The crystal structures of 1 : 1 host–guest complexes of (a) SbF_6_^–^, (b) PF_6_^–^, (c) BF_4_^–^, (d) ClO_4_^–^, (e) IO_4_^–^, (f) ReO_4_^–^ and (g) CF_3_SO_3_^–^ anions in the **cycHC[8]** cavity. Minor disorder components are shown as a wireframe model. The host in (b–f) is represented by an octahedron depicting the cavity of **cycHC[8]**.

**Fig. 3:**
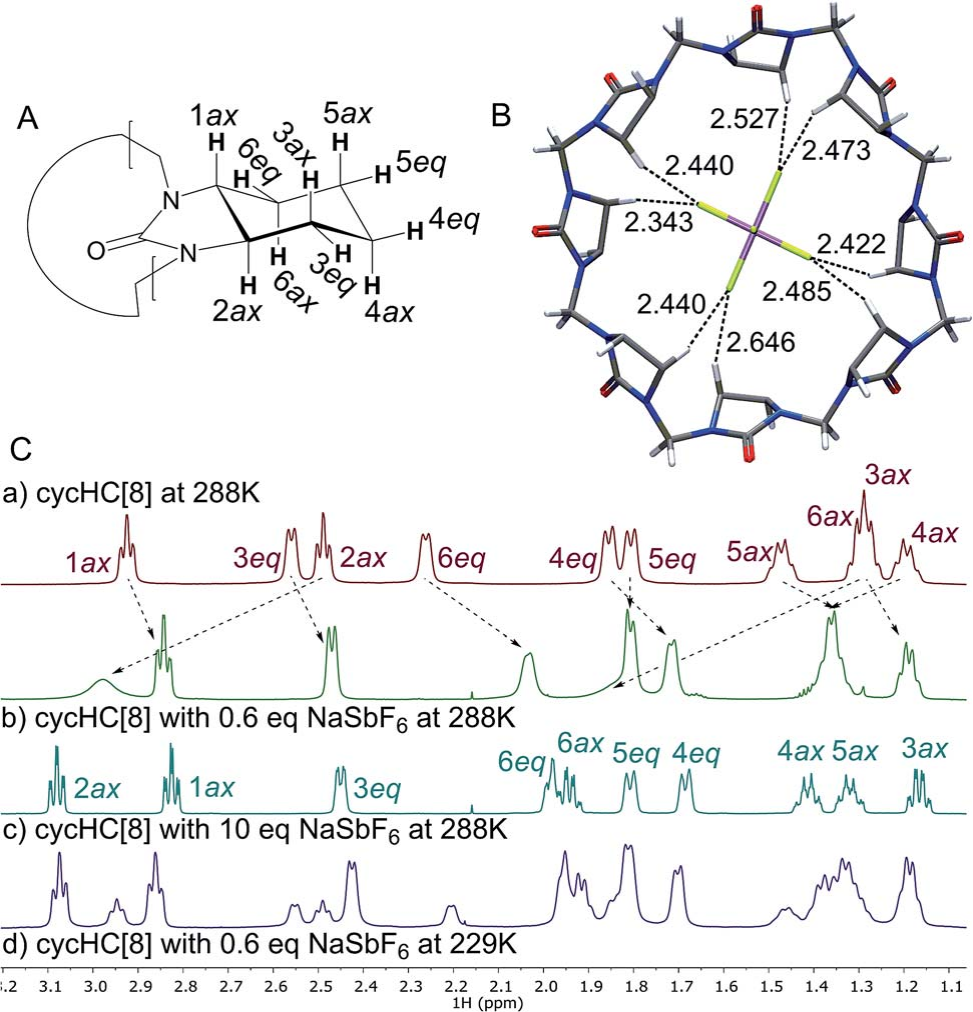
(A) Labelling of **cycHC[8]** protons, (B) C–H···F distances between the host proton 2*ax* and SbF_6_^–^ from the X-ray structure; all (CH_2_)_4_ groups are omitted for clarity, (C) ^1^H NMR in MeOD of (a) free **cycHC[8]**, (b) **cycHC[8]** with 0.6 eq. of NaSbF_6_ at 288 K (c) **cycHC[8]** with 10 eq. of NaSbF_6_ at 288 K; (d) SbF_6_^–^@**cycHC[8]** and free **cycHC[8]** formed from **cycHC[8]** with 0.6 eq. of NaSbF_6_ at 229 K.

The crystal structures of the host–guest complexes, obtained by single crystal X-ray diffraction, demonstrate unambiguously the 1 : 1 stoichiometry of the anion inclusion complexes in the solid state (Fig. 2). Single crystals of the complexes were obtained from solutions of **cycHC[8]** in methanol with the guest added as a tetrabutylammonium (TBA) or tetrabutyl-phosphonium (TBP) salt. The TBA or TBP cations and a number of solvent molecules fill the space between the capsule-like moieties, affording isostructural crystals regardless of the guest anion used. The guest anions are situated at the center of the **cycHC[8]** cavity, in a manner depending on their size and shape. The octahedral anions SbF_6_^–^ and PF_6_^–^ are locked in a fixed position showing no disorder. Four fluorine atoms of SbF_6_^–^/PF_6_^–^ lying on the equatorial plane of the macrocycle point to the four corners of its square-shaped belt, while the two remaining fluorines point to the opposite portals (Fig. 2a and b). The Hirshfeld surface^44^ plotted for the encapsulated octahedral anion SbF_6_^–^ indicates that the host–guest C–H···F interactions are responsible for the fixed orientation of these guests (ESI Fig. S6†). The shortest C–H···F distances are found between the four equatorial fluorine atoms and the axial 2*ax* protons in each corner of the macrocyclic cavity (Fig. 3B and Table S3†). In contrast to the octahedral anions, the tetrahedral anions BF_4_^–^, ClO_4_^–^, ReO_4_^–^ and IO_4_^–^ have more space inside the cavity (Fig. 2c–f) and show disorder in the crystal. As the host cavity is symmetric and therefore offers a number of equal interaction sites, several orientations of the tetrahedral anions are equally favored. The Hirshfeld surface of encapsulated IO_4_^–^ (Fig. S7†), together with the close contact analysis of other tetrahedral anions (Tables S5–S8†) reveals that these anions can only form a limited number of interactions with the host cavity wall in a given orientation. Smaller anions like BF_4_^–^ and ClO_4_^–^ can form even fewer host–guest interactions simultaneously. The large CF_3_SO_3_^–^ anion has two orientations (Fig. 2g and Table S9†). Given that **cycHC[8]** preserves its conformation almost fully regardless of the guest anion encapsulated, its cavity can be considered as an octahedrally shaped void, which encapsulates guests in a manner depending on their shape, volume and complementarity of the interactions.

Next, we examined anion complexation with **cycHC[8]** in solution using ^1^H NMR spectroscopy. The complex with SbF_6_^–^ showed the largest complexation-induced chemical shift changes (downfield shifts of 0.52, 0.23 and 0.64 ppm for 2*ax*, 4*ax* and 6*ax*, respectively). The signals for 2*ax*, 4*ax* and 6*ax* in the complex with SbF_6_^–^ also showed significant signal broadening at room temperature, indicating a slow guest exchange rate for SbF_6_^–^. The guest exchange slows down at low temperature resulting in the signals of SbF_6_^–^@**cycHC[8]** and excess free **cycHC[8]** being separate (Fig. 3C(d)).

The Job plot analysis confirmed 1 : 1 stoichiometry of binding for all studied guests in methanol (ESI†), as also observed by crystallography in the solid state and by mass spectrometry in the gas phase. Association constants were determined by NMR titrations (Table 1), simultaneously following three **cycHC[8]** proton signals 1*ax*, 2*ax* and 3*eq* (Fig. 3A). The association constant for SbF_6_^–^ was, due to the broadening of the 2*ax* signal, determined only from the 1*ax* and 3*eq* proton signals. The range of association constants varied over five orders of magnitude, strongly dependent on the size and shape of the guest. The poor water solubility of **cycHC[8]** prevented measurements in pure water, but no significant decrease in binding strength was observed when pure methanol was changed to a 1 : 1 methanol/water mixture (Table 1, rows 2–3).

**Table 1:** Association constants *K*_a_ for **cycHC[8]** inclusion complexes with anions, measured in MeOD at 288 K by ^1^H NMR titration experiments. Volumes of the anions (*V*_anion_) and their packing coefficients (PC)^*a*^

Anion	Cation	*V*_anion_ (Å^3^)	PC	*K*_a_ (M^–1^)
SbF_6_^–^	Na^+^	81.8	0.67	(2.5 ± 0.7) × 10^5^
PF_6_^–^	Bu_4_N^+^	70.6	0.57	(2.8 ± 0.4) × 10^4^
PF_6_^–^ ^*b*^	Bu_4_N^+^	70.6	0.57	(2.6 ± 0.2) × 10^4^
PF_6_^–^	Na^+^	70.6	0.57	(2.0 ± 0.2) × 10^4^
ReO_4_^–^	Bu_4_N^+^	64.8	0.53	(4.7 ± 0.4) × 10^3^
IO_4_^–^	Na^+^	64.3	0.52	(1.8 ± 0.2) × 10^3^
ClO_4_^–^	Bu_4_N^+^	54.7	0.45	(4.7 ± 0.2) × 10^2^
BF_4_^–^	Bu_4_N^+^	51.6	0.42	(4.8 ± 0.4) × 10
CF_3_SO_3_^–^	Bu_4_N^+^	82.3	0.67	(3.9 ± 0.5) × 10
CF_3_CO_2_^–^	Bu_4_N^+^	68.7	0.56	<10

^*a*^Anion volume is based on optimized anion geometries (BP86-D/def2-TZVPD) and calculated using a triangulated sphere model (based on CSD default atomic radii) through the Olex2 program package.^45^ PC is defined as the ratio between the *V*_anion_ to *V*_cavity_(host).^42^ *V*_cavity_(**cycHC[8]**) = 123.0 Å^3^ is measured from the crystal structure of **cycHC[8]**.^31^

^*b*^ ^1^H NMR in 1 : 1 MeOD/D_2_O.

**Fig. 4:**
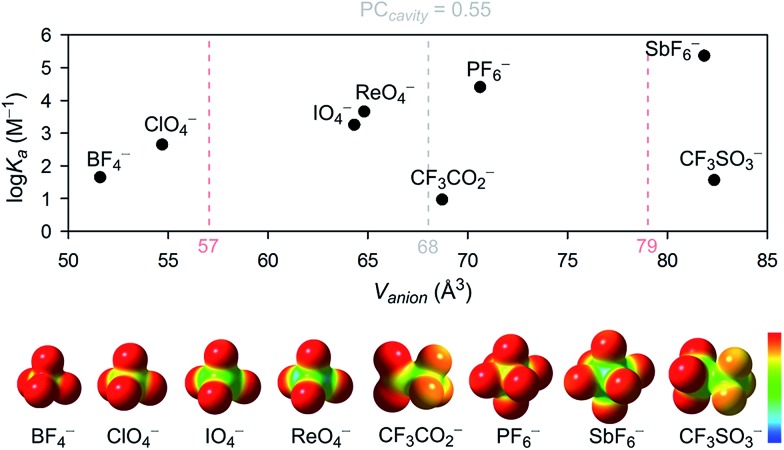
The association constant dependency on the anion size and the electrostatic surface potential of the studied anions. Surface potential calculated using Gaussian 09,^46^ visualized using GaussView5,^47^ red to blue surface color range spans from –0.2 to 0.2. Pale red dashed lines represent the 68 ± 11 Å^3^ anion volume range (PC = 0.55 ± 0.09).

For tetrahedral and octahedral anions, the affinity to the host grows exponentially with the increasing size of the guest, ranging from 48 M^–1^ for the smallest tested anion BF_4_^–^ to 250 000 M^–1^ for the largest tested octahedral anion SbF_6_^–^ (Table 1 and Fig. 4). The anion size dependency for anion binding has also been discussed by Sindelar and co-workers for a bambus[6]uril host in chloroform,^24^ with the highest selectivity towards ClO_4_^–^. Given the double-cone shape of the dodecabenzylbambus[6]uril host and the restricted diameter of the central cavity of 6-membered hemicucurbiturils, it seems that anions larger than perchlorate are bound away from the center of the bambus[6]uril macrocycle, inside the cone-shaped pockets formed by the extending substituents.^25,30^ With **cycHC[8]**, a single binding site is suggested by the crystal structures, with the anion in each case fully encapsulated in the center of the cavity. The selectivity of this 8-membered host is therefore determined by the parameters of its central cavity. Based on the crystal structures, the correlation between the binding strength and the size of the anion can arguably be ascribed to the number of host–guest interactions an anion can form simultaneously. Thus smaller anions BF_4_^–^ and ClO_4_^–^, which are able to form only one or two C–H···anion interactions in a given orientation, are bound with considerably lower affinities.

Surprisingly, however, the association constant for the binding of roughly octahedral CF_3_SO_3_^–^ is dramatically lower compared to the similarly sized octahedral guest SbF_6_^–^, regardless of the several interactions between the host and the encapsulated CF_3_SO_3_^–^ apparent in the crystal structure (Table S9†). Given the inherent symmetry of **cycHC[8]**, one can argue that the binding might be stronger with anions having the charge equally distributed over the surface. To assess whether the low binding affinity of CF_3_SO_3_^–^ is caused by the asymmetric charge distribution, a control experiment was conducted with CF_3_CO_2_^–^, similar in volume to PF_6_^–^, but with a charge distribution resembling that in CF_3_SO_3_^–^. The fact that CF_3_CO_2_^–^, which effectively templates the synthesis of **cycHC[8]** (in acetonitrile) and has been shown by diffusion NMR to bind to **cycHC[8]** in chloroform,^31^ does not bind to **cycHC[8]** in methanol (*K*_a_ < 10), indicates that the binding of anions to **cycHC[8]** in methanol is sensitive to charge distribution around the anion.

According to the range of optimal packing coefficients, 0.55 ± 0.09, the association constants within the tested group of tetrahedral and octahedral anions with roughly spherical charge distribution should follow a statistical distribution around the optimal guest fit at PC = 0.55. For **cycHC[8]**, with a cavity volume 123.0 Å^3^, the optimal guest volume should therefore centre in the range of 68 ± 11 Å^3^ (Fig. 4). However, the strongest association was in fact observed for SbF_6_^–^ (*V*_anion_ = 81.8 Å^3^), suggesting a shift from the optimal PC to higher values which might be due to the stabilizing effect of host–guest interactions with tighter-fitting guests like SbF_6_^–^ or the conformational flexibility of the macrocycle itself.

**Fig. 5:**
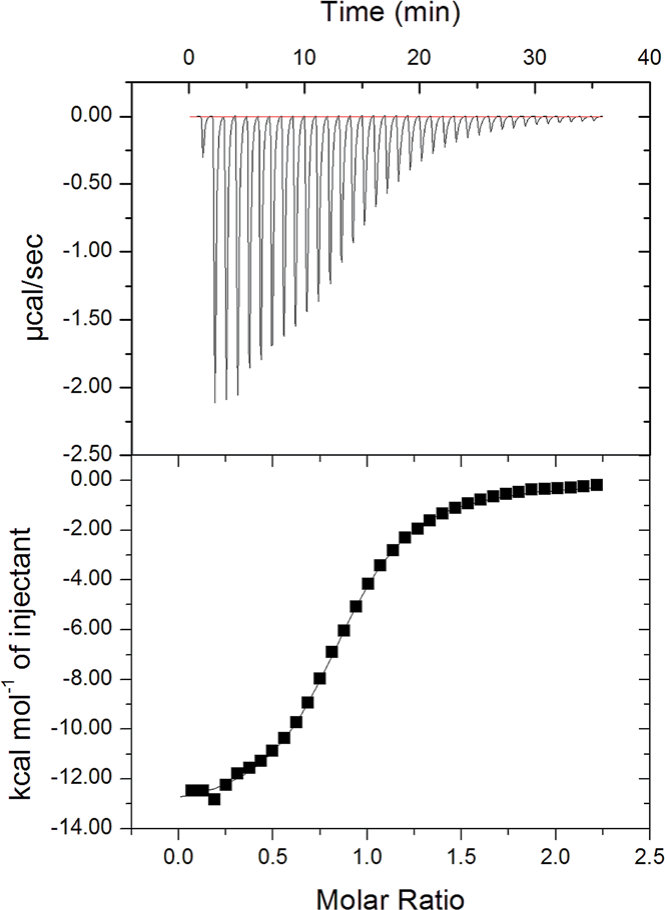
Raw thermogram (above) for SbF_6_^–^ titration with **cycHC[8]** and the binding isotherm (below) from the integrated thermogram fit using the one-site model.

Next, isothermal titration calorimetry (ITC) was used to determine thermodynamic parameters for the complexes of **cycHC[8]** with SbF_6_^–^ and PF_6_^–^ in methanol. The raw thermogram and the binding isotherm for SbF_6_^–^ are presented in Fig. 5 with the calculated parameters given in Table 2. The *K*_a_ values obtained by ITC were comparable with those obtained by NMR spectroscopy. Similarly to NMR, ITC showed higher affinity of **cycHC[8]** for SbF_6_^–^ than PF_6_^–^, with binding being exothermal, enthalpy driven and entropically disfavored for both. Comparing the two anions revealed that the binding of SbF_6_^–^ was accompanied by a greater change in both enthalpy and entropy. This is in line with tighter binding of the larger SbF_6_^–^ anion, giving rise to a stronger host–guest interaction (enthalpic term) but also a greater loss in degrees of freedom (entropic term). Generally, the thermodynamic profile of **cycHC[8]** binding resembles the behavior of other hemicucurbiturils.^37^

**Table 2:** Thermodynamic and kinetic parameters for the complexation with **cycHC[8]** at 298 K, all energies given in kJ mol^–1^

Guest	ITC titration			Δ*G*°			VT-NMR			
							Activation parameters for complex formation			Association rate constant^*a*^ *k* × 10^3^ s^–1^
	*K*_a_ (M^–1^)	Δ*H*°	*T*Δ*S*°	ITC titration	NMR titration	DFT calc.	Δ*H* ^‡^	*T*Δ*S* ^‡^	Δ*G* ^‡^	
NaSbF_6_	(1.02 ± 0.03) × 10^5^	–56.2 ± 0.3	–27.7	–28.5	–30.8	—	38.6	–13.4	51.9	2.6
NaPF_6_	(1.29 ± 0.04) × 10^4^	–43.8 ± 0.2	–20.4	–23.4	–24.5	–22	42.1	–5.5	47.5	17.5

^*a*^Determined at 291 K.

The complexation kinetics of normal cucurbiturils has been thoroughly^48–56^ studied and the exchange of neutral guests has been shown to proceed through a single step,^55,56^ while the complexation of cationic guests proceeds through a number of intermediates.^50–54^ To the best of our knowledge, the kinetics of anion binding by hemicucuribiturils has not been previously studied. A particularly interesting aspect of the **cycHC[8]** behavior is the conformational dynamics of guest encapsulation. Given the bulkiness of the encapsulated guests (Fig. 1), the conformation of the host clearly has to change considerably for the guest to pass through the narrow portals of **cycHC[8]**. The flexibility of the host is seemingly crucial to the encapsulation and, likewise, to the ejection of the guests, and the reaction pathway of host–guest complex formation was therefore studied computationally. Density functional theory (DFT) calculations were used to model the complexation of **cycHC[8]** with PF_6_^–^ utilizing COSMO solvation model for methanol.^57,58^

**Fig. 6:**
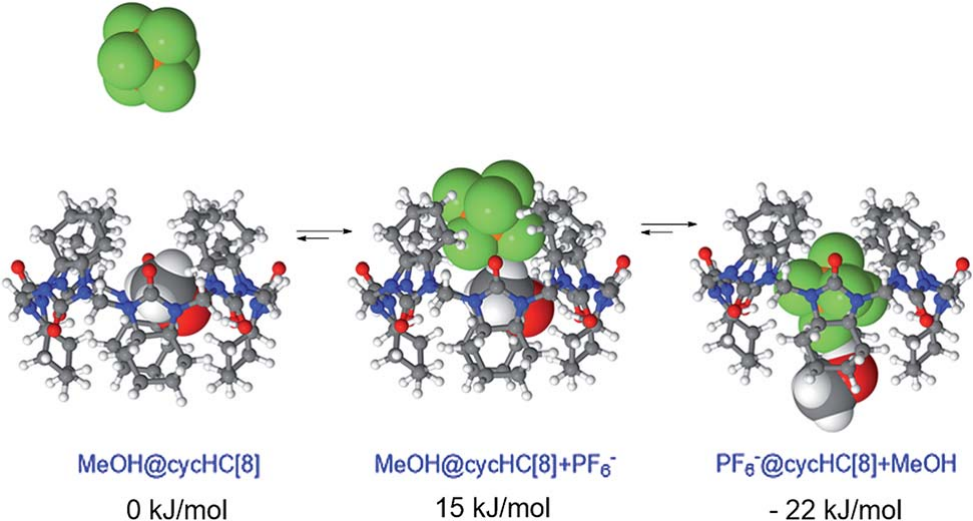
Minimum energy geometries of **cycHC[8]** complexes with PF_6_^–^ in MeOH and the associated relative Gibbs free energy values.

By positioning up to four methanol molecules inside the cavity of **cycHC[8]** we found that, at temperatures above 100 K, a single molecule of methanol is preferably accommodated within the cavity, close to its center. As the association constants derived from NMR titration with sodium and tetrabutylammonium PF_6_^–^ salts were very similar, cation influence was assumed to be negligible and was not studied. Modelling the exchange of methanol in MeOH@**cycHC[8]** with PF_6_^–^ showed the initial formation of a pre-complex with PF_6_^–^ at the portal of **cycHC[8]** (MeOH@**cycHC[8]** + PF_6_^–^; Fig. 6). Next, through a transition state involving the dissociation of a hydrogen bond between methanol and **cycHC[8]**, the inclusion complex PF_6_^–^@**cycHC[8]** is formed (the complexation reaction pathway is visualized in a video, ESI†). The DFT-derived Gibbs free energy difference indicates that the methanol-solvated cycHC[8] is 22 kJ mol^–1^ higher in energy compared to its inclusion complex with PF_6_^–^, which is in good agreement with the experimental Δ*G* values calculated from equilibrium constants obtained by NMR and ITC analyses (Table 2).

**Fig. 7:**
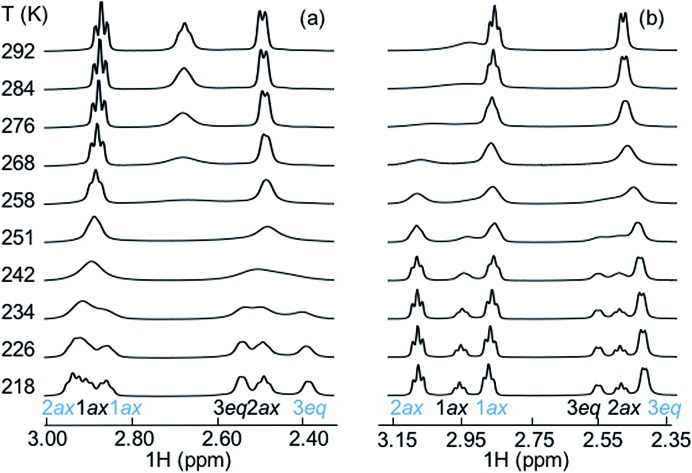
Evolution of proton resonances in the variable temperature NMR study (a) for PF_6_^–^ and (b) for SbF_6_^–^. The **cycHC[8]** and guest concentrations were 2.6 mM and 1.5 mM in MeOD solution, respectively. At 218 K, the hydrogen signals labelled with blue arise from the host–guest complex and black ones from the free host.

Additional experimental insight into the kinetics of the complexation and the reaction pathway was gained by variable temperature NMR (VT-NMR) studies of SbF_6_^–^ and PF_6_^–^, using a 2 : 1 host-to-guest ratio (Fig. 7). The complexation reaction order was determined by dilution experiments near the coalescence temperature (241 K and 253 K for SbF_6_^–^ and PF_6_^–^, respectively).

Reaction rates remained constant upon dilution, indicating that the complexation process follows first order kinetics, characteristic for a unimolecular reaction. This suggests that the overall complexation reaction occurs *via* a low-energy pre-complex. Moreover, the complexation reaction rate constants for both SbF_6_^–^ and PF_6_^–^ (Table 2) were determined, allowing us to derive the activation parameters (Table 2) using the Eyring equation (for details, see ESI†). As expected, the complexation rate constant was an order of magnitude higher for PF_6_^–^ than for the larger SbF_6_^–^, also in good agreement with our initial solution studies by NMR. The entropy of activation of the complexation is negative for both anions, as expected for the host and guest forming one host–guest complex. Other entropic contributions that play a role in the host–guest formation reaction are the entropic penalty of orientation of the guest within the macrocycle and the desolvation of the cavity of the host and the collapse of the methanol cluster around the chaotropic guests.

Both the computational and kinetic studies propose the existence of pre-complexes, although the computations suggest them to be higher in energy than the precursors, whereas the VT-NMR results require this pre-complex to be more stable than the starting components. This reflects a complicated complexation reaction pathway that includes several steps. We propose that either the anion pre-complex formation or the reorganization of the methanol solvation shell around anions and the macrocycle play an important role, which merits further investigation.

## Conclusions

The inherently chiral (*all-R*)-cyclohexanohemicucurbit[8]uril, **cycHC[8]**, is the first example of an octameric macrocycle of the hemicucurbituril family acting as a neutral host that fully encapsulates anions as 1 : 1 complexes in gas, solution and solid state. The binding affinity strongly depends on the size, shape and charge distribution of the anion. Crystallographic studies show the importance of the shape fit between the **cycHC[8]** cavity and the anion guest, with the octahedral guests bound in a perfectly ordered manner, while the tetrahedral guests exhibited various degrees of disorder. Moreover, due to the unique size and shape of **cycHC[8]**, the large, octahedrally shaped SbF_6_^–^ was found to be the most tightly bound guest, closely followed by PF_6_^–^. On the other hand, binding of the similarly sized CF_3_SO_3_^–^ and CF_3_CO_2_^–^ was around four orders of magnitude weaker, which could be ascribed to their asymmetric charge distribution limiting the number of hydrogen bonds that can be formed simultaneously. The complexation was found to proceed through the formation of a pre-complex, accompanied by the ejection of a solvent molecule from the **cycHC[8]** cavity and involving a large movement of the portals during the encapsulation.

This work, besides being the first comprehensive anion-binding study on an eight-membered hemicucurbituril-type macrocycle, also demonstrates the unique anion binding properties of a macrocyclic host easily accessible through a simple templated synthetic protocol.^31^ Moreover, it proposes a pathway to and encourages the preparation of new hemicucurbiturils for anion binding and transport, catalysis in confined space and other supramolecular applications.

## Author contribution

The manuscript was written through contributions of all authors.
