# Supplementary material for: Chiral hemicucurbit[8]uril as an anion receptor: selectivity to size, shape and charge distribution
Source: Chem Sci. 2016 Nov 30;8(3):2184–90. doi: 10.1039/c6sc05058a (PMC5477839; doi:10.1039/c6sc05058a)
Supplement: Supplementary file 2 [file SC-008-C6SC05058A-s002.pdf]

Electronic Supplementary Material (ESI) for Chemical Science.
This journal is © The Royal Society of Chemistry 2016

Electronic Supplementary Information for

Chiral Hemicucurbit[8]uril as Anion Receptor: Selectivity to Size, Shape and Charge Distribution

Sandra Kaabel,[a,b] Jasper Adamson,[c] Filip Topić,[b] Anniina Kiesilä,[b] Elina Kalenius,[b] Mario Öeren,[a] Mart Reimund,[a] Elena Prigorchenko,[a] Aivar Lõokene,[a] Hans J. Reich,[d] Kari Rissanen[b,*] and Riina Aav[a,*]

a. *Department of Chemistry, Tallinn University of Technology, Akadeemia tee 15, 12618 Tallinn, Estonia.*
b. *University of Jyvaskyla, Department of Chemistry, Nanoscience Center, P.O. Box. 35, FI-40014 Jyvaskyla, Finland.*
c. *National Institute of Chemical Physics and Biophysics, Akadeemia tee 23, 12618 Tallinn, Estonia*
d. *Department of Chemistry, University of Wisconsin, Madison, WI 53706, USA*

* riina.aav@ttu.ee, kari.t.rissanen@jyu.fi

Table of Contents

List of abbreviations 2
1. Mass Spectrometry 3
ESI-MS experimental 3
Complexation and competition experiments 3
CID experiments 6
2. Single Crystal X-ray diffraction analysis 8
Materials and methods 8
General remarks 8
Crystallographic details for TBA(PF_6@cycHC[8]) · 1.75CH_3OH 8
Crystallographic details for TBP(SbF_6@cycHC[8]) · 2CH_3OH 9
Crystallographic details for TBA(BF_4@cycHC[8]) · 2CH_3OH 9
Crystallographic details for TBA(ClO_4@cycHC[8]) · 2CH_3OH 10
Crystallographic details for TBP(IO_4@cycHC[8]) · 2CH_3OH 10
Crystallographic details for TBA(ReO_4@cycHC[8]) · 2CH_3OH 10
Crystallographic details for TBA(CF_3SO_3@cycHC[8]) · 2CH_3OH 11
Analysis of Hirshfeld surfaces and host-guest interactions 13
3. Host guest binding studies in solution state 21
Determination of the stoichiometry of the cycHC[8] inclusion complexes 21
Determination of the association constants 22
General remarks 22
Stacked spectra of the titration experiments and fitted isotherms of the titration data 23
ITC measurements 34
Variable temperature NMR for NaSbF6 and NaPF6 inclusion complexes 37
General methods 37
Association-dissociation reaction order 37
WINDNMR fits 39

4. Computational details 42
The formation of PF_6^-@cycHC[8] complex in MeOH 42
Number of solvent molecules in the cavity of cycHC[8] 42
Solvent molecule ejection and PF_6^- encapsulation to cycHC[8] 48
Optimized minima of the reaction 49
Optimized geometries 53
5. References 68

List of abbreviations

cycHC[8] – cyclohexanohemicucurbit[8]uril, in this context (*all-R*)-cyclohexanohemicucurbit[8]uril

TBA – tetrabutylammonium ion

TBP – tetrabutylphosphonium ion

ESI-MS – electrospray ionization mass spectrometry

CID – collision-induced dissociation

CE – collision energy

sof – site occupancy factor

ITC – isothermal titration calorimetry

VT-NMR – variable temperature nuclear magnetic resonance spectroscopy

1. Mass Spectrometry

ESI-MS experimental

Mass spectrometry experiments were performed with AB Sciex QSTAR Elite ESI-Q-TOF mass spectrometer, equipped with an API 200 TurboIonSpray ESI source from AB Sciex. Nitrogen was used as drying and nebulization gas. The parameters were optimized to get the maximum abundance of ions under study. The measurements and data handling was done with Analyst® QS 2.0 software. The ions were characterized according to comparison of theoretical and experimental *m/z* values and isotopic patterns calculated on basis of natural abundances of elements.

Samples were prepared using HPLC solvents. From host 1 mM stock solution was made in methanol. From salts 2 or 5 mM stock solutions were prepared in methanol. All samples were prepared with 10 μM concentration and 1:1 host:guest ratio in methanol. The samples were injected into the ESI source with 5 μl flow rate and spectra were externally calibrated using sodium trifluoroacetate.

Competition experiments were performed with 1:1:1 ratio using two competing anions with same counter cation (Na^+ or TBA^+) at the time. Competition experiments were carried out on five different samples, which all were measured five times. The overall variance was calculated from the standard deviation of sampling and the standard deviation of the measurement ($s^{tot} = s^1 + s^2$). Measurements or samples were rejected if the average deviation of a suspect value from the mean was four or more times the average deviation of the retained values.

In CID experiments the ions were isolated and activated by CE values from 5 to 55 V. Nitrogen was used as a collision gas with 5 bar pressure. The dissociation was followed as a function of CE value and dissociation graphs were fitted to sigmoidal. For studied ions $CE^{50\%}$ values were defined using OriginPro 2015 software.

Complexation and competition experiments

Complexation of **cycHC[8]** was monitored on (–)ESI-MS mode in methanol, where only free anion and 1:1 host:guest complexes were observed. Based on complexation experiments, the affinity of the host was studied in competition experiments, where two anions with the same counter cation were added to the same sample. The order in affinity was determined as follows: $SbF_6^- \approx PF_6^- > ReO_4^- > ClO_4^- > SCN^- > BF_4^- > HSO_4^- > CF_3SO_3^- > H_2PO_4^- > AcO^-$. The competition between SbF_6^- and PF_6^- was performed several times, but no clear preference was observed.

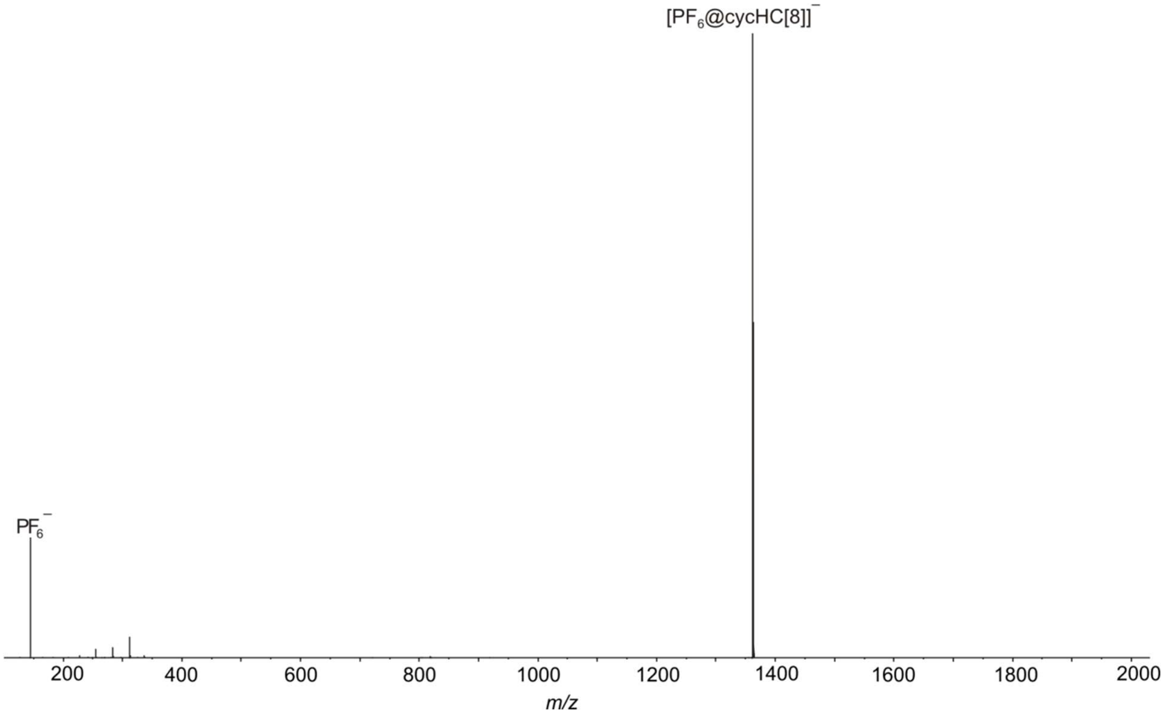

Figure S1. (–)ESI-MS spectrum of **cycHC[8]** + $TBAPF_6$ (10 µM in MeOH, 1:1 ratio).

Table S1. Observed host:guest complexes in ESI-MS analysis, their molecular formulas and theoretical and experimental *m*/*z*-values.

Ion	Molecular formula	m/z_{exp}	m/z_{theor}
$[PF_6@cycHC[8]]^-$	$C_{64}H_{96}N_{16}O_8PF_6$	1361.7292	1361.7244
$[SbF_6@cycHC[8]]^-$	$C_{64}H_{96}N_{16}O_8SbF_6$	1451.6632	1451.6545
$[ClO_4@cycHC[8]]^-$	$C_{64}H_{96}N_{16}O_{12}Cl$	1315.7169	1315.7088
$[ReO_4@cycHC[8]]^-$	$C_{64}H_{96}N_{16}O_{12}Re$	1467.7202	1467.6962
$[SCN@cycHC[8]]^-$	$C_{65}H_{96}N_{17}O_8S$	1274.7332	1274.7354
$[BF_4@cycHC[8]]^-$	$C_{64}H_{96}N_{16}O_8BF_4$	1303.7698	1303.7642
$[HSO_4@cycHC[8]]^-$	$C_{64}H_{97}N_{16}O_{12}S$	1313.7138	1313.7198
$[CF_3SO_3@cycHC[8]]^-$	$C_{65}H_{96}N_{16}O_{11}F_3S$	1365.7078	1365.7123
$[H_2PO_4@cycHC[8]]^-$	$C_{64}H_{98}N_{16}O_{12}P$	1313.7294	1313.7293
$[AcO@cycHC[8]]^-$	$C_{66}H_{99}N_{16}O_{10}$	1275.7764	1275.7736
$[Br@cycHC[8]]^-$	$C_{64}H_{96}N_{16}O_8Br$	1295.7060	1295.6786
$[Cl@cycHC[8]]^-$	$C_{64}H_{96}N_{16}O_8Cl$	1251.7939	1251.7291
$[I@cycHC[8]]^-$	$C_{64}H_{96}N_{16}O_8I$	1343.7279	1343.6647
$[F@cycHC[8]]^-$	$C_{64}H_{96}N_{16}O_8F$	1235.7500	1235.7587
$[NO_3@cycHC[8]]^-$	$C_{64}H_{96}N_{17}O_{11}$	1278.7802	1278.7481
$[NO_2@cycHC[8]]^-$	$C_{64}H_{96}N_{17}O_{10}$	1262.7910	1262.7532

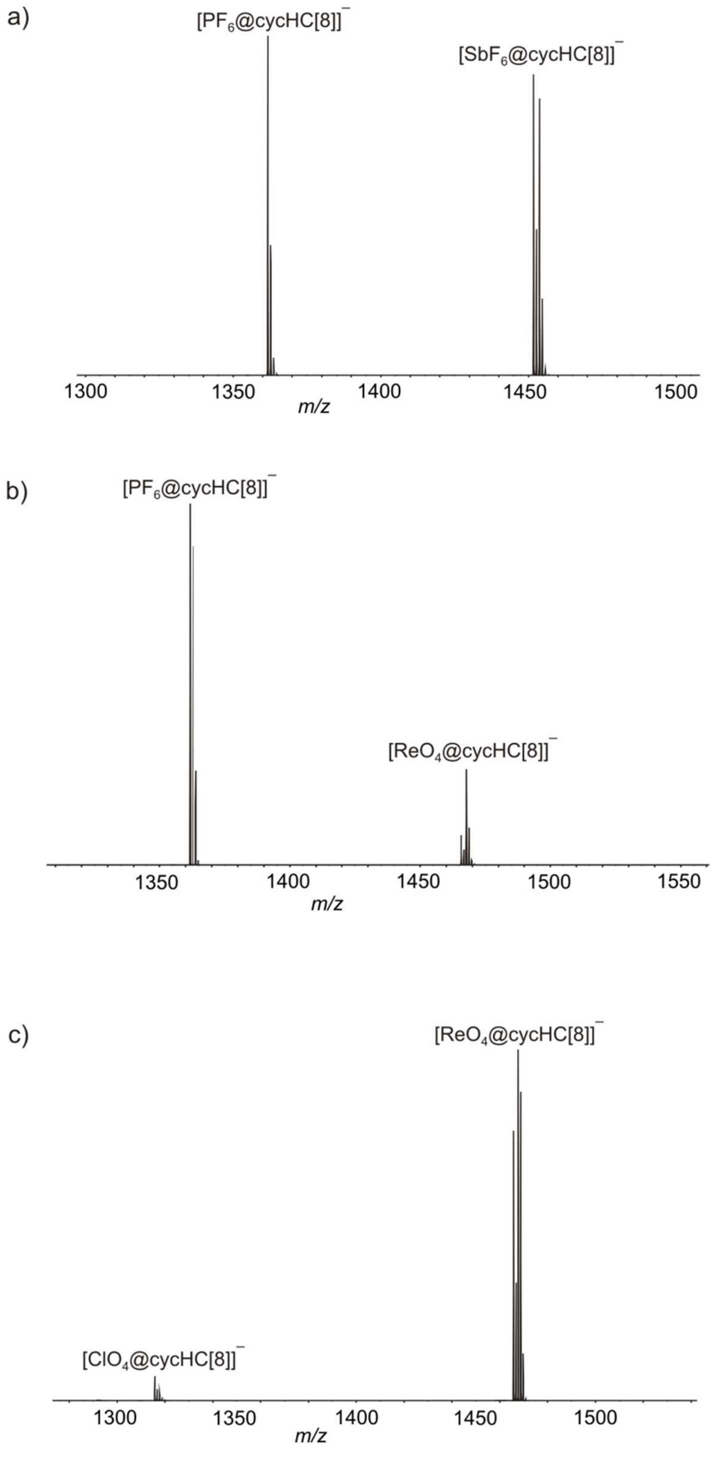

Figure S2. ESI-MS spectra from competition experiments: a) competition between $NaPF_6$/$NaSbF_6$ b) $TBAPF_6$/$TBAReO_4$ and c) $TBAClO_4$/$TBAReO_4$.

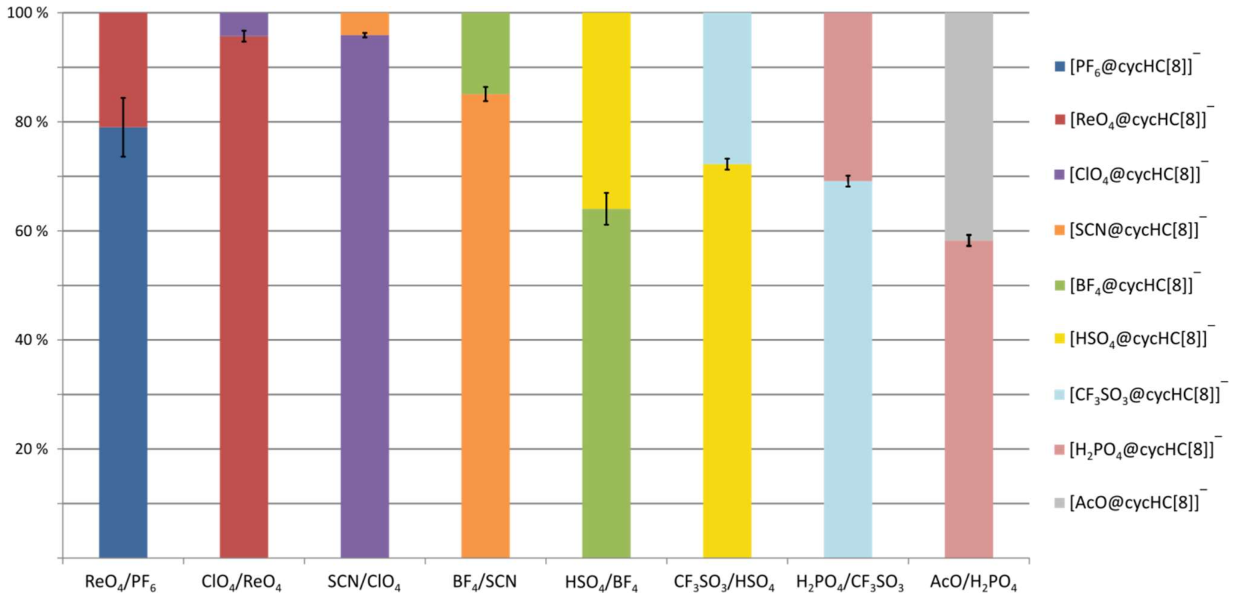

Figure S3. Results from bilateral competition experiments. Relative intensities of competing complexes from individual competition experiments presented.

CID experiments

CID experiments were performed for $[cycHC[8]+PF_6]^-$, $[cycHC[8]+SbF_6]^-$, $[cycHC[8]+ReO_4]^-$, $[cycHC[8]+ClO_4]^-$, $[cycHC[8]+CF_3SO_3]^-$ and $[cycHC[8]+BF_4]^-$ complexes. All isolated complexes dissociated through same pathway with elimination of an anion. $CE^{50\%}$ values, which represent the relative thermal energy that is needed for a complex to dissociate to its half intensity, were calculated from dissociation curves.

Tetrahedral anions with smaller packing coefficient seem to form kinetically more stable complexes with **cycHC[8]**. This is most probably due to size fit into the cavity. While anions with smaller packing coefficient have void space around them, higher kinetic activation is needed for these complexes to dissociate. Respectively, guests with higher packing coefficient are more sensitive for thermal activation, and lack of void space inside the cavity results in low-energy dissociation.

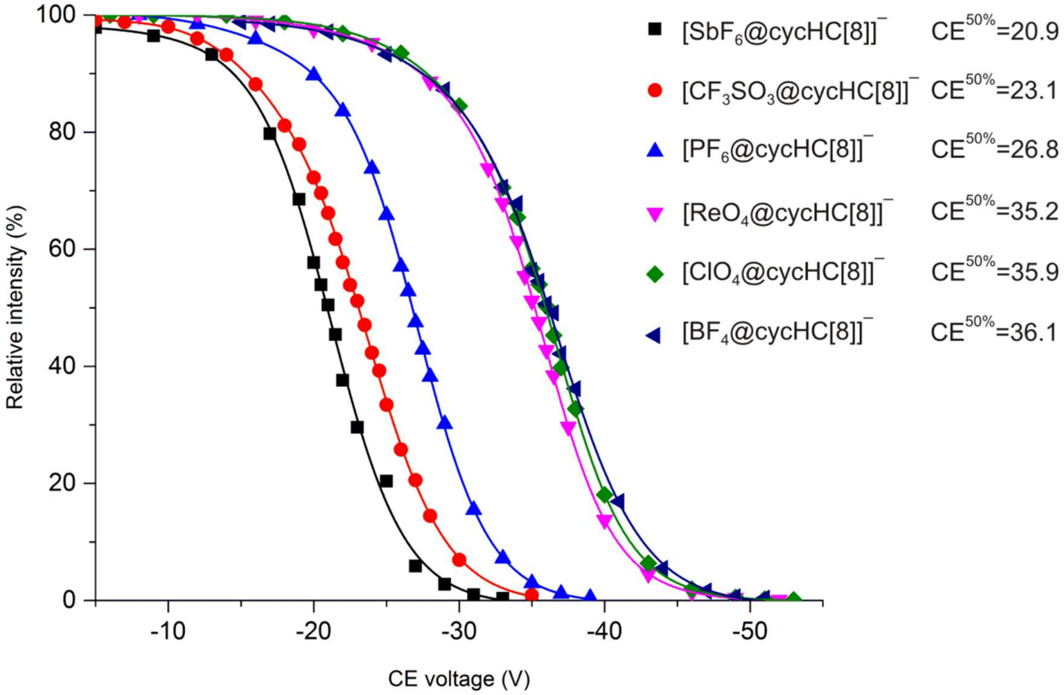

Figure S4. Dissociation curves for isolated ions studied in CID experiments and their $CE^{50\%}$ values.

2. Single Crystal X-ray diffraction analysis

Materials and methods

(*all-R*)-**cycHC[8]** was synthesized as described in literature.[1] Commercial reagents were purchased as reagent-grade and used without further purification.

General remarks

Single crystal X-ray diffraction data was collected on Agilent SuperNova Dual diffractometer, equipped with an Atlas detector and an Oxford Cryostream cooling system, using mirror-monochromatized Cu-$K\alpha$ radiation (1.54178Å), with the exception of data for TBP(IO_4@**cycHC[8]**) which was collected using mirror-monochromatized Mo-$K\alpha$ radiation (0.71073Å). *CrysAlisPro*[2] was used for data collection, processing and for applying numerical absorption correction[3]. Structures were solved using *SHELXT*[4]. The structures were refined by full-matrix least-squares method against F^2 with *SHELXL-2014*[4] through *WinGX*[5] and *OLEX2*[6] program packages. All non-hydrogen atoms were refined with anisotropic atomic displacement parameters, hydrogen atoms were calculated to their optimal positions and treated as riding on their parent carbon and oxygen atoms, with U_{iso}(H) = 1.2U_{iso}(C) for CH and CH_2; U_{iso}(H) = 1.5U_{iso}(C or O) for CH_3 and OH. Appropriate restraints were applied to the geometry and atomic displacement parameters of the atoms throughout the structures. Some reflections obscured by the beam stop were omitted from the refinements. The absolute configuration of **cycHC[8]** was confirmed based on the anomalous dispersion effects. The figures were drawn using the programs *Mercury*[7] and POV-Ray[8]. The Hirshfeld surfaces between the host and guest were visualized using *CrystalExplorer*[9] software. The crystallographic data is deposited with the Cambridge Crystallographic Data Centre (CCDC 1514736-1514741, 1521388) and can be obtained free of charge from The Cambridge Crystallographic Data Centre *via* www.ccdc.cam.ac.uk/data_request/cif.

Crystallographic details for TBA(PF_6@cycHC[8]) · 1.75CH_3OH

Single crystals of the complex were obtained from a methanol solution of **cycHC[8]** with twofold excess of TBAPF_6 by slow evaporation of the solvent. The resulting colourless plate-like crystals were found to be of an inclusion complex of the PF_6^- in **cycHC[8]**, with the tetrabutylammonium ion positioned between the capsule-like moieties. TBA^+ was refined in two separate disorder components, with relative *sof* of the respective disorder components allowed to refine freely. The resulting relative *sof* of the disorder components was 0.729(3)/0.271(3). The asymmetric unit contains two solvent molecules, one of which was modelled as disordered between two discreet orientations, with relative occupancies 0.711(6)/0.289(6). The other methanol molecule was refined with reduced occupancy (0.75). The compound was found to crystallize in a non-centrosymmetric space group $P2_12_12_1$, with four TBA(PF_6@**cycHC[8]**) moieties and eight solvent molecules in the unit cell.

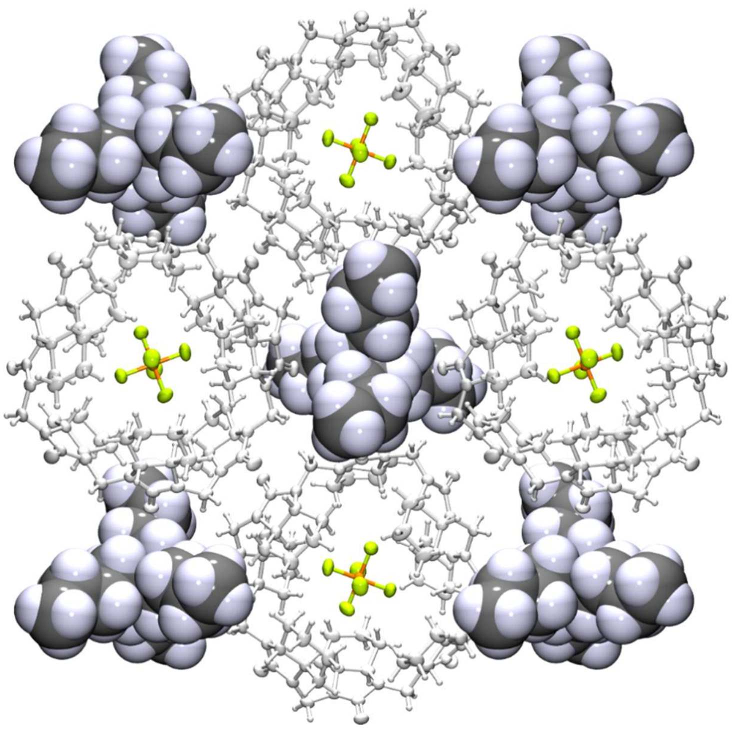

Figure S5. Packing of the TBA(PF_6@**cycHC[8]**) · 1.75CH_3OH along c-axis. Solvent molecules are omitted for clarity.

***Crystallographic details for* TBP(SbF_6@cycHC[8]) · 2CH_3OH**

Due to the commercial unavailability of tetrabutylammonium or tetrabutylphosphonium hexafluoroantimonate salts, an amount of TBPSbF_6 sufficient for threefold excess over **cycHC[8]** was prepared ahead of crystallization. For this equimolar amounts of NaSbF_6 and TBPBr were weighed out and dissolved separately in distilled water. Upon mixing the two aqueous solutions a white precipitate formed. The precipitate was extracted from the suspension by repeated washing with by CH_2Cl_2. The organic phase was collected and subsequently dried, resulting in a white solid. Neither the purity nor the yield of the product was determined. The solid was dissolved in methanol and added to **cycHC[8]**. The solution was sonicated and heated for all of the **cycHC[8]** to dissolve. When the clear solution was left to cool down, small colourless block-like single crystals of TBP(SbF_6@**cycHC[8]**) · 2CH_3OH formed. The compound was found to crystallize in monoclinic space group $P2_1$, with unit cell dimensions very close to orthorhombic [β = 90.0578°]. The crystal exhibited twinning by pseudo-merohedry, imitating orthorhombic Laue symmetry. Twin law, a two-fold rotation around the crystallographic *c*-axis (−100 0−10 001), was found using the TwinRotMat tool of PLATON[10] and applied in the refinement. The fraction of the minor twin component refined to 0.2056(6). The unit cell contains four TBP(SbF_6@**cycHC[8]**) moieties together with 8 solvent molecules.

***Crystallographic details for* TBA(BF_4@cycHC[8]) · 2CH_3OH**

Single crystals of the complex were obtained from a methanol solution of **cycHC[8]** with about twofold excess of TBABF_4 upon slow evaporation of the solvent. The inclusion complex crystallized in an orthorhombic space group $P2_12_12_1$, with four units of TBA(BF_4@**cycHC[8]**) together with eight solvent molecules in the unit cell. The tetrafluoroborate anion was refined in four separate disorder components, with *sof* of their respective relative occupancies refined freely. The resulting relative *sof* of the disorder components was 0.352(7)/0.163(7)/0.251(8)/0.23(8). The geometry of the disordered BF_4^- anions was restrained to be tetrahedral, with tying the 1,2- and 1,3- distances (B···F and F···B···F respectively) to a free variable which was then

refined. One of the two methanol molecules was modelled as occupying two discreet positions, with relative occupancies 0.601(6)/ 0.399(6).

***Crystallographic details for* TBA(ClO_4@cycHC[8]) · 2CH_3OH**

Single crystals of the complex were obtained from a methanol solution of **cycHC[8]** with an equimolar ratio of TBAClO_4 upon slow evaporation of the solvent. The inclusion complex crystallized in an orthorhombic space group $P2_12_12_1$, with four units of TBA(ClO_4@**cycHC[8]**) together with eight solvent molecules in the unit cell. The perchlorate anion was refined in four separate disorder components, with *sof* of their respective relative occupancies refined freely. The resulting *sof* of the disorder components was 0.291(8)/0.219(8)/0.252(8)/0.237(8). The geometry of the disordered ClO_4^- was restrained to be tetrahedral, with the 1,2- and 1,3- distances (C···O and O···C···O respectively) tied to a free variable, that was then allowed to refine. The disorder of one of the butyl groups of TBA was modelled between two discreet positions, with *sof* or the respective disorder components refined to 0.549(12)/0.451(12). One of the two methanol molecules in the asymmetric unit was modelled as occupying two discreet positions, with relative occupancies 0.857(7)/0.143(7).

***Crystallographic details for* TBP(IO_4@cycHC[8]) · 2CH_3OH**

An amount of TBPIO_4 sufficient for threefold excess over **cycHC[8]** was prepared ahead of crystallization. For this equimolar amounts of $NaIO_4$ and TBPBr were weighed out and dissolved separately in distilled water. Upon mixing the two aqueous solutions a white precipitate formed. The precipitate was extracted from the suspension by repeated washing with by CH_2Cl_2. The organic phase was collected and subsequently dried, resulting in a white solid. Neither the purity nor the yield of the product was determined. The solid was dissolved in methanol and added to **cycHC[8]**. The solution was sonicated and heated for all of the **cycHC[8]** to dissolve. When the clear solution was left to cool down, small colourless block-like single crystals of TBP(IO_4@**cycHC[8]**) · 2CH_3OH formed.

The compound was found to crystallize in monoclinic space group $P2_1$, with unit cell dimensions very close to orthorhombic [β = 90.0371(12)°]. The crystal exhibited twinning by pseudo-merohedry, imitating orthorhombic Laue symmetry. Twin law, a two-fold rotation around the crystallographic c-axis (−100 0−10 001) was found using the TwinRotMat tool of PLATON[10] and applied in the refinement. The fraction of the minor twin component refined to 0.3228(6). The unit cell contains four TBP(IO_4@**cycHC[8]**) moieties together with 8 solvent molecules. The two metaperiodide anions in the asymmetric unit were modelled in three disorder components each, with the occupancies of the respective disorder components refined freely, giving *sof* 0.431(3)/0.377(3)/0.192(3) and 0.346(3)/0.530(2)/0.123(2) respectively for components in the two anion sites. The geometry of the disorder components of IO_4^- was restrained to be tetrahedral, with the 1,2- and 1,3- distances (I···O and O···I···O respectively) tied to a free variable, which was then allowed to refine. One of the four methanol molecules in the asymmetric unit was modelled as occupying two discreet positions, with relative occupancies tied to a free variable which was then refined, giving *sof* 0.708(6)/0.292(6) for the disorder components.

***Crystallographic details for* TBA(ReO_4@cycHC[8]) · 2CH_3OH**

Single crystals of TBA(ReO_4@**cycHC[8]**) · 2CH_3OH were obtained from an equimolar solution of **cycHC[8]** and TBAReO_4 in methanol by slow evaporation of the solvent. The compound was found to crystallize in space group $P2_12_12_1$, with four TBA(ReO_4@**cycHC[8]**) moieties in the unit cell together with 8 methanol molecules. The perrhenate anion was modelled in two disorder components, with the occupancy of the disorder components

refined freely. The resulting relative *sof* of the disordered anion components was 0.597(4)/0.403(4). The geometry of the ReO_4^- was restrained to be tetrahedral, with the 1,2- and 1,3- distances (Re···O and O···Re···O respectively) tied to a free variable, which was then allowed to refine. One of the two methanol molecules in the asymmetric unit was modelled as disordered between two discreet orientations, with relative occupancies 0.64(5)/0.36(5). The other methanol molecule was also found to be disordered, but the minor component could not me modelled properly. The major component in this solvent site was modelled with 0.75 occupancy.

***Crystallographic details for* TBA(CF_3SO_3@cycHC[8]) · 2CH_3OH**

Single crystals of TBA(CF_3SO_3@**cycHC[8]**) · 2CH_3OH were obtained from an equimolar solution of **cycHC[8]** and TBACF_3SO_3 in methanol by slow evaporation of the solvent. The compound was found to crystallize in space group $P2_12_12_1$, with four TBA(CF_3SO_3@**cycHC[8]**) moieties in the unit cell together with 8 methanol molecules. The encapsulated trifluoromethanesulfonate anion was modelled in two disorder components, crossed to one another at an angle of about 67°. The relative occupancy of these was refined freely, resulting in *sof* 0.876(3)/0.124(3). The geometry of the $CF_3SO_3^-$ was restrained to be more symmetrical and same for the two modelled disorder components. Two additional disorder components are suggested by looking at the anisotropic displacement parameters of the modelled $CF_3SO_3^-$. The ADPs of the sulphur atoms are unexpectedly large while the carbon atoms have relatively small ADPs, indicating that each of the $CF_3SO_3^-$ orientations probably has an additional overlapping disorder component flipped 180 degrees respective to it. However attempts to refine these additional disorder components were not successful as the geometry of the flipped minor components was severely affected by their overlap with the major disorder components. One tetrabutyl group of the TBA^+ was seen to be disordered, and was therefore modelled in two disorder components. The relative occupancy refined to 0.563(12)/0.437(12) for the disorder components. One of the two methanol molecules in the asymmetric unit was modelled as disordered between two discreet orientations, with relative occupancies 0.738(6)/0.262(6). Restraints were applied to the geometry of the solvent molecules. Restraints were also applied to the ADPs of the disordered parts of the structure. Some reflections, obscured by the beam stop, were omitted from the refinement.

Table S2. Crystallographic data for the inclusion complexes *Cation*(*Anion*@**cycHC[8]**) · *n*CH_3OH

Identification code	TBA(PF_6@**cycHC[8]**)	TBP(SbF_6@**cycHC[8]**)	TBA(BF_4@**cycHC[8]**)	TBA(ClO_4@**cycHC[8]**)	TBP(IO_4@**cycHC[8]**)	TBA(ReO_4@**cycHC[8]**)	TBA(CF_3SO_3@**cycHC[8]**)
CCDC number	**1514736**	**1514737**	**1514738**	**1521388**	**1514740**	**1514739**	**1514741**
Empirical formula	$C_{81.75}H_{139}F_6N_{17}O_{9.75}P$	$C_{82}H_{140}F_6N_{16}O_{10}PSb$	$C_{82}H_{140}BF_4N_{17}O_{10}$	$C_{82}H_{140}ClN_{17}O_{14}$	$C_{82}H_{140}IN_{16}O_{14}P$	$C_{81.75}H_{139}N_{17}O_{13.75}Re$	$C_{83}H_{140}F_3N_{17}O_{13}S$
Formula weight	1661.06	1776.81	1610.91	1623.51	1731.96	1766.29	1673.17
Temperature/K	123.01(10)	123.01(10)	123.01(10)	123.01(10)	120.00(10)	123.01(10)	123.01(10)
Crystal system	orthorhombic	monoclinic	orthorhombic	orthorhombic	monoclinic	orthorhombic	orthorhombic
Space group	$P2_12_12_1$	$P2_1$	$P2_12_12_1$	$P2_12_12_1$	$P2_1$	$P2_12_12_1$	$P2_12_12_1$
a/Å	20.2513(2)	20.20836(17)	20.03081(12)	20.19077(10)	20.1263(3)	20.1641(3)	20.27229(12)
b/Å	20.7166(3)	21.3780(2)	20.92663(13)	20.79244(11)	21.3089(3)	20.8019(3)	20.73280(13)
c/Å	21.07611(18)	20.70075(19)	20.97159(13)	20.95498(11)	20.7811(2)	21.1254(3)	21.02695(11)
α/°	90	90	90	90	90	90	90
β/°	90	90.0578(8)	90	90	90.0371(12)	90	90
γ/°	90	90	90	90	90	90	90
V/Å^3	8842.22(17)	8943.00(14)	8790.81(9)	8797.22(8)	8912.4(2)	8861.1(2)	8837.65(9)
Z	4	4	4	4	4	4	4
D_{calc}/g cm^{-3}	1.248	1.320	1.217	1.226	1.291	1.324	1.258
μ/mm^{-1}	0.914	3.25	0.70	0.950	0.446	3.239	0.946
F(000)	3582.0	3776	3488	3520.0	3696.0	3734.0	3616.0
Crystal size/mm^3	0.2794×0.2509×0.0706	0.1754×0.1528×0.1056	0.5093×0.1537×0.1251	0.4083×0.3874×0.3281	0.5041×0.4425×0.3579	0.2767×0.2026×0.1869	0.6163×0.2939×0.2541
Radiation, λ/Å	1.54184 (CuK_α)	1.54184 (CuK_α)	1.54184 (CuK_α)	1.54184 (CuK_α)	0.71073 (MoK_α)	1.54184 (CuK_α)	1.54184 (CuK_α)
2Θ range /°	6.052 to 146.87	6.0 to 148.6	6.102 to 148.54	7.42 to 148.396	5.882 to 58.736	6.06 to 148.66	6.056 to 148.602
Index ranges	$-24 \le h \le 23$,<br>$-24 \le k \le 25$,<br>$-18 \le l \le 26$	$-21 \le h \le 24$,<br>$-24 \le k \le 26$,<br>$-25 \le l \le 25$	$-24 \le h \le 14$,<br>$-26 \le k \le 25$,<br>$-25 \le l \le 26$	$-24 \le h \le 25$,<br>$-25 \le k \le 23$,<br>$-23 \le l \le 25$	$-27 \le h \le 26$,<br>$-28 \le k \le 27$,<br>$-28 \le l \le 28$	$-22 \le h \le 24$,<br>$-24 \le k \le 25$,<br>$-25 \le l \le 26$	$-20 \le h \le 25$,<br>$-25 \le k \le 25$,<br>$-24 \le l \le 26$
Reflections collected	27726	66781	70925	35570	118166	35614	65288
Independent reflections	16875 [R_{int} = 0.0195, R_{sigma} = 0.0265]	33880 [R_{int} = 0.038, R_{sigma} = 0.0455]	17694 [R_{int} = 0.0266, R_{sigma} = 0.0190]	17412 [R_{int} = 0.0169, R_{sigma} = 0.0204]	42566 [R_{int} = 0.0421, R_{sigma} = 0.0483]	17494 [R_{int} = 0.0206, R_{sigma} = 0.0266]	17735 [R_{int} = 0.0237, R_{sigma} = 0.0192]
Data/restraints/parameters	16875/2444/1231	33880/3641/2106	17694/2641/1196	17412/2420/1216	42566/4156/2266	17494/1990/1102	17735/2566/1176
Goodness-of-fit on F^2	1.031	1.039	1.061	1.037	1.041	1.033	1.040
Final R indexes [$I \ge 2\sigma(I)$]	R_1 = 0.0377,<br>wR_2 = 0.1002	R_1 = 0.0327,<br>wR_2 = 0.0931	R_1 = 0.0544,<br>wR_2 = 0.1574	R_1 = 0.0529,<br>wR_2 = 0.1516	R_1 = 0.0362,<br>wR_2 = 0.0836	R_1 = 0.0594,<br>wR_2 = 0.1643	R_1 = 0.0492,<br>wR_2 = 0.1432
Final R indexes [all data]	R_1 = 0.0412,<br>wR_2 = 0.1036	R_1 = 0.0334,<br>wR_2 = 0.0933	R_1 = 0.0553,<br>wR_2 = 0.1587	R_1 = 0.0538,<br>wR_2 = 0.1528	R_1 = 0.0385,<br>wR_2 = 0.0862	R_1 = 0.0613,<br>wR_2 = 0.1664	R_1 = 0.0502,<br>wR_2 = 0.1445
Largest diff. peak/hole /eÅ^{-3}	0.32/−0.26	0.83/−0.89	0.68/−0.50	0.83/−0.46	0.40/−0.88	1.42/−2.08	0.64/−0.57
Flack parameter	0.016(8)	−0.0114(15)	0.00(3)	0.000(6)	-0.010(3)	-0.003(2)	0.044(5)

Analysis of Hirshfeld surfaces and host-guest interactions

Hirshfeld surfaces[11] provide a valuable tool for analysing the close contacts between moieties in the crystal structures. The surface is created around a molecule, where its electron density exceeds that from all the neighbouring molecules. In this work, the Hirshfeld surfaces were calculated for SbF_6^- (Figure S6), IO_4^- (Figure S7) and $CF_3SO_3^-$ (Figure S8), which represent the different shape of guest anions encapsulated. The program *CrystalExplorer*[9] was used to generate the Hirshfeld surfaces and to highlight the close contacts between the anion and the neighbouring host molecule (C−H···anion). The surfaces were mapped with d_{norm} where a red spot signifies areas with d(D-H···A) ≤ ∑r(*vdW*)[H, A].

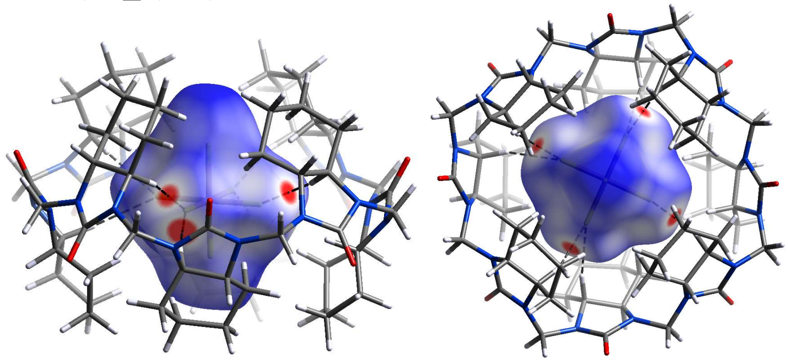

Figure S6. Hirshfeld surface for the encapsulated SbF_6^- anion, mapped with d_{norm} over the range −0.1 to 1.0. Close contacts where d(D-H···F) is shorter than ∑r(*vdW*)[H, F] are displayed with a dashed line

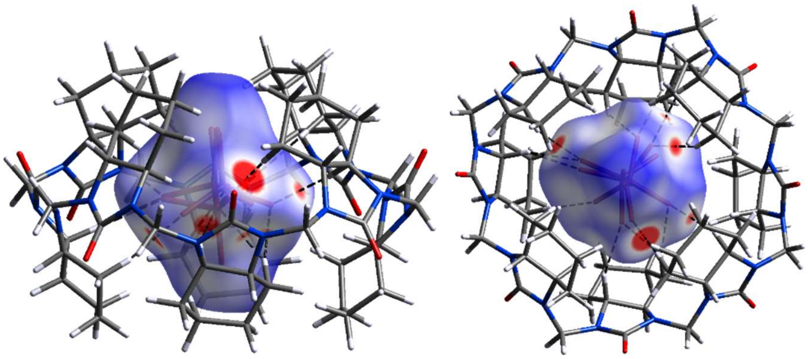

Figure S7. Hirshfeld surface for the encapsulated IO_4^- anion, mapped with d_{norm} over the range −0.1 to 1.0. The Hirshfeld surface is generated for all the three disorder components simultaneously. Close contacts where d(D-H···O) is shorter than ∑r(*vdW*)[H, O] by 0.10Å are displayed with a dashed line

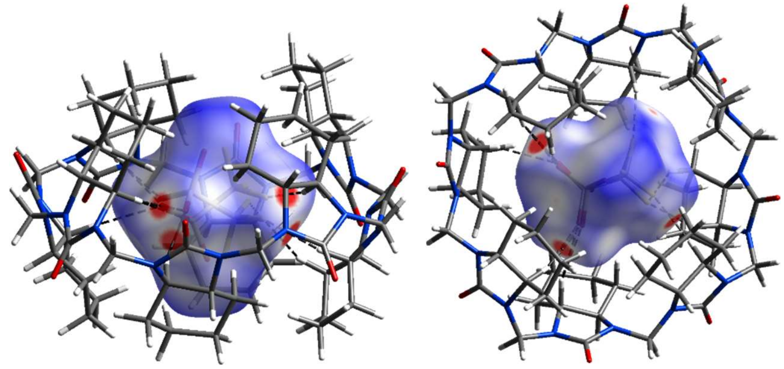

Figure S8. Hirshfeld surface for the encapsulated $CF_3SO_3^-$ anion, mapped with d_{norm} over the range −0.1 to 1.0. The Hirshfeld surface is generated for the two disorder components simultaneously. Close contacts where d(D-H···O) or d(D-H···F) is shorter by 0.10Å than ∑r(*vdW*)[H, O] or ∑r(*vdW*)[H, F] respectively are displayed with a dashed line

Shortest distances between the host molecule and the encapsulated anions were investigated using *Mercury 3.7*,[7] the result of which is shown in Tables S2 – S8. Distances shorter than the sum of van der Waals radii of hydrogen and the acceptor atom **∑r(*vdW*)[H, A]** are marked in black, as they indicate influential host-guest interactions. The denomination of atoms in the host molecule is as follows: equivalent monomeric units are assigned a suffix A-H (P) depending on the number of monomers in the asymmetric unit, atoms in the monomeric units are labelled as shown on Figure S9. Anions are labelled with suffixes when more than one moiety of the host-guest complex is present in the asymmetric unit or/and when numbering disorder components in one anion site.

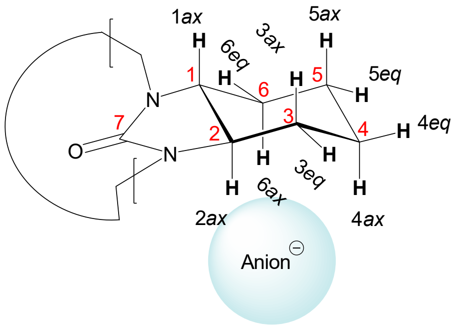

Figure S9. Atom labelling scheme shown on the monomeric unit of (*all-R*)-**cycHC[8]**. The placement of the model anion (light blue) indicates the side of the monomer facing the cavity. Numbers in red represent the numbering scheme of carbons.

Hexafluoroantimonate (SbF_6^-)

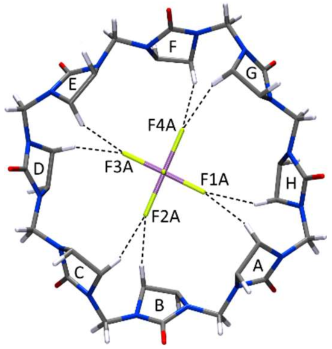

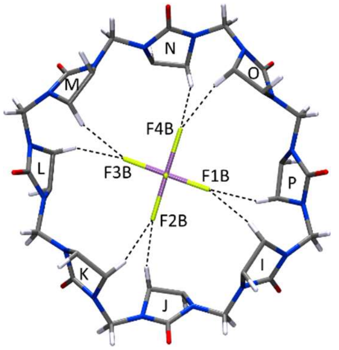

Table S3. Shortest *d*(D-H···A) distances from the crystal structure of TBP(SbF_6@**cycHC[8]**) · $2CH_3OH$, between the encapsulated SbF_6^- and the host. Two moieties of SbF_6@**cycHC[8]** are present in the asymmetric unit

D–H···A	d(H···A), Å	D(H···A) − ∑r(*vdW*)[H, A], Å
C2H–H2…F1A	2.646	0.09
**C2A–H2…F1A**	2.440	-0.12
**C2B–H2…F2A**	2.343	-0.22
**C2C–H2…F2A**	2.440	-0.12
**C2D–H2…F3A**	2.527	-0.03
**C2E–H2…F3A**	2.473	-0.09
**C2F–H2…F4A**	2.422	-0.14
**C2G–H2…F4A**	2.485	-0.08
C2P–H2…F1B	2.600	0.04
**C2I–H2…F1B**	2.403	-0.16
**C2J–H2…F2B**	2.315	-0.25
**C2K–H2…F2B**	2.383	-0.18
C2L–H2…F3B	2.689	0.13
**C2M–H2…F3B**	2.529	-0.03
**C2N–H2…F4B**	2.391	-0.17
**C2O–H2…F4B**	2.429	-0.13

Hexafluorophosphate (PF_6^-)

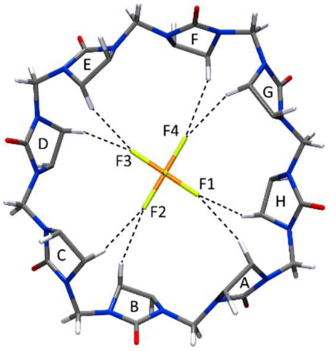

Table S4. Shortest *d*(D-H···A) distances from the crystal structure of TBA(PF_6@**cycHC[8]**) · 1.75CH_3OH, between the encapsulated PF_6^- and the host.

D−H···A	d(H···A), Å	D(H···A) − ∑r(*vdW*)[H, A], Å
C2H−H2H…F1	2.57	0.01
C2A−H2A…F1	2.69	0.13
C2B−H2B…F2	2.78	0.22
**C2C−H2C…F2**	2.55	-0.01
**C2D−H2D…F3**	2.51	-0.05
C2E−H2E…F3	2.65	0.09
C2F−H2F…F4	2.93	0.37
C2G−H2G…F4	2.66	0.10

Figure S10. Close contacts within PF_6@**cycHC[8]**. $(CH_2)_4$ groups are omitted for clarity, atoms involved in equivalent monomeric units are denoted with suffixes A-H.

Perrhenate (ReO_4^-)

Table S5. Shortest *d*(D-H···A) distances from the crystal structure of TBA(ReO_4@**cycHC[8]**) · 2CH_3OH, between the host and ReO_4^-. The disorder component denoted with K has *sof* 0.403(4), disorder component I has *sof* 0.597(4).

D−H···A	d(H···A), Å	D(H···A) − ∑r(*vdW*)[H, A], Å
**C6A−H6ax…O3K**	2.26	-0.35
**C2C−H2…O4K**	2.58	-0.03
C2D−H2…O4K	2.68	0.07
C6F−H2…O2K	2.63	0.02
C6F−H6ax…O2K	2.64	0.03
**C6A−H6ax…O4I**	2.57	-0.04
C6B−H6ax…O3I	2.62	0.01
C2E−H2…O2I	2.63	0.02
**C2H−H2…O4I**	2.51	-0.1
**C6H−H6ax…O4I**	2.57	-0.04

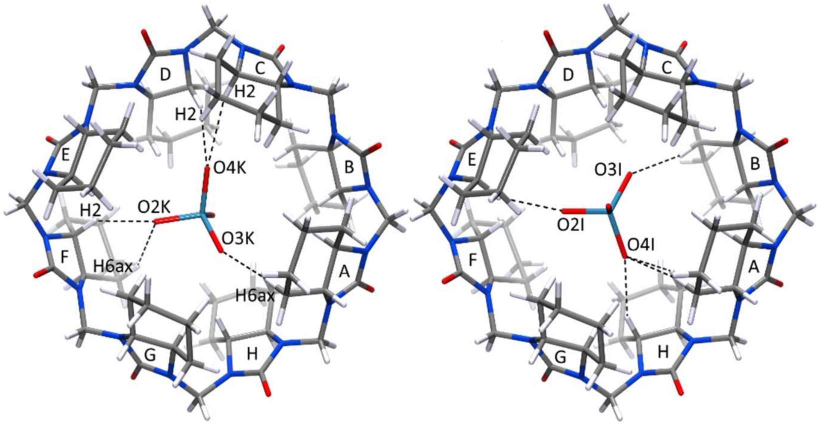

Figure S11. Close contacts within ReO_4@**cycHC[8]**. Atoms involved in equivalent monomeric units of the host are denoted with suffixes A-H. The contacts for the two disorder components of ReO_4^- are shown separately for clarity, but they occupy the same site with *sof* 0.403(4) for component K (left) and *sof* 0.597(4) for component I (right)

Periodate (IO_4^-)

Table S6 Shortest *d*(D-H···A) distances from the crystal structure of TBP(IO_4@**cycHC[8]**) · 2CH_3OH, between the host and IO_4^-. Two moieties of TBP(IO_4@**cycHC[8]**) are present in the asymmetric unit, IO_4^- has three disorder components in both sites, with *sof* 0.431(3)/0.192(3)/0.377(3) for K/L/M and 0.346(3)/0.530(2)/0.123(2) for O/P/Q.

D−H···A	**d(H···A), Å**	**D(H···A) − ∑r(*vdW*)[H, A], Å**
**C6B−H6ax…O3AK**	2.61	0
C6C−H6ax…O4AK	2.65	0.04
**C6D−H6ax…O4AK**	2.58	-0.03
**C2D−H2…O4AK**	2.44	-0.17
**C2F−H2…O2AK**	2.50	-0.11
**C2G−H2…O2AK**	2.47	-0.14
**C6H−H6ax…O4AL**	2.51	-0.1
**C2H−H2…O4AL**	2.51	-0.1
C2A−H2…O4AL	2.67	0.06
**C2C−H2…O2AL**	2.51	-0.1
**C6D−H6ax…O2AL**	2.57	-0.04
**C6F−H6ax…O3AL**	2.42	-0.19
C6G−H6ax…O4AL	2.64	0.03
C2H−H2…O3AM	2.70	0.09
**C2A−H2…O3AM**	2.58	-0.03
**C6A−H6ax…O3AM**	2.51	-0.1
**C6C−H6ax…O4AM**	2.30	-0.31
C6D−H6ax…O4AM	2.64	0.03
C6F−H6ax…O2AM	2.67	0.06
**C2F−H2…O2AM**	2.52	-0.09
C2G−H2…O2AM	2.63	0.02
**C2K−H2…O4AO**	2.60	-0.01
**C6N−H6ax…O2AO**	2.28	-0.33
C6P−H6ax…O3AO	2.62	0.01
**C2P−H2…O3AO**	2.55	-0.06
C2I−H2…O3AP	2.66	0.05
**C6I−H6ax…O4AP**	2.61	0
C2J−H2…O4AP	2.62	0.01
**C2M−H2…O2AP**	2.52	-0.09
**C6M−H6ax…O2AP**	2.54	-0.07
**C6N−H6ax…O2AP**	2.50	-0.11
C6O−H6ax…O3AP	2.69	0.08
C2I−H2…O3AQ	2.68	0.07
C6I−H6ax…O3AQ	2.65	0.04
**C6J−H6ax…O3AQ**	2.43	-0.18
**C6L−H6ax…O2AQ**	2.33	-0.28
**C2L−H2…O2AQ**	2.60	-0.01
**C2N−H2…O4AQ**	2.51	-0.1
**C2O−H2…O4AQ**	2.39	-0.22

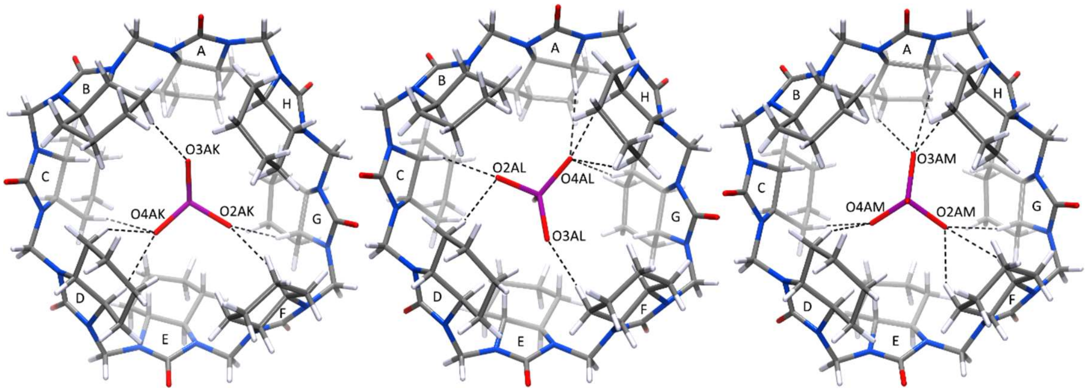

Figure S12. Close contacts within one moiety of IO_4@**cycHC[8]** in the asymmetric unit, (monomer suffixes A-H). The contacts for the three disorder components are shown separately for clarity, but they occupy the same site with *sof* 0.431(3) for component K (left), *sof* 0.192(3) for component L (centre) and *sof* 0.377(3) for component M (right)

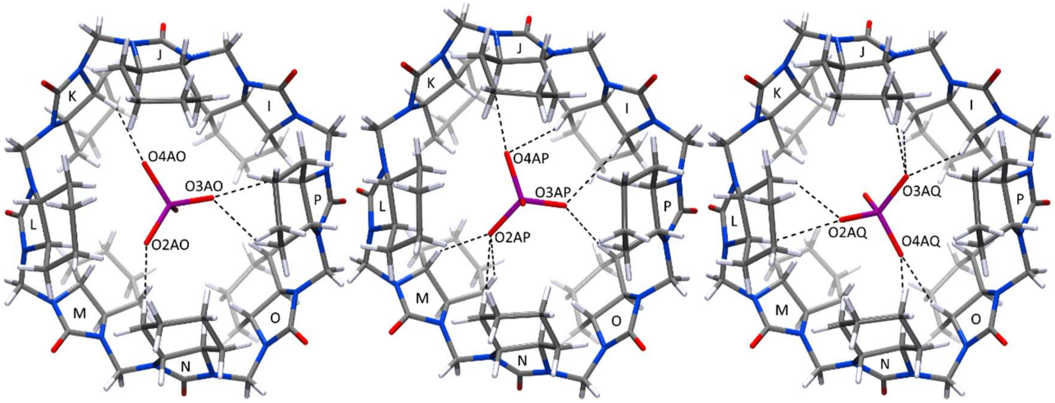

Figure S13. Close contacts within the second moiety of IO_4@**cycHC[8]** in the asymmetric unit, (monomer suffixes I-P). The contacts for the three disorder components are shown separately for clarity, but they occupy the same site with *sof* 0.346(3) for component O (left), *sof* 0.530(2) for component P (centre) and *sof* 0.123(2) for component Q (right)

Perchlorate (ClO_4^-)

Table S7. Shortest *d*(D-H···A) distances from crystal structure of TBA(ClO_4@**cycHC[8]**) · $2CH_3OH$, between the host and ClO_4^-. ClO_4^- has four disorder components in the binding site, with *sof* 0.291(8)/0.252(8)/0.219(8)/0.237(8) for XA/XB/XC/XD respectively.

D−H···A	d(H···A), Å	D(H···A) − ∑r(*vdW*)[H, A], Å
**C6C−H6ax…O3XA**	2.46	-0.15
**C6D−H6ax…O3XA**	2.43	-0.18
C2B−H2…O2XB	2.67	0.06
**C2C−H2…O2XB**	2.52	-0.09
C6D−H6ax…O2XB	2.66	0.05
**C6G−H6ax…O1XB**	2.48	-0.13
C6H−H6ax…O1XB	2.7	0.09
C2H−H2…O1XB	2.68	0.07
C6B−H6ax…O2XC	2.63	0.02
**C2D−H2…O4XC**	2.59	-0.02
C2E−H2…O4XC	2.69	0.08
**C6G−H6ax…O1XC**	2.59	-0.02
**C6H−H6ax…O1XC**	2.39	-0.22

**C6C−H6ax…O3XD**	2.44	-0.17
C6D−H6ax…O3XD	2.63	0.02
C2D−H2…O3XD	2.66	0.05
C2F−H2…O1XD	2.70	0.09
C2G−H2…O1XD	2.63	0.02
C6H−H6ax…O1XD	2.68	0.07

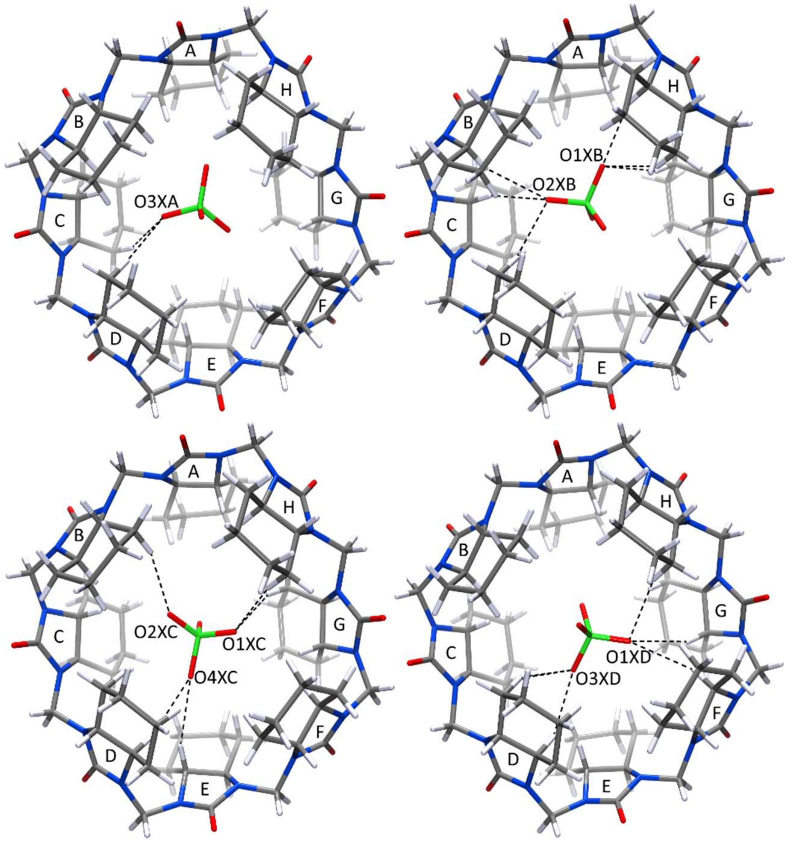

Figure S14. Close contacts within ClO_4@**cycHC[8]**, atoms involved in equivalent monomeric units are denoted with suffixes A-H. The contacts for the four disorder components are shown separately for clarity, but they occupy the same site with *sof* 0.291(8) for component XA (top left), *sof* 0.252(8) for component XB (top right), *sof* 0.219(8) for component XC (bottom left) and *sof* 0.237(8) for component XD (bottom right)

Tetrafluoroborate (BF_4^-)

Table S8. Shortest *d*(D-H···A) distances from the crystal structure of TBA(BF_4@**cycHC[8]**)· $2CH_3OH$, between host and BF_4^-. BF_4^- has four disorder components in the binding site, with *sof* 0.353(7)/0.162(7)/0.251(8)/0.234(8) for A/B/C/D respectively.

D−H···A	d(H···A), Å	D(H···A) − ∑r(*vdW*)[H, A], Å
**C6C−H6ax…F1A**	2.53	-0.03
C6D−H6ax…F1A	2.67	0.11
C2D−H2…F1A	2.62	0.06
C2G−H2…F4A	2.61	0.05
C6G−H6ax…F4A	2.68	0.12
**C6H−H6ax…F4A**	2.52	-0.04
**C6D−H6ax…F2B**	2.56	0.00

**C6G−H6ax…F4B**	2.39	-0.17
**C6H−H6ax…F4B**	2.55	-0.01
C2C−H2…F2C	2.66	0.10
**C6C−H6ax…F2C**	2.44	-0.12
C6H−H6ax…F4C	2.64	0.08
**C2H−H2…F4C**	2.47	-0.09
C2B−H2…F3D	2.66	0.10
**C2C−H2…F3D**	2.56	0.00
C6C−H6ax…F3D	2.68	0.12
C6D−H6ax…F3D	2.62	0.06
C6C−H6ax…F2D	2.60	0.04
C6G−H6ax…F1D	2.58	0.02
C2H−H2…F1D	2.68	0.12

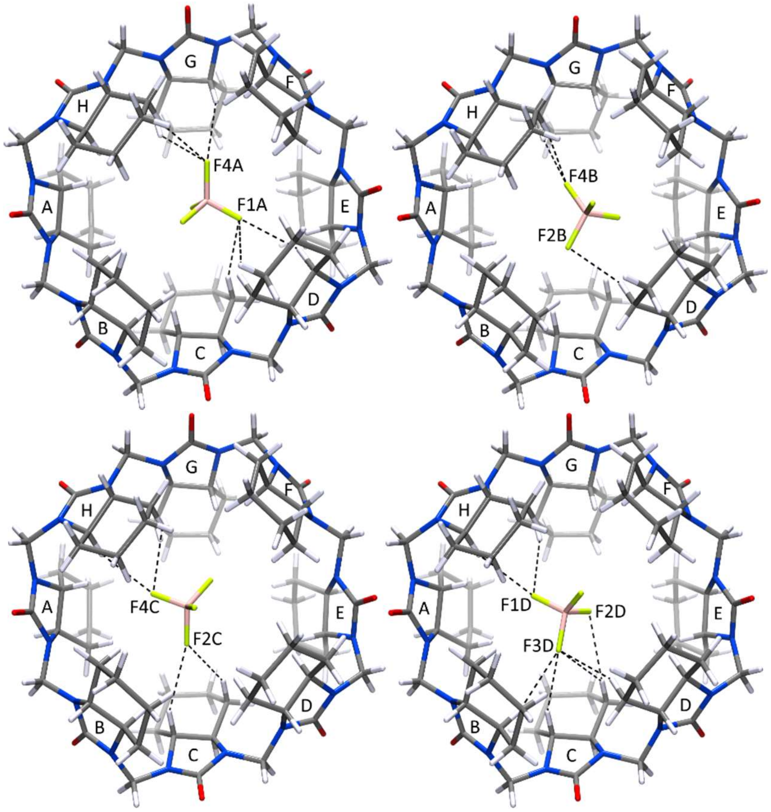

Figure S15. Close contacts within BF_4@**cycHC[8]**, atoms involved in equivalent monomeric units are denoted with suffixes A-H. The contacts for the four disorder components are shown separately for clarity, but they occupy the same site with *sof* 0.353(7) for component A (top left), *sof* 0.162(7) for component B (top right), 0.251(8) for component C (bottom left) and *sof* 0.234(8) for component D (bottom right)

Trifluoromethanesulfonate ($CF_3SO_3^-$)

Table S9. Shortest *d*(D-H···A) distances from the crystal structure of TBA(CF_3SO_3@**cycHC[8]**) · $2CH_3OH$, between the host and $CF_3SO_3^-$. The disorder component denoted with M has *sof* 0.124(3), disorder component I has *sof* 0.876(3)

D−H···A	**d(H···A), Å**	**D(H···A) − ∑r(*vdW*)[H, A], Å**
C6H−H6ax…O2M	2.65	0.04

**C2A−H2…O2M**	2.49	-0.12
**C2B−H2…O2M**	2.44	-0.17
**C2C−H2…O1M**	2.60	-0.01
**C2D−H2…O1M**	2.39	-0.22
C4D−H4ax…O3M	2.67	0.06
**C2E−H2…F1M**	2.42	-0.14
C6G−H6ax…F1M	2.65	0.09
C6G−H6ax…F2M	2.58	0.02
**C6G−H6ax…F3M**	2.48	-0.08
**C2G−H2…F3M**	2.32	-0.24
**C2H−H2…F3M**	2.56	0
**C2A−H2…O2N**	2.44	-0.17
**C2B−H2…O2N**	2.56	-0.05
**C2C−H2…O1N**	2.50	-0.11
**C2D−H2…O1N**	2.46	-0.15
C6D−H6ax…F1N	2.64	0.08
**C2E−H2…F1N**	2.56	0
**C2F−H2…F1N**	2.55	-0.01
C2G−H2…F3N	2.67	0.11
**C2H−H2…F3N**	2.41	-0.15

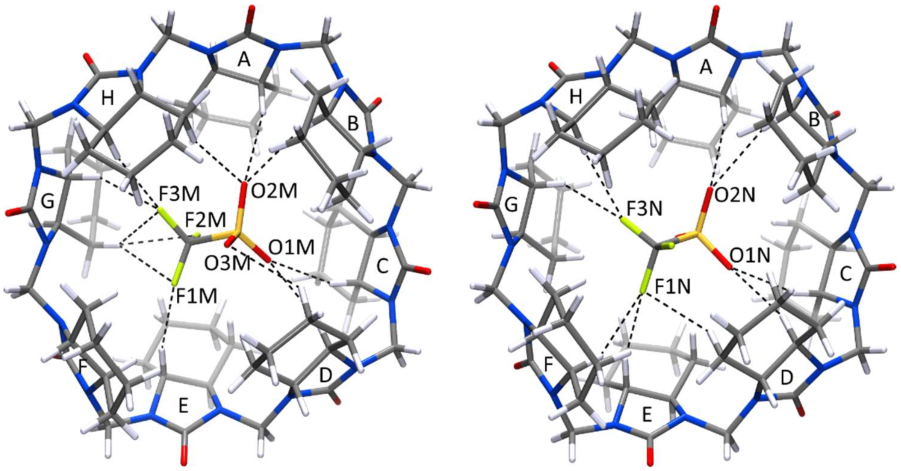

Figure S16. Close contacts within CF_3SO_3@**cycHC[8]**. Atoms involved in equivalent monomeric units of the host are denoted with suffixes A-H. The contacts for the two disorder components of $CF_3SO_3^-$ are shown separately for clarity, but they occupy the same site with *sof* 0.124(3) for component M (left) and *sof* 0.876(3) for component N (right)

3. Host-guest binding studies in solution

Determination of the stoichiometry of the cycHC[8] inclusion complexes

The stoichiometry of the anion complexes with **cycHC[8]** was determined by the method of continuous variation (MCV), also called the Job's method using ^{1}H NMR spectroscopy. The experiments were conducted for binary systems of the host [H] **cycHC[8]** and a chosen guest [G], where the total concentration of [H] + [G] was held constant ($3\cdot10^{-3}$ M or $4\cdot10^{-4}$ M), and the relative proportions of [H] and [G] were varied from [H]/[G] = 1 to [H]/[G] = 0. The ^{1}H NMR data for the Job plots was measured for a selection of guests ($TBAPF_6$, $TBAClO_4$, $TBABF_4$ or $NaSbF_6$) in deuterated methanol at room temperature using a Bruker Avance III 400MHz spectrometer. The Job plots were drawn by following the chemical shift of the **cycHC[8]** proton signals while varying the [H]/[G] ratio. Chemical shift of the host proton 2*ax* was followed, with the exception of $NaSbF_6$, where due to the broadening of proton 2*ax* signal, proton 1*ax* was followed. The maximum shift change was seen at α = 0.5 in all of the Job plot curves, indicating a 1:1 molecular association (Figure S17).

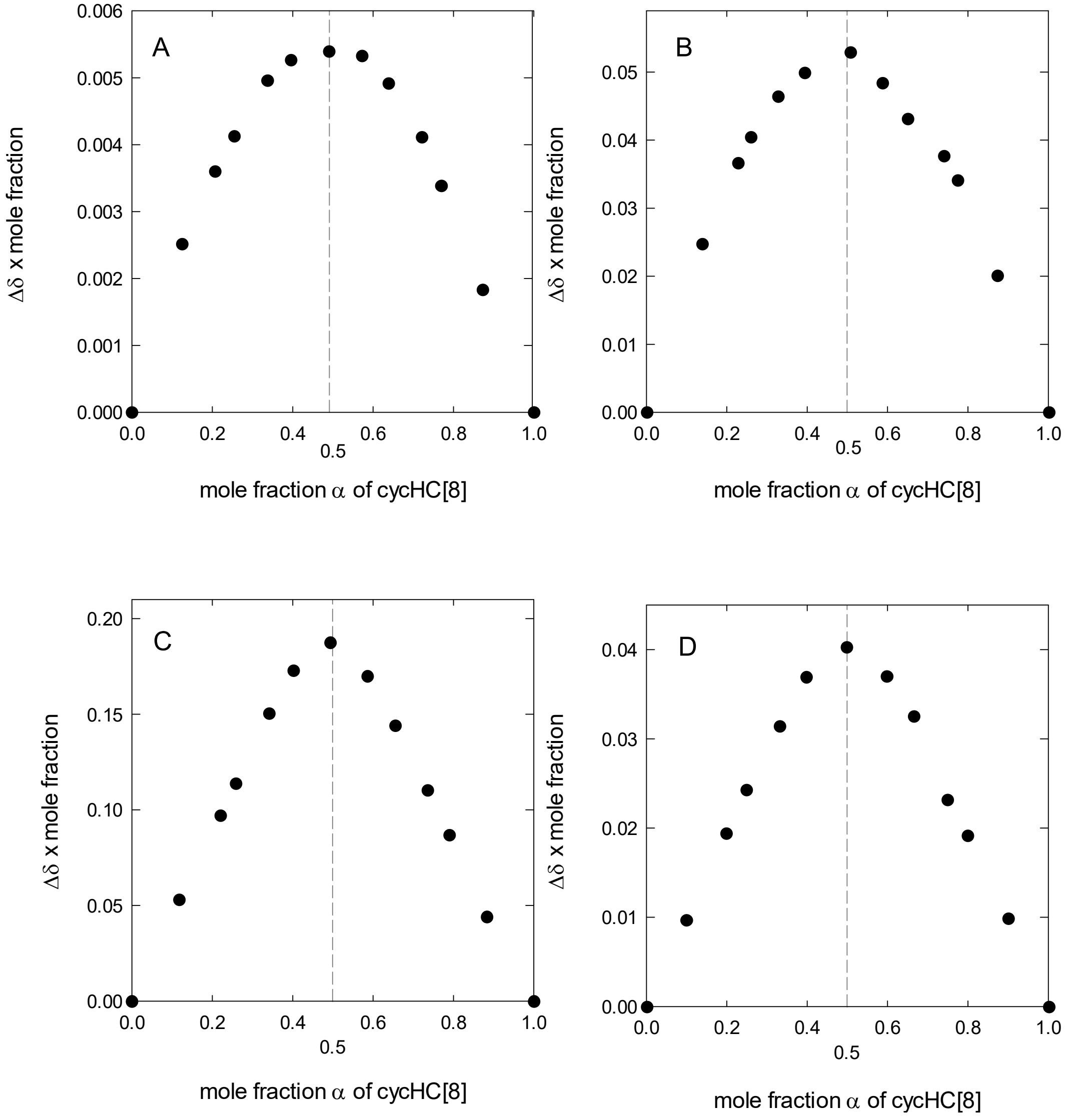

Figure S17. Job plots for cycHC[8] with: A) $TBABF_4$; B) $TBAClO_4$; C) $TBAPF_6$ and D) $NaSbF_6$

Determination of the association constants

General remarks

Guest affinity was ascertained by ^{1}H NMR titration experiments in deuterated methanol at 288 K. The concentration of **cycHC[8]** for a titration experiment was chosen according to the predicted guest binding strength (host concentrations 4mM, 3mM, 0.4mM or 0.04mM were used) and held constant throughout the titration sequence. The guest stock solution was added to the titration samples in small aliquots cumulatively and the spectra recorded upon each addition. The concentration of guest stock solution was chosen so that the total volume of added guest stock solution would not affect the overall concentration of the components in the sample more than 10%. The ^{1}H NMR spectra were recorded on a Bruker Avance III 800MHz spectrometer using regular 5 mm NMR tubes. Probe temperature was set to 288 K and each sample was allowed to thermally equilibrate in the probe for at least 10 min prior to measurements. The progressive changes of the chemical shifts of 1*ax*, 2*ax* and 3*eq* protons were followed as small aliquots of guest stock solution were added to a solution containing **cycHC[8]**, resulting in a set of spectra (18-21 per titration) for each of the tested guests. Precise host-guest ratio in the titration sequence was determined by integration of the ^{1}H signal for the host 1*ax* proton (2.91-2.82ppm) and either the methyl signal (1.03 ppm) of guest counter cation TBA or the added internal standard 1,3,5-tris(trifluoromethyl)benzene (8.3515 ppm). The relaxation time for the signals used for quantification was measured on concentrations relevant to the titration experiment setup, using the t1ur pulse programme. This gave relaxation times for: **cycHC[8]** proton 1*ax* 647(8) ms (4mM), 582(5) ms (0.4 mM), 509(7) ms (0.04 mM); TBA CH_3 group 1.25(2) s (40 mM), 1.40(2) s (4 mM), 1.40(1) s (0.4 mM) and the internal standard aromatic proton 4.7 s. Therefore the spectra in the titration sequences with TBA salts were measured with pulse duration of 5 μs and relaxation delay of 1 s, whereas titrations using an internal standard were performed with pulse duration of 5 μs and relaxation delay of 5 s, according to the longer relaxation time (T_1 = 4.7 s) measured for the internal standard protons.

Chemical shifts were referenced to the residual methanol signal at 3.310 ppm. Additional peaks on low concentration titrations (0.04 mM) arise from solvent impurities (plastificators), and as no change in their chemical shifts is seen throughout the titration experiment, they are considered not to affect the complexation. The titration data was fitted by nonlinear regression analysis to a 1:1 binding isotherm as the Job plot analysis and crystallographic evidence suggested a 1:1 complexation model. The equation used for nonlinear curve fitting of the resulting binding data (equation 1) was adapted from what was used by Nitschke et al.[12]

$$f = y0 + Dy\frac{(K_a(P+x)+1) - \sqrt{(K_a(P+x)+1)^2 - 4K_a{}^2Px}}{2K_aP}$$

Where *f* is the measured chemical shift, *y0* is the chemical shift of the empty **cycHC[8]**, *Dy* is the difference of the chemical shift of an empty host and a fully occupied host, K_a is the association constant, *P* is the total host concentration and *x* is the total guest concentration

Following three signals instead of only one afforded a reliable estimation of the association constant, since the data is less biased by the possible errors on reading the chemical shifts, especially since the migrating signals were seen to overlap at certain guest ratios in the titration sequences.

Stacked spectra of the titration experiments and fitted isotherms of the titration data

$NaSbF_6$

600µL of 0.04mM **cycHC[8]** stock solution was used for each of the 5 titration samples. To achieve a concentration range of added guest (0-10 equivalent [G] to [H]), a 4.8mM $NaSbF_6$ stock solution was prepared and added to titration samples cumulatively in small aliquots. To be able to determine the precise host-guest ratio in a titration sequence, 1,3,5-tris(trifluoromethyl)benzene was added in 1:1 ratio to the guest stock solution. The precise host-guest ratio in a titration sequence was determined by integration of the ^{1}H signals for host 1*ax* (2.92-2.82ppm) or 3*eq* (2.56-2.45ppm) and the signal of the added standard in the aromatic region (8.3515ppm).

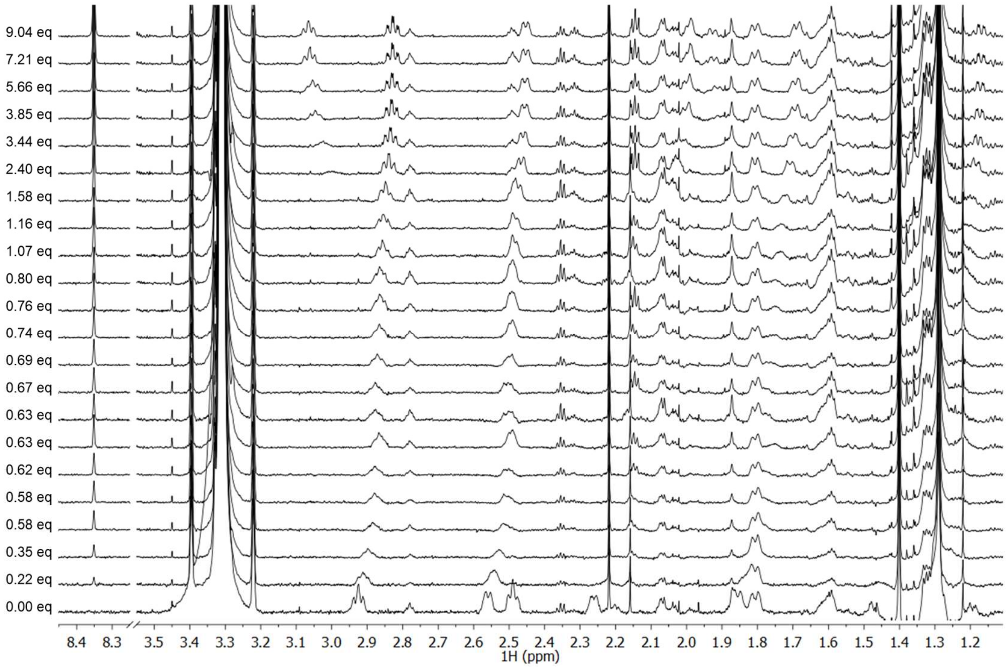

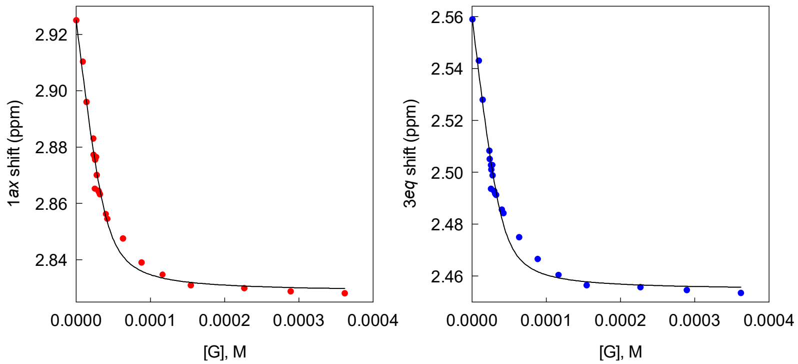

	K_a	Std. Error	R^2
1*ax*	234750	43195	0.9832
3*eq*	266327	54267	0.9814
$K_{a\,(avg)}$	249881	69455	
K_a		**2.5±0.7 · 10^5**	

$TBAPF_6$

600µL of 0.4mM **cycHC[8]** stock solution was used for each of the 5 titration samples. To achieve a concentration range of added guest (0-10 equivalent [G] to [H]), a 48mM $TBAPF_6$ stock solution was prepared and added to titration samples cumulatively. The precise host-guest ratio in a titration sequence was determined by integration of the ^{1}H signal for host 1*ax* proton (2.91-2.82ppm) and the methyl group (1.03ppm) of guest counter cation TBA.

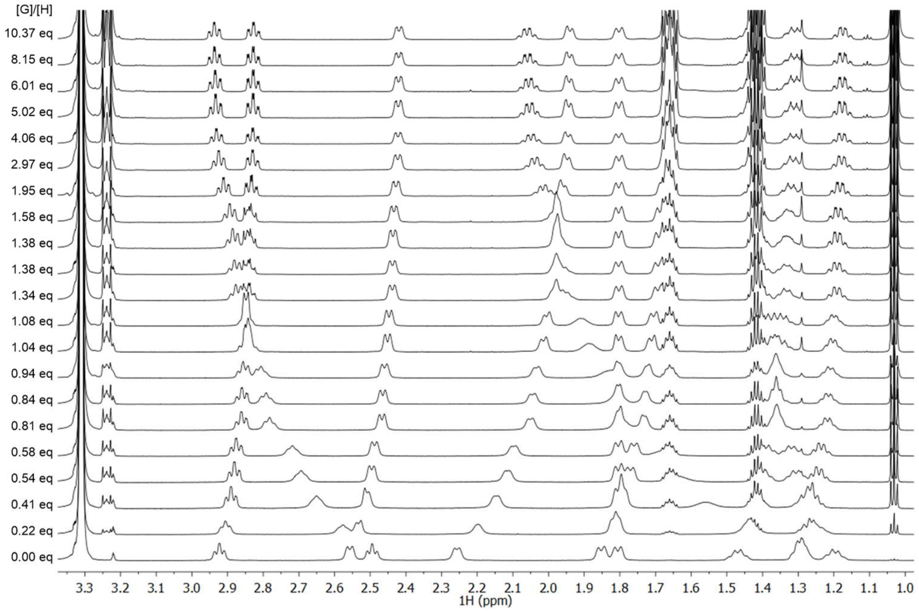

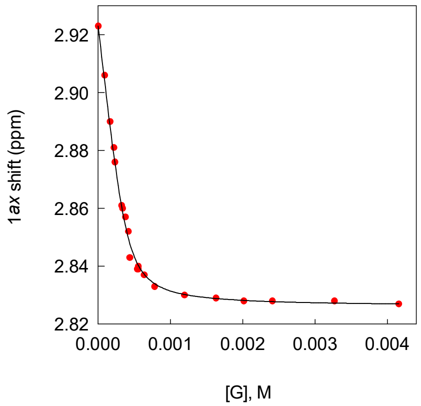

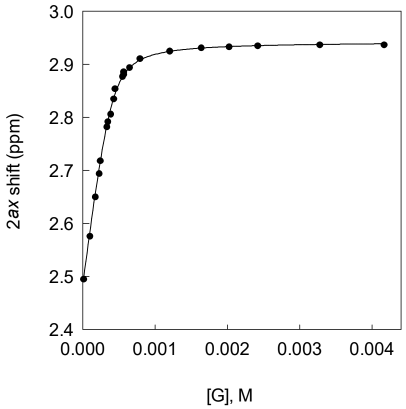

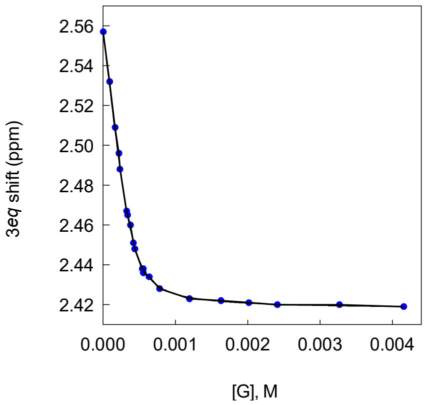

	K_a	Std. err.	R^2
1*ax*	27391	2702	0.9959
2*ax*	28793	1706	0.9985
3*eq*	27768	1447	0.9988
$K_{a\ (avg)}$	27984	3508	
K_a	**$2.8 \pm 0.4 \cdot 10^4$**		

TBAPF$_6$ (1:1 D_2O/MeOD)

600µL of 0.04mM **cycHC[8]** stock solution was used for each of the 18 titration samples. To achieve a concentration range of added guest (0-5 equivalent [G] to [H]), a 4.8mM TBAPF$_6$ stock solution was prepared and added to the titration samples in appropriate volume (1-25µL). The precise host-guest ratio in a titration sequence was determined by integration of the ^{1}H signal for host 1*ax* proton (2.91-2.82ppm) and the methyl group (1.03ppm) of guest counter cation TBA.

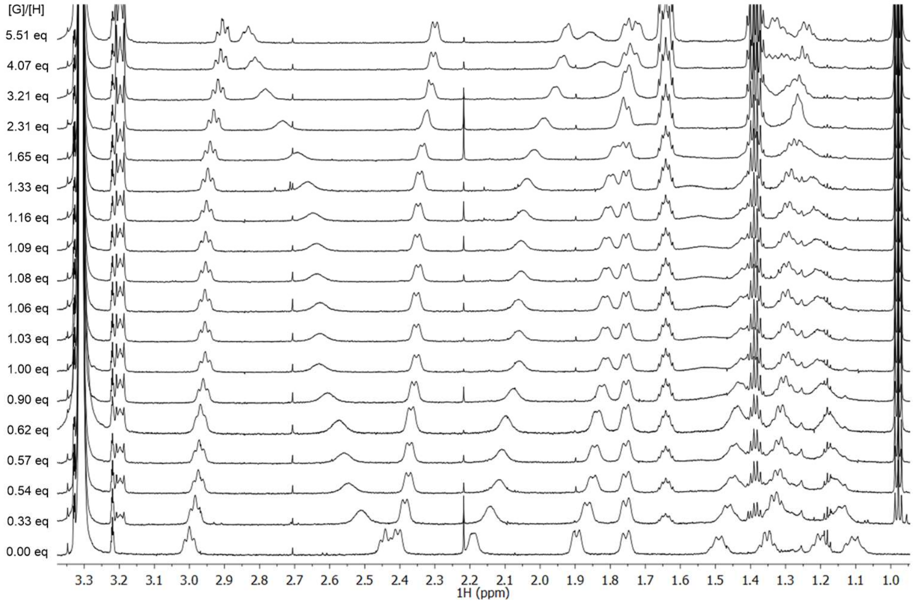

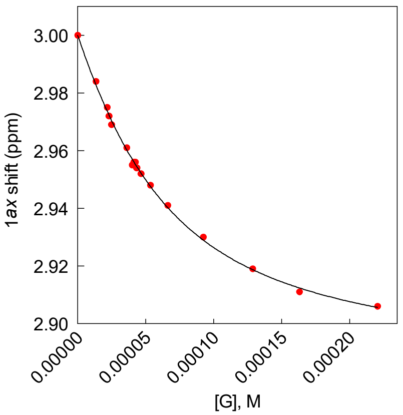

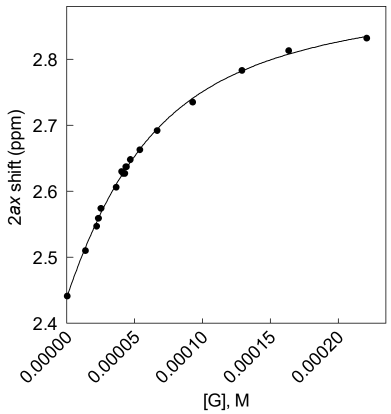

	K_a	Std. err.	R^2
1*ax*	23404	911	0.9985
2*ax*	25215	1019	0.9984
3*eq*	30310	1625	0.9972
$K_{a\,(avg)}$	26310	2124	

K_a	**$2.6 \pm 0.2 \cdot 10^4$**

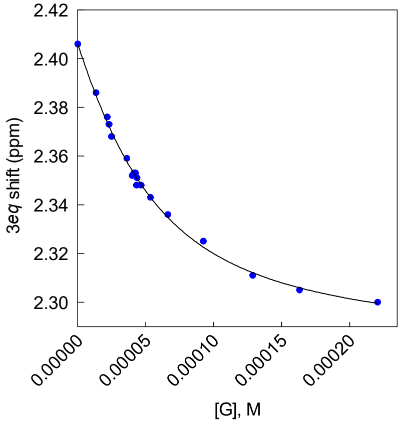

$NaPF_6$

600µL of 0.4mM **cycHC[8]** stock solution was used for each of the 5 titration samples. To achieve a concentration range of added guest (0-10 equivalent [G] to [H]), a 48mM $NaPF_6$ stock solution was prepared and added to titration samples cumulatively. To be able to determine the precise host-guest ratio in the titration sequence, 1,3,5-tris(trifluoromethyl)benzene was added in 1:1 ratio to the guest stock solution. The precise host-guest ratio in a titration sequence was determined by integration of the ^{1}H signals for host 1*ax* (2.92-2.82ppm) proton signals and the signal of the added standard (8.3515ppm).

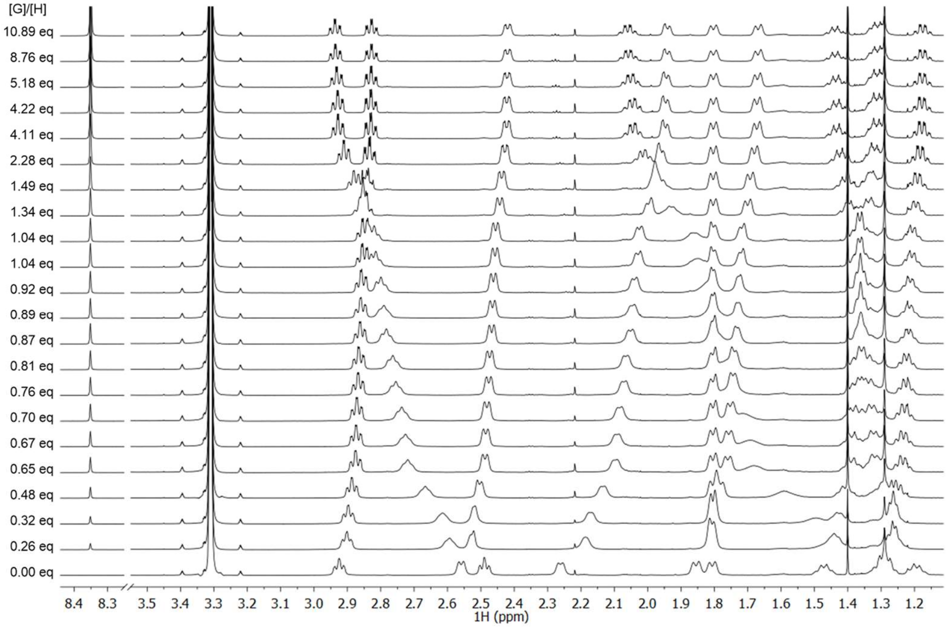

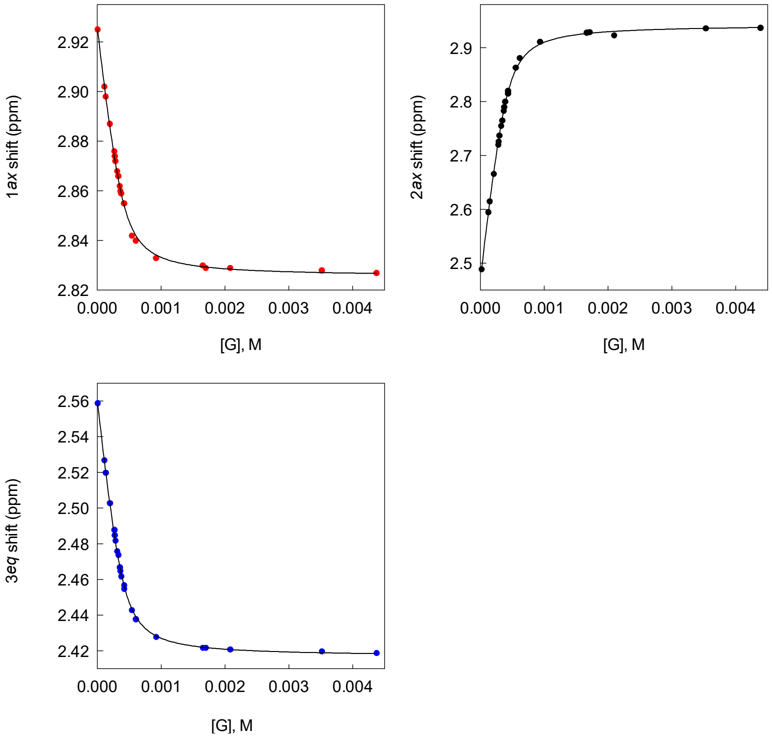

	K_a	Std. err.	R^2
1*ax*	18869	1012	0.9980
2*ax*	21186	876	0.9989
3*eq*	20534	768	0.9991
$K_{a\,(avg)}$	20197	1543	
K_a	**$2.0 \pm 0.2 \cdot 10^4$**		

The pulse program of this measurement was not suitable for quantitative evaluation of the standard, 1,3,5-tris(trifluoromethyl)benzene (at 8.3515 ppm) signal intensity, as the signal was suppressed due to insufficient relaxation delay between scans (ideal P1=30 and D1= 5s, used pulse program P1=90 and D1=1s). The standard signals were therefore reassessed using a calibration curve obtained from measuring a number of the titration samples of **cycHC[8]**+$NaPF_6$+Std at each of the pulse programs P1=90, D1=1s and P1=30, D1=5s and evaluating the increase of the standard signal relative to the corresponding 1*ax* signal of the **cycHC[8]**. Added guest concentration, used in the curve-fitting of the binding isotherm, was calculated based on the corrected integral values for the standard,

Calibration data:

		**The relative integral of Std compared to the 1*ax* signal of the cycHC[8]**	
		Initial pulse program (P1=90, D1=1s)	Adjusted pulse program (P1=30, D1=5s)
**Sample no**	[G], M	Integral	Integral
**151020155**	0.00024	0.14	0.23
**151020168**	0.0012	0.85	1.61
**151020169**	0.00164	0.87	1.63
**151020170**	0.002	1.10	2.03
**151020171**	0.0032	1.81	3.23
**151020172**	0.004	2.25	4.07

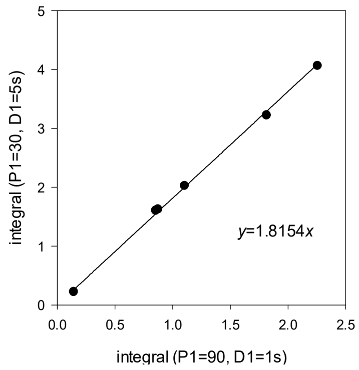

TBAReO$_4$

600µL of 0.4mM **cycHC[8]** stock solution was used for each of the 5 titration samples. To achieve a concentration range of added guest (0-10 equivalent [G] to [H]), a 48mM TBAReO$_4$ stock solution was prepared and added to titration samples cumulatively. The precise host-guest ratio in a titration sequence was determined by integration of the ^{1}H signal for host 1*ax* proton (2.91-2.82ppm) and the methyl group (1.03ppm) of guest counter cation TBA.

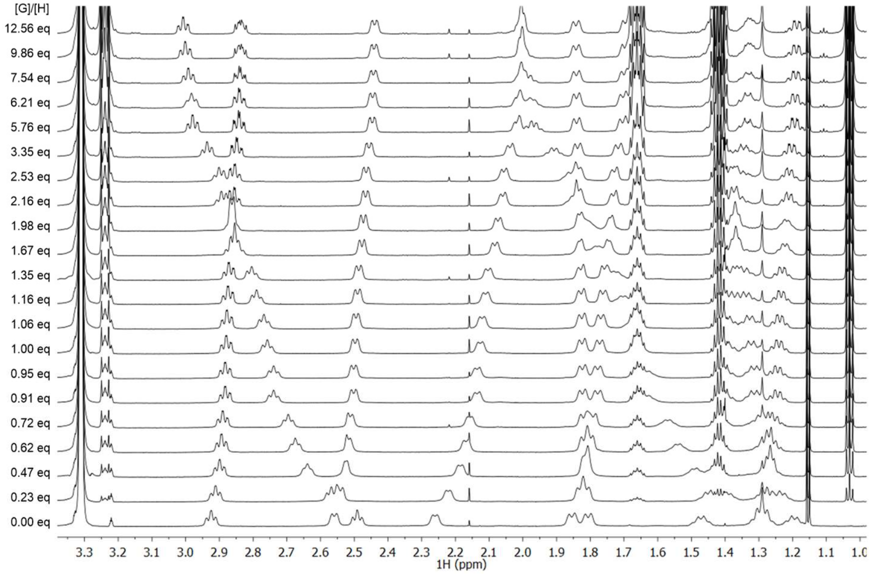

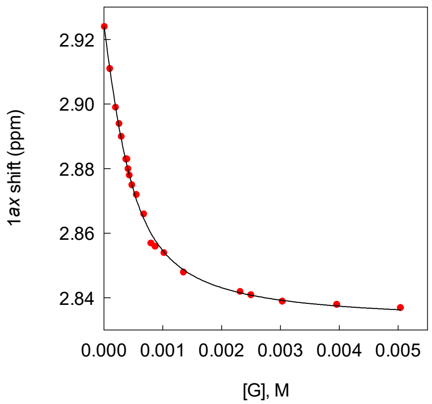

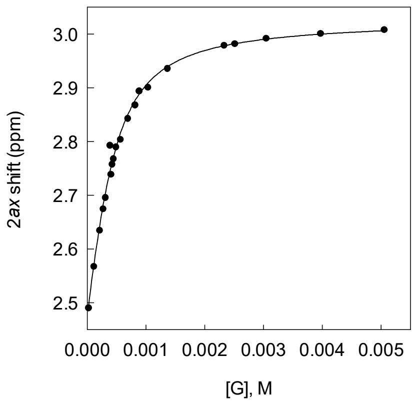

	K_a	Std. err.	R^2
1*ax*	4451	158	0.9982
2*ax*	4987	358	0.9929
3*eq*	4702	119	0.9991
$K_{a\,(avg)}$	4713	409	

K_a	**4.7±0.4 · 10^3**

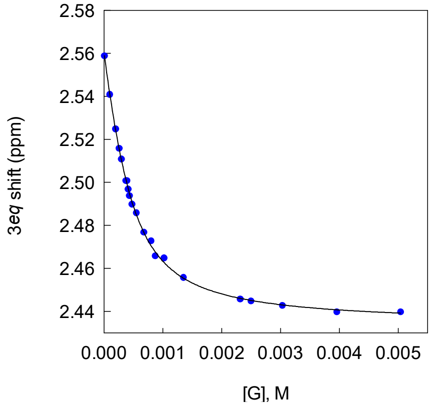

$NaIO_4$

600µL of 0.4mM **cycHC[8]** stock solution was used for each of the 5 titration samples. To achieve a concentration range of added guest (0-10 equivalent [G] to [H]), a stock solution of 48mM $NaIO_4$ was prepared and added to titration samples cumulatively. To be able to determine the precise host-guest ratio in the titration sequence, 1,3,5-tris(trifluoromethyl)benzene was added in 1:1 ratio to the guest stock solution. The precise host-guest ratio in a titration sequence was determined by integration of the ^1H signals for host 1*ax* (2.92-2.82ppm) proton signals and the signal of the added standard in the aromatic region (8.35 ppm).

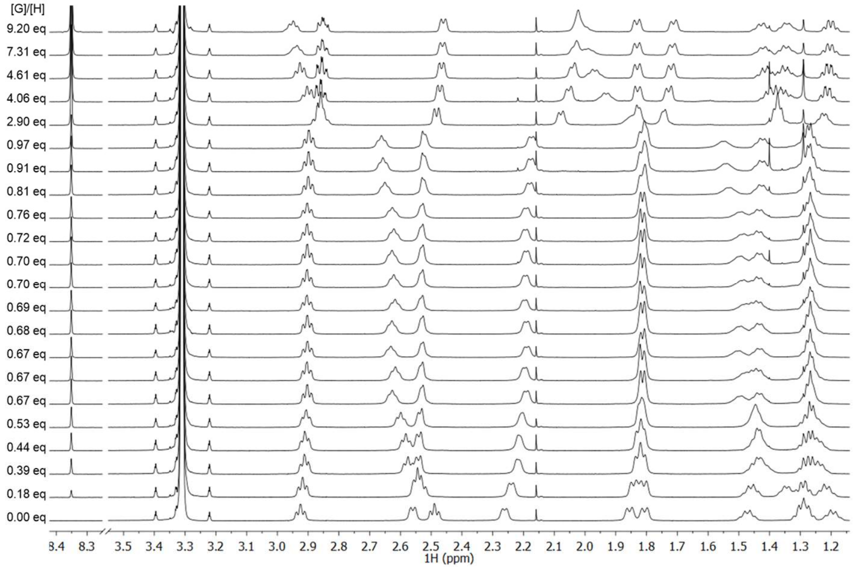

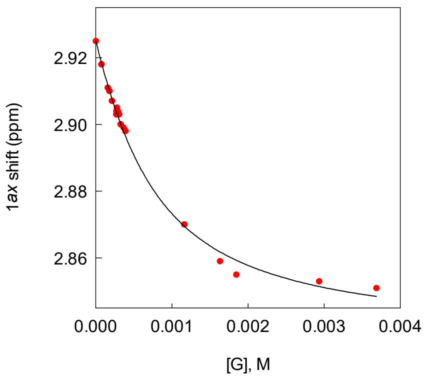

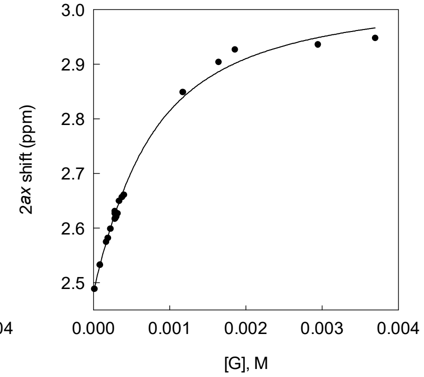

	K_a	Std. err.	R^2
1*ax*	1804	113	0.9950
2*ax*	1905	126	0.9944
3*eq*	1889	109	0.9957
$K_{a\ (avg)}$	1866	201	
K_a		**1.8±0.2 · 10³**	

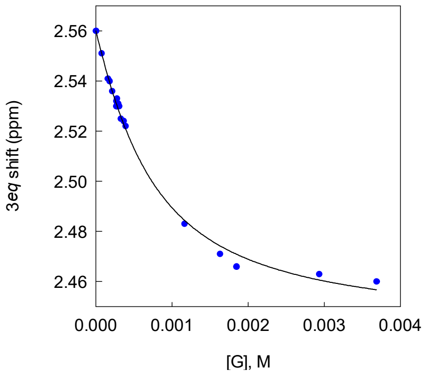

TBAClO$_4$

600µL of 0.4mM **cycHC[8]** stock solution was used for each of the 3 titration samples. To achieve a concentration range of added guest (0-10 equivalent [G] to [H]), a 48mM TBAClO$_4$ stock solution was prepared and added to titration samples cumulatively. The precise host-guest ratio in a titration sequence was determined by integration of the ^{1}H signal for host 1*ax* proton (2.91-2.82ppm) and the methyl group (1.03ppm) of guest counter cation TBA.

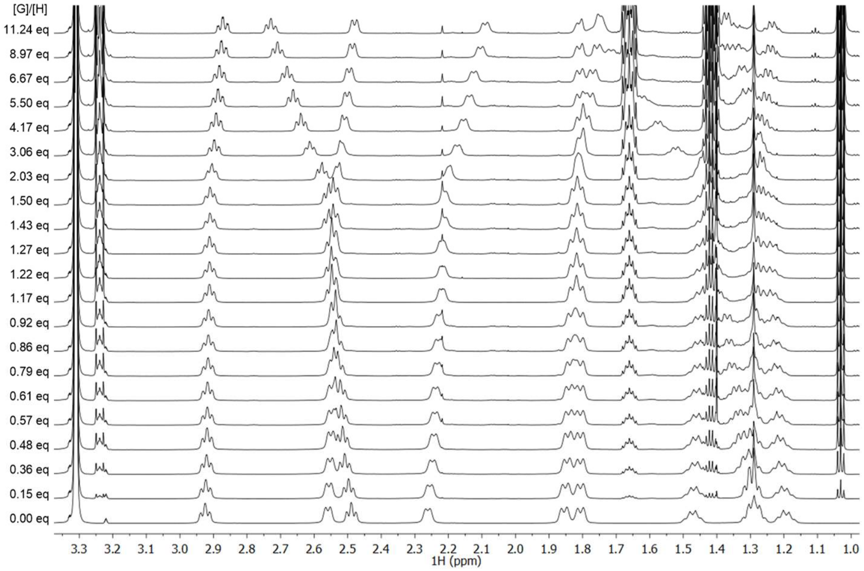

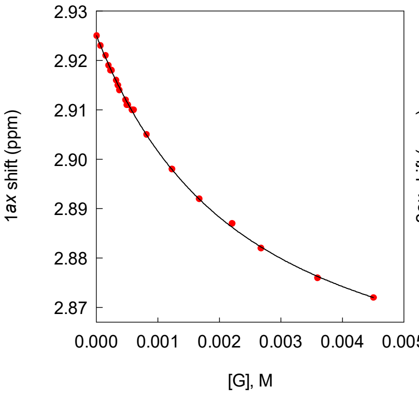

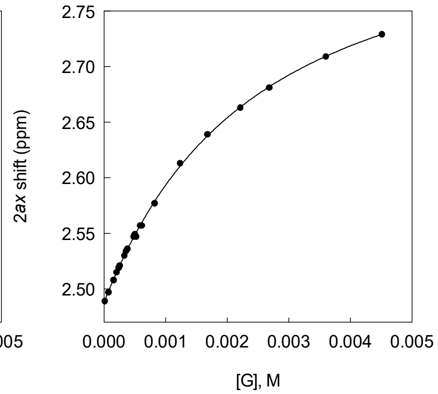

	K_a	Std. err.	R^2
1*ax*	478	12	0.9994
2*ax*	461	9	0.9997
3*eq*	467	17	0.9989
$K_{a\ (avg)}$	469	23	
K_a		**4.7±0.2 · 10^2**	

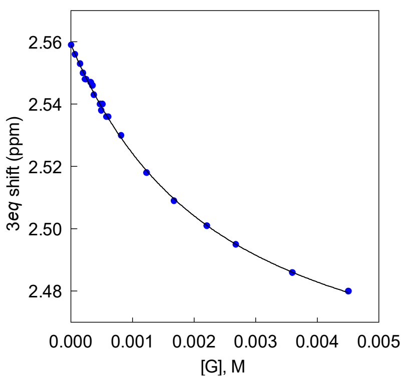

$TBABF_4$

600µL of 3mM **cycHC[8]** stock solution was used for each of the 5 titration samples. To achieve a concentration range of added guest (0-10 equivalent [G] to [H]), a 0.36M (360mM) $TBABF_4$ stock solution was prepared and added to titration samples cumulatively. The precise host-guest ratio in a titration sequence was determined by integration of the ^{1}H signal for host 1*ax* proton (2.91-2.82ppm) and the methyl group (1.03ppm) of guest counter cation TBA.

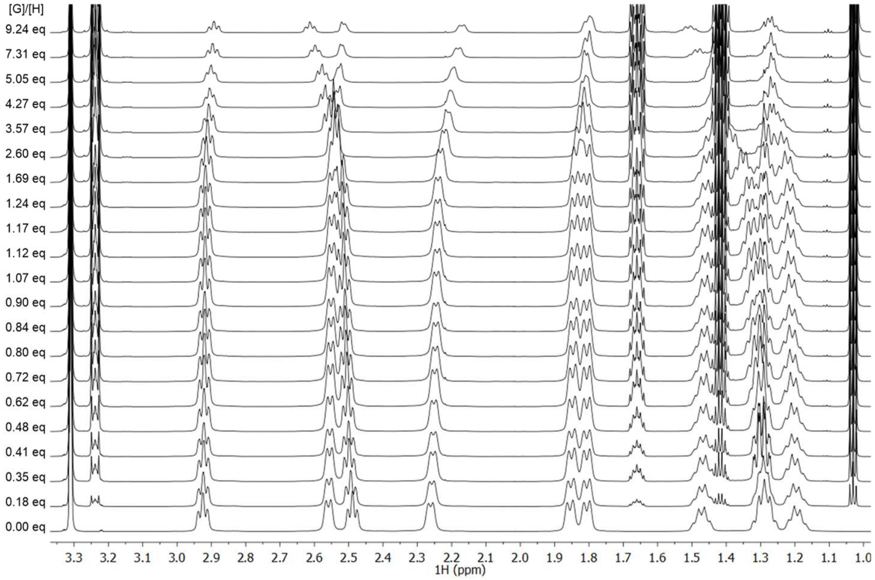

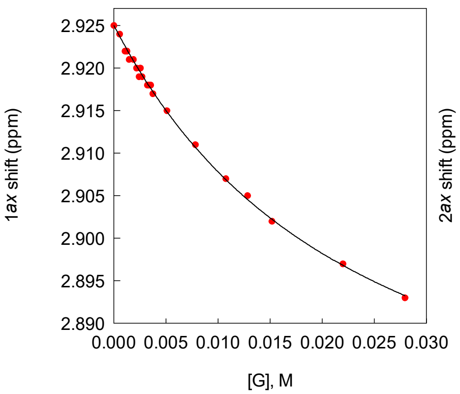

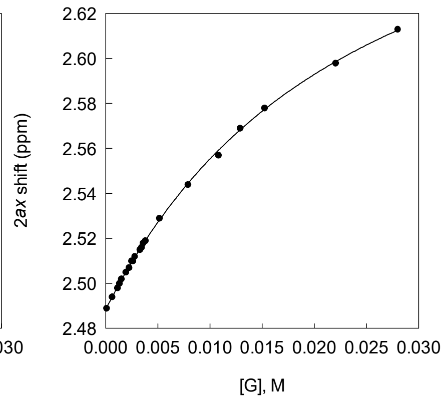

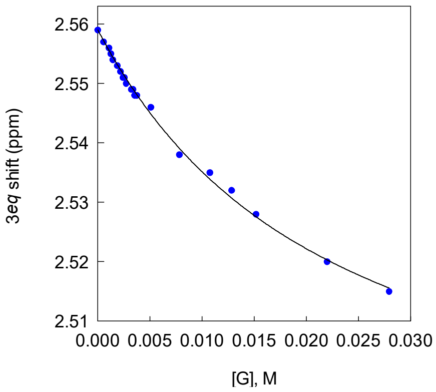

	K_a	Std. err.	R^2
1*ax*	47	2	0.9984
2*ax*	46	1	0.9996
3*eq*	50	3	0.9972
$K_{a\,(avg)}$	48	4	
K_a	**$4.8 \pm 0.4 \cdot 10^1$**		

$TBACF_3SO_3$

600µL of 3mM **cycHC[8]** stock solution was used for each of the 5 titration samples. To achieve a concentration range of added guest (0-10 equivalent [G] to [H]), a 0.36M (360mM) $TBACF_3SO_3$ stock solution was prepared and added to titration samples cumulatively. The precise host-guest ratio in a titration sequence was determined by integration of the ^{1}H signal for host 1*ax* proton (2.91-2.82ppm) and the methyl group (1.03ppm) of guest counter cation TBA.

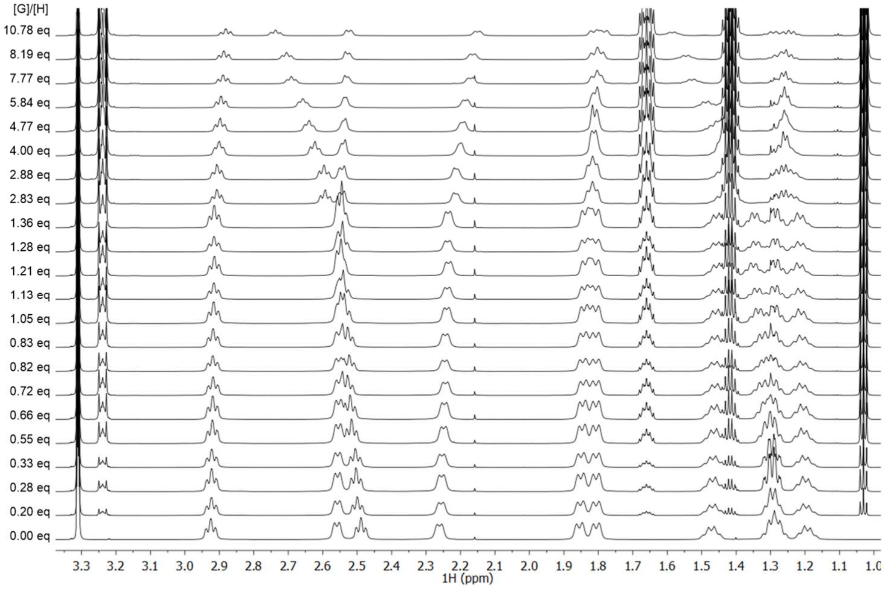

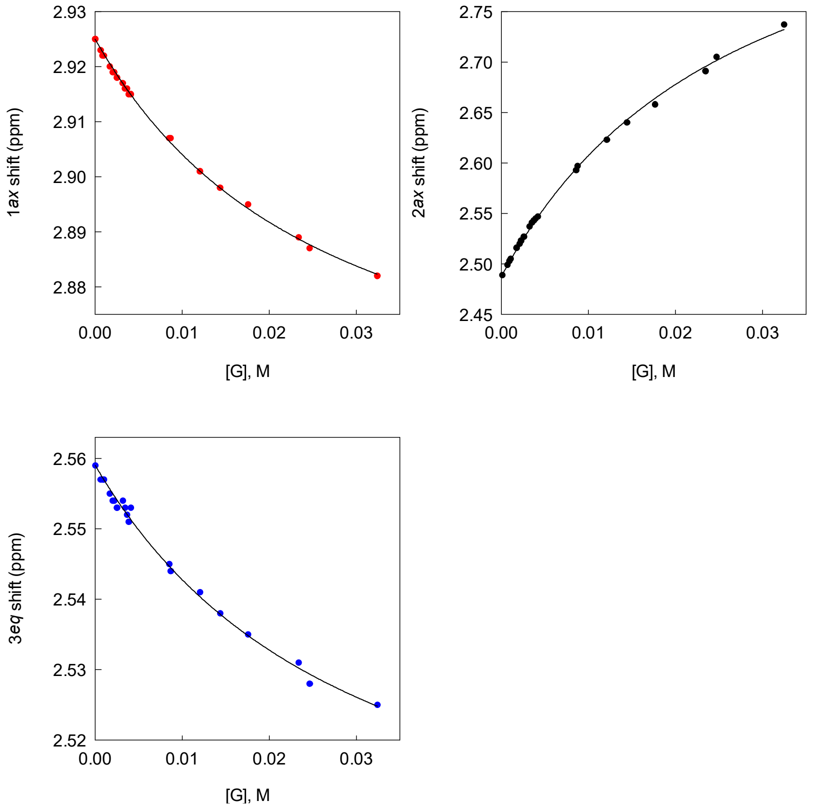

	K_a	Std. err.	R^2
1*ax*	41	2	0.9985
2*ax*	41	2	0.9987
3*eq*	36	4	0.9940
$K_{a\,(avg)}$	39	5	
K_a		**$3.9 \pm 0.5 \cdot 10^1$**	

$TBACF_3CO_2$

$TBACF_3CO_2$ was prepared ahead of titration from trifluoroacetic acid ant tetrabutylammonium hydroxide. 600µL of 3mM **cycHC[8]** stock solution was used for each of 5 titration samples. A 0.36M (360mM) $TBACF_3CO_2$ stock solution was prepared and added to titration samples cumulatively. Precise determination of host-guest ratio in a titration sequence was determined by integration of the ^{1}H signal for host 1*ax* proton (2.91-2.82ppm) and the methyl group (1.03ppm) of guest counter cation TBA. Spectra with up to 15.23 eq of $TBACF_3CO_2$ were collected, and from these it was assessed that K_a must be lower than 10. Determination of the precise association constant was not conducted, as the overall shift was so small even in the case where large excess of $TBACF_3CO_2$ was added.

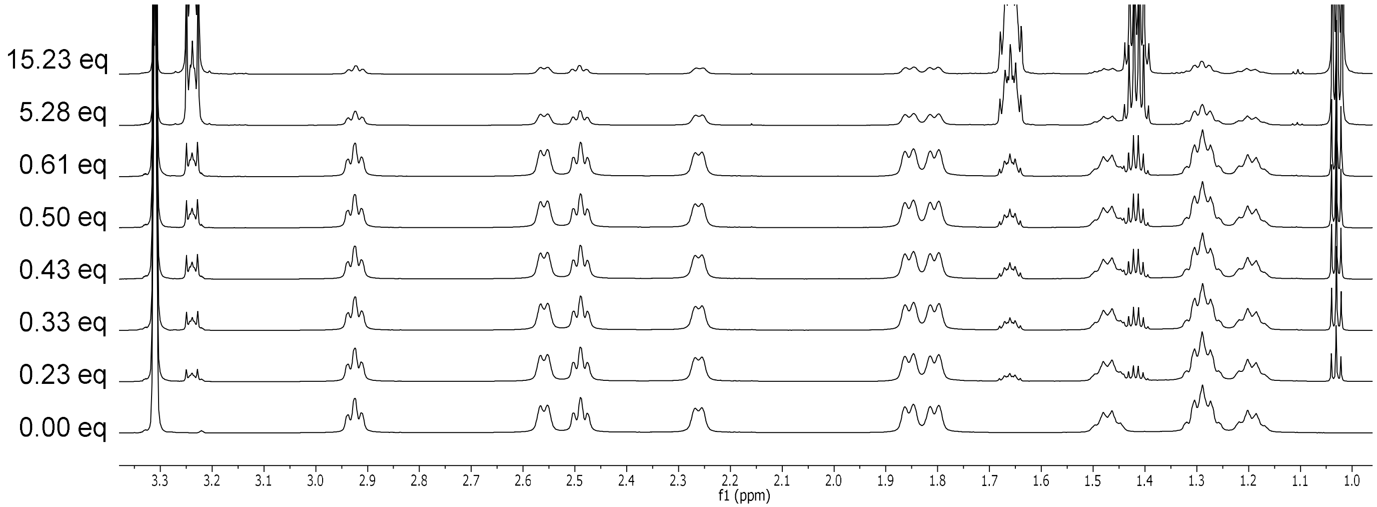

ITC measurements

Calorimetric measurements were performed on a MicroCal iTC200 calorimeter (GE Healthcare Life Sciences). The volume of the calorimetric cell was 200 µl and the size of the syringe was 40 µl. All experiments were carried out in methanol. In the case of the interaction between $NaSbF_6$ and **cycHC[8]**, calorimetric cell was loaded with 0.2 mM $NaSbF_6$ solution and the syringe was filled with 2.4 mM **cycHC[8]** solution. Sequential injections (1 µl) of **cycHC[8]** were added at 60 s intervals by rotating syringe to the calorimetric cell. Since the heat of dilution was negligible, it was not subtracted from the total heat of the interaction. Such a protocol was chosen as the heat of dilution of **cycHC[8]** was negligible compared to the heat of dilution observed using the protocol of sequential addition of a concentrated $NaSbF_6$ solution to the calorimetric cell containing a **cycHC[8]** solution.

Using the same protocol and solution concentrations was attempted for studying the interactions of $NaPF_6$ with **cycHC[8]**, but was deemed not optimal for obtaining a well-fitting binding isotherm due to the lower binding strength of PF_6^-. Simple increasing of the solution concentrations while using the same protocol was not applicable as a solution of 24mM of **cycHC[8]** is outside the solubility limits of **cycHC[8]** in methanol. Thus in order to investigate the interaction between $NaPF_6$ and **cycHC[8]**, higher solution concentrations were achieved by loading the calorimetric cell with 2.5 mM **cycHC[8]** solution and filling the syringe with 31.39 mM $NaPF_6$. Sequential injections (1 µl) of $NaPF_6$ were added at 60 s intervals by rotating syringe to the calorimetric cell. The heat of dilution was obtained by injecting $NaPF_6$ to methanol. Prior to data analysis the heat of dilution was subtracted from the corresponding total heat of the interaction.

All measurements were carried out at 298 K and the stirrer speed was set to 600 rpm.

ORIGIN 7.0 with MicroCal AddOn was used for data analysis. Data were fitted using simple one-site model.

	n	K_a (M^{-1})	ΔH (kJ mol^{-1})	ΔS (J mol^{-1} K^{-1})
$NaSbF_6$	0.861	$(1.02\pm0.03)\cdot10^5$	−56.2±0.3	−92.8
$NaPF_6$	0.818	$(1.29\pm0.04)\cdot10^4$	−43.8±0.2	−68.3

The deviation from n = 1 could be caused by small uncertainties of the used solutions concentrations. In NMR spectroscopic methods the concentration of the solutions can be verified by an internal standard, whereas the concentrations of the solutions used in the ITC measurements rely on the preparation of the stock solutions. The uncertainties in concentration would reflect in the stoichiometry parameter, which is allowed to vary in the fitting of the binding isotherm. The resulting n = 0.82-0.86 is nevertheless close enough to n = 1, and 1:1 stoichiometry of binding is also supported by ^{1}H-NMR Job plot analysis in methanol, X-ray crystallography in solid state and MS in gas phase.

A) **cycHC[8]** to $NaSbF_6$

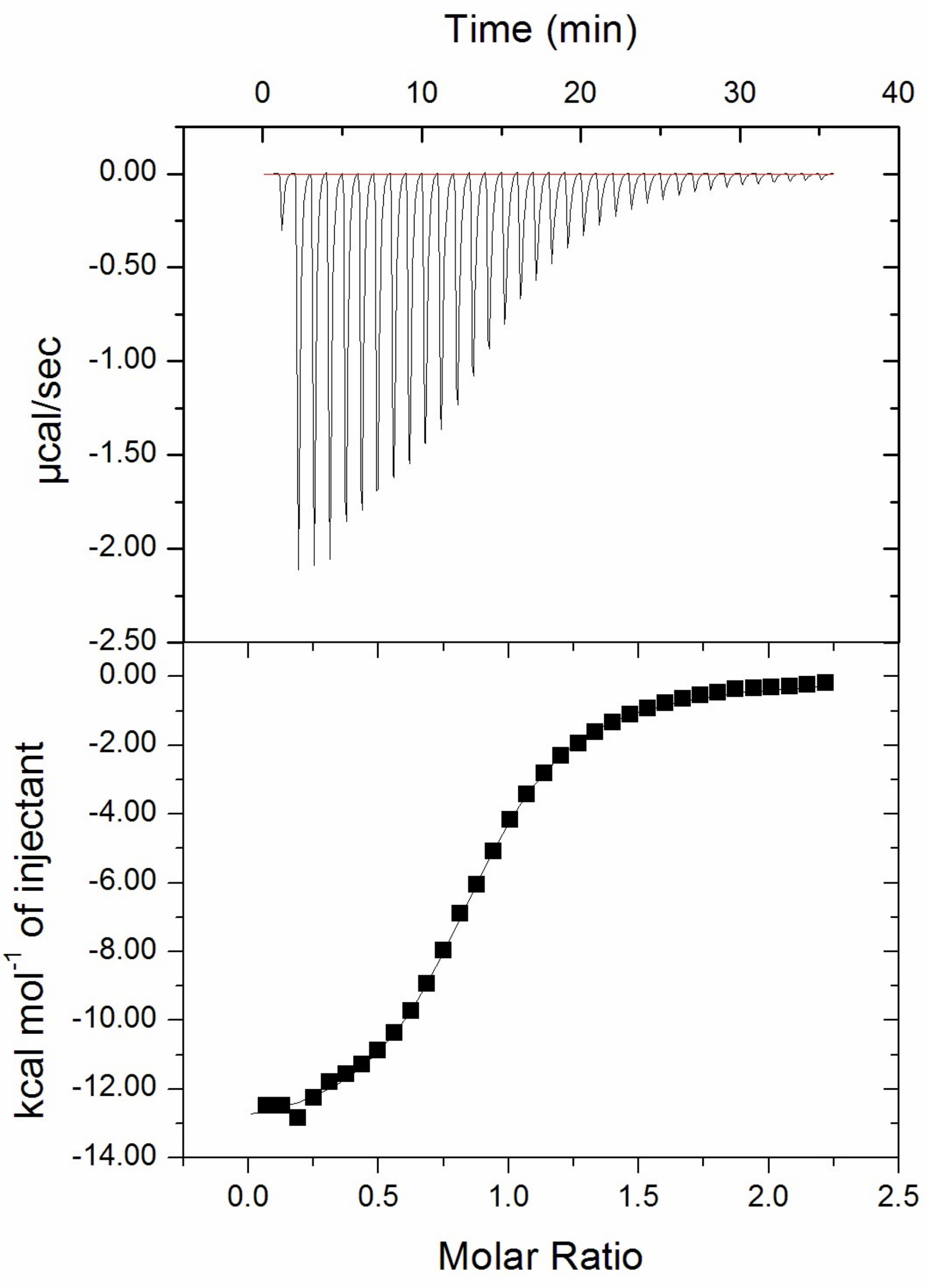

B) $NaPF_6$ to **cycHC[8]**

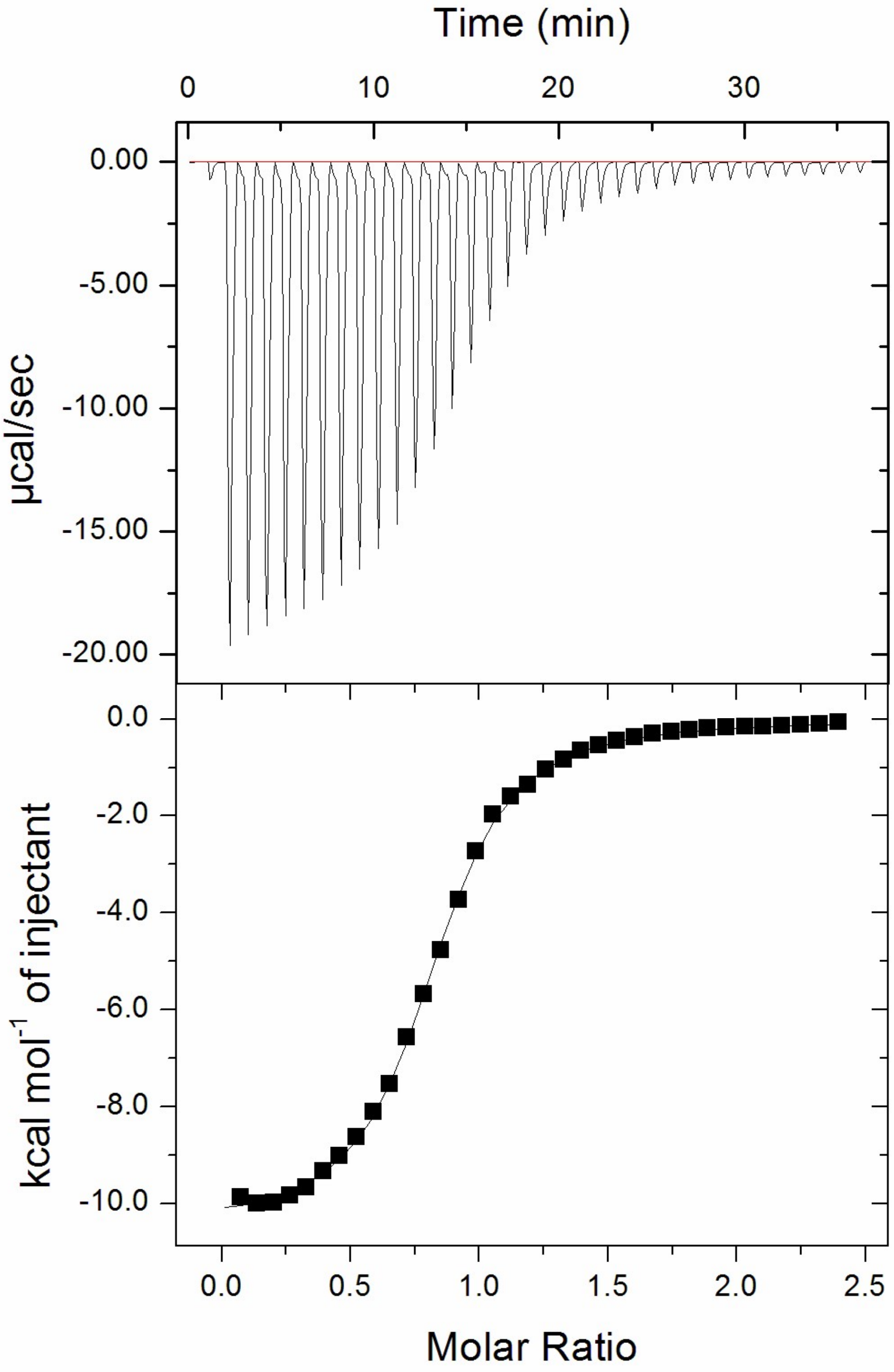

Variable temperature NMR for NaSbF6 and NaPF6 inclusion complexes

General methods

The $2ax$ and $3eq$ proton resonances for $NaPF_6$ (1.5 mM) and $3eq$ proton resonances for $NaSbF_6$ (1.5 mM) in the variable temperature NMR spectra of a methanol-d_4 solution of **cycHC[8]** (2.6 mM) in the temperature region of 204–295 K in 4 K temperature increments were simulated with the program WINDNMR V. 7.1.14 (http://www.chem.wisc.edu/areas/reich/plt/windnmr.htm**)** after processing the NMR spectra with NUTS. The coalescence temperatures were 241 K for $NaPF_6$ and 253 K for $NaSbF_6$, respectively. The $3eq$ and $2ax$ resonances for $NaPF_6$ and the $3eq$ resonance for $NaSbF_6$ were present as free and complexed **cycHC[8]** in the slow exchange region at low temperatures and were therefore suitable for simulations. DNMR option with 2dddd was used in WINDNMR to simulate spectra with all input parameters except temperature taken from the slow-exchange or near slow-exchange limit spectra. Adjustments of spectral amplitudes and rate constants (k_{assoc} + k_{dissoc}) were carried out iteratively until the line-shapes matched. The coalescence region was neglected for determining activation parameters due to large broadening of the peaks and difficulty in obtaining good fits. The simulated spectra are shown in shown in Figure S20 and Figure S21 and fitting data are listed for $NaSbF_6$ in Table S10 and for $NaPF_6$ in Table S11. Temperatures were calibrated using an external standard MeOH solution.

NMR titration experiments and ITC data indicate that the equilibrium greatly favors the host-guest complex. Furthermore, it was not possible to detect either the free host or a precomplex with PF_6^- at 0.4 mM concentration and in 1.5 times excess of the guest at sub-coalescence temperature of 213 K. This also suggests that the equilibrium constant for the overall reaction is greatly in favor of the host-guest complex. Accordingly, the dissociation rate constant is negligible compared to the association rate constant. Therefore, the observed sum of rate constants, obtained from lineshape analysis in WINDNMR, is approximately equal to the rate constant of the association reaction. Based on the assumption that $k_{asssoc} + k_{dissoc} = k_{assoc}$, the calculated activation parameters, obtained from the Eyring analysis, are shown in Table 2 in the main text and the Eyring plots brought in Figure S 19. Experimental details to confirm a first-order association reaction are given below.

Activation parameters for both the dissociation and association reactions were obtained from modified Eyring equation:

$$\ln\left(\frac{k}{\mathrm{T}}\right) = -\Delta\mathrm{H}^{\ddagger}\frac{1}{\mathrm{RT}} + \left(23.76 + \Delta\mathrm{S}^{\ddagger}\frac{1}{\mathrm{R}}\right)$$

via a linear plot ln(k/T) versus 1/T.

The activation Gibbs free energy, enthalpy and entropy are detailed in Table 2 in the main text.

Association-dissociation reaction order

The dissociation reaction is unambiguously a first-order process (H – host; G – guest, HG - complex):

$$\mathrm{HG} \longrightarrow \mathrm{H} + \mathrm{G}$$

The dissociation reaction rate is given by the equation:

$$r = k_{dissoc} \times [HG]$$

We have determined that the association reaction is also a first-order process. Based on the reaction coordinate derived from DFT calculations (Section 5, SI) and the dilution experiments that are brought below, complexation reaction proceeds through a low energy intermediate precomplex $(HG)_1$:

$$\mathrm{H} + \mathrm{G} \rightleftharpoons (\mathrm{HG})_1 \rightleftharpoons (\mathrm{HG})_2$$

This low-energy pathway is visualized in Figure S18 B.

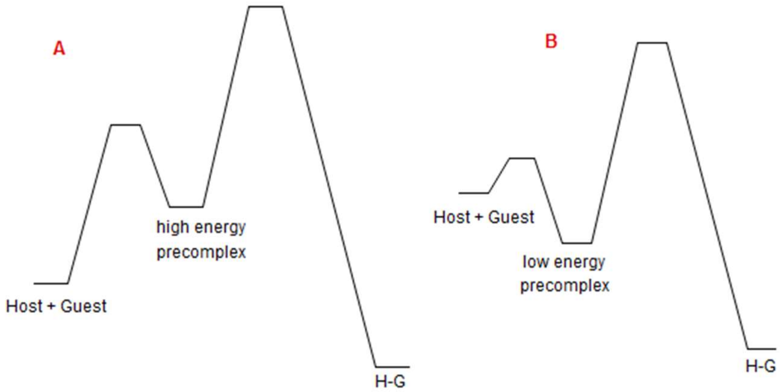

Figure S18. (A) showing host-guest complex formation via a high energy precomplex, which results in a second-order association reaction, and (B) showing host-guest complex formation via a low energy precomplex, which results in presaturation of the precomplex and a first-order association reaction

In order to find the association reaction rate order, we performed a series of dilution experiments (Figure S19) near the coalescence temperature of $NaPF_6$. The peak shapes and hence the rate of the reaction did not change on diluting the sample, which implies presaturation and the low energy precomplex formation according to Figure S18 B and therefore also a first-order association reaction.

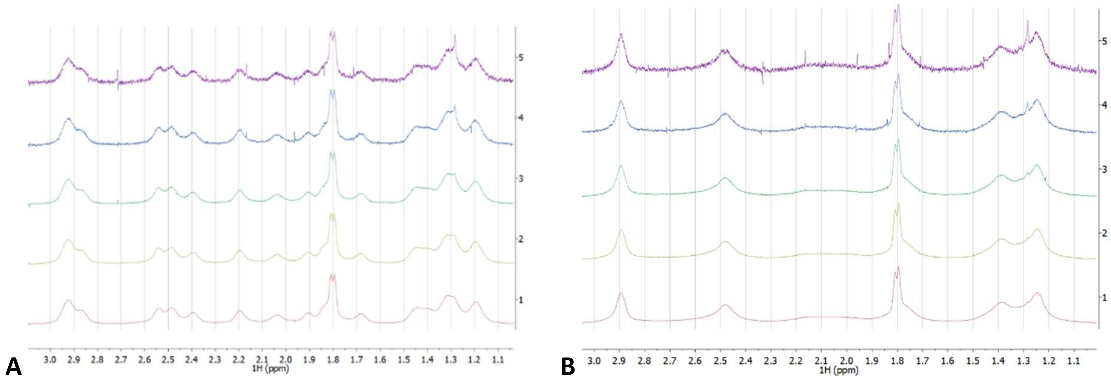

Figure S19. (A) Dilution experiments of 2:1 ratio of H:G at 233 K; (B) at 249 K. The concentrations started with 3 mM host and 1.5 mM guest (1st stacked spectrum) and they were diluted two times up to 0.1875 mM host and 0.09375 mM guest (5th stacked spectrum)

WINDNMR fits

The 2*ax* proton signals for $NaPF_6$ were chosen for simulations as those protons have good lineshapes over a large range of temperatures and-are separated from other protons with at least one of the peaks uninterrupted by other peaks at all temperatures. For $NaSbF_6$, the 3*eq* proton was observable over the whole temperature range, whereas 2*ax* proton was significantly broadened at higher temperatures.

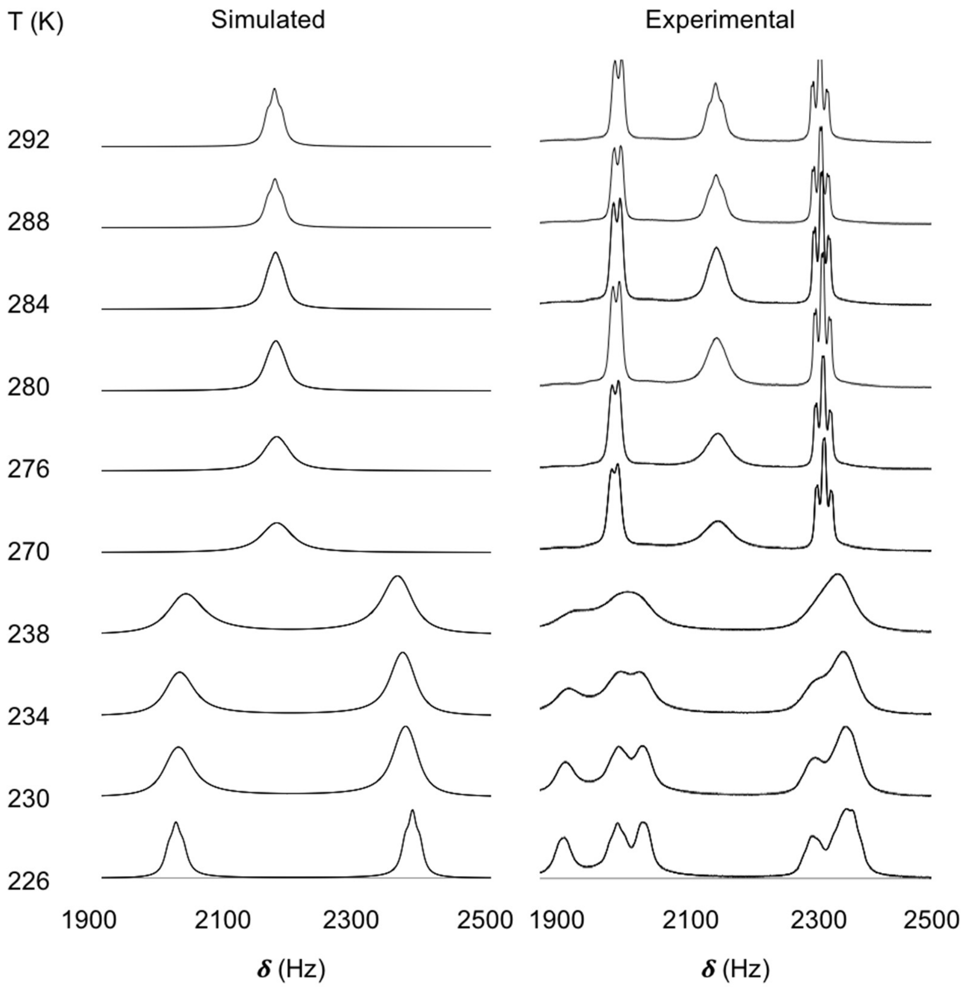

Figure S20. Simulated and experimental spectra of the variable temperature study as obtained from WinDNMR lineshape analysis for $NaPF_6$.

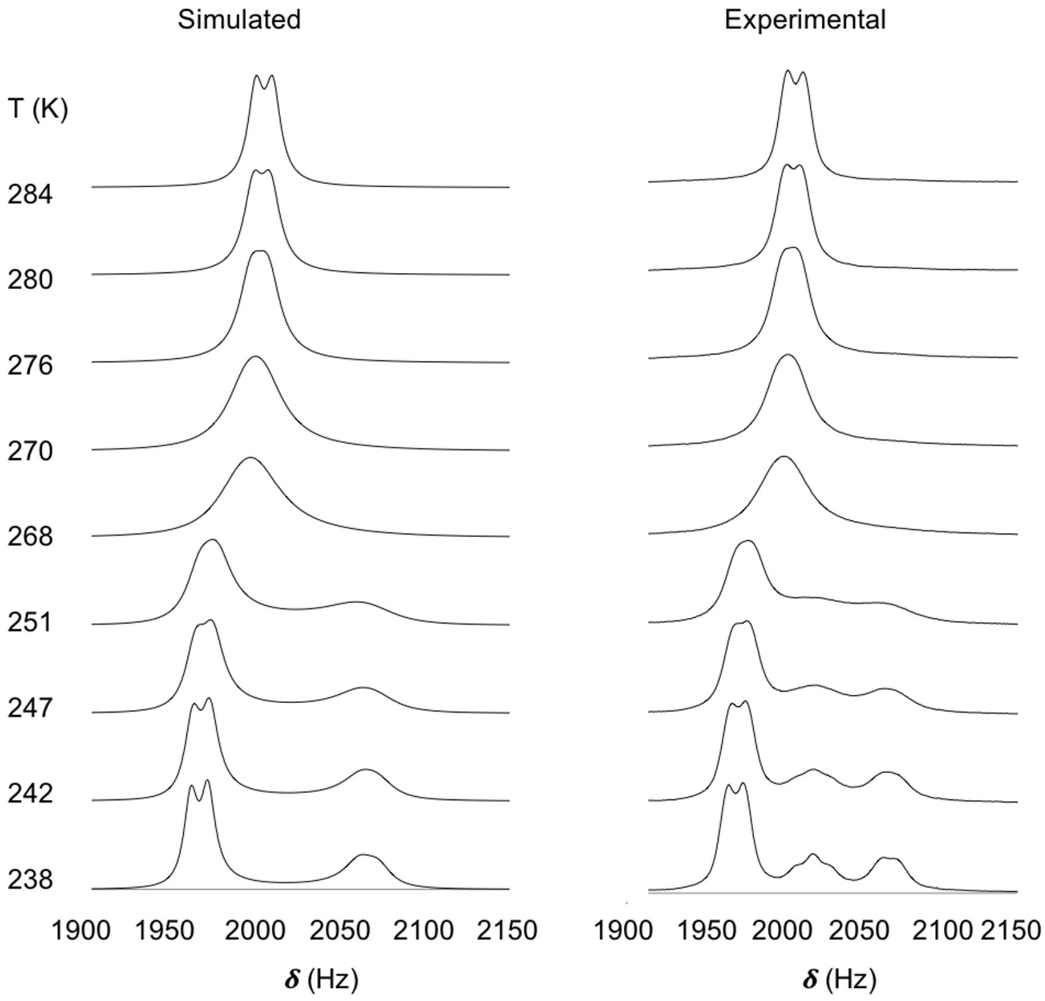

Figure S21. Simulated and experimental spectra of the variable temperature study as obtained from WinDNMR lineshape analysis for $NaSbF_6$.

Table S10. Temperature, sum of host-guest complex formation reaction rate constants, individual association reaction rate constants and natural logarithms used for the Eyring equation for 3*eq* proton for $NaSbF_6$. Coalescence region was not used for obtaining fits.

T (K)	k_{assoc} +k_{dissoc} (s^{-1})	k_{assoc} (s^{-1})	ln(k_{assoc}/T)
238	90	90	-0,97
242	109	109	-0,79
247	146	146	-0,53
251	197	197	-0,24
268	696	696	0,96

270	877	877	1,18
276	1496	1496	1,69
280	1835	1835	1,88
284	2216	2216	2,05

Table S11. Temperature, sum of host-guest complex formation reaction rate constants, individual association reaction rate constants and natural logarithms used for the Eyring equation for 2*ax* proton for $NaPF_6$. Coalescence region was not used for obtaining fits.

T (K)	$k_{assoc} + k_{dissoc}$ (s^{-1})	k_{assoc} (s^{-1})	$\ln(k_{assoc}/T)$
226	93	93	-0.89
230	153	153	-0.41
234	222	222	-0.05
238	308	308	0.26
270	5209	5209	2.96
276	6801	6801	3.20
280	9901	9901	3.56
284	12301	12301	3.77
288	16001	16001	4.02
292	17501	17501	4.09

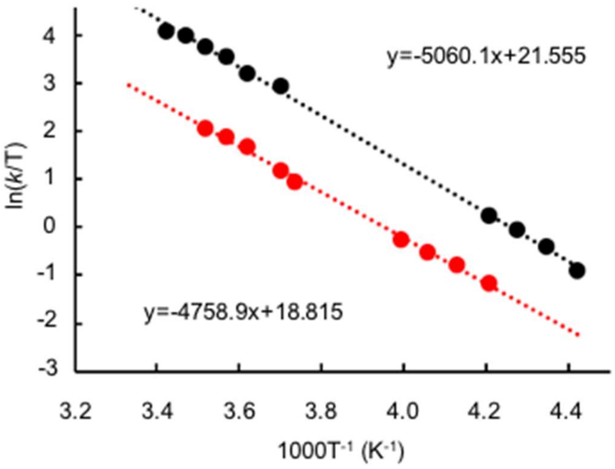

Figure S22. Eyring plot for the association process for SbF_6^- (red circles) and PF_6^- (black circles).

4. Computational details

Because the guest molecules are not chiral the calculated complexation energies do not depend on the chirality of the host, the geometry of (*all*-*S*)-cyclohexanohemicucurbit[8]uril was chosen from the previous computational work of Prigorchenko et al.[1] Geometry optimization for local minima were carried out using the BP86[14] functional, along with the def2-SV(P)[15] basis set. Energies were refined using the BP86-D functional, along with the def2-TZVPDref[15] basis set. The heavier atoms were described with the inclusion of the appropriate Stuttgart pseudopotential.[16] Vibrational analysis calculations were performed to ensure all chosen geometries were at local minima. The solvation model COSMO[17] (methanol ε = 32.613) was used for calculations. The total energies were calculated using the Gibbs free energy correction from the vibrational analysis. Gibbs free energy was estimated using the temperature 293.15 K and the pressure 0.1 MPa (if not stated otherwise). The calculations were performed using the program package Turbomole 6.5[18]. The maps of electrostatic potential (MEPs) were generated using Gaussian 09[19]. COSMO was not used for generating MEPs. The van der Waals radii for Sb (2.06), I (1.98) and Re (2.00) were added manually.[20] The results do not include a basis set superposition error correction due to the incompatibility between the continuum solvation model (COSMO) and the counterpoise approach to BSSE correction.

The formation of PF_6^-@cycHC[8] complex in MeOH

Number of solvent molecules in the cavity of cycHC[8]

Encapsulated water molecules are higher in energy compared to the bulk water molecules.[21] This energy difference, in turn, affects the binding energy of the guest (high-energy solvent is released, guest-host complex forms, thus the total energy of the system is lower). The energy difference between the encapsulated and free solvent is governed by the cavity size (larger cavities allow more stable H-bonded networks) and the amount of molecules in it. Thus the number of the solvent molecules inside the cavity and the structure of the encapsulated solvent molecules play an important role in studying the formation of a host-guest complex.

According to the crystal structure of **cycHC[8]**, the number of solvent molecules (methanol) captured in the cavity of **cycHC[8]** varies from one to three.[1] Our previous research[22] indicates that the nitrogen atoms are the strongest hydrogen bond acceptors of the **cycHC[8]**, thus the captured methanol forms a hydrogen bond with **cycHC[8]** through its hydrogen atom as depicted on Figure S23 a.

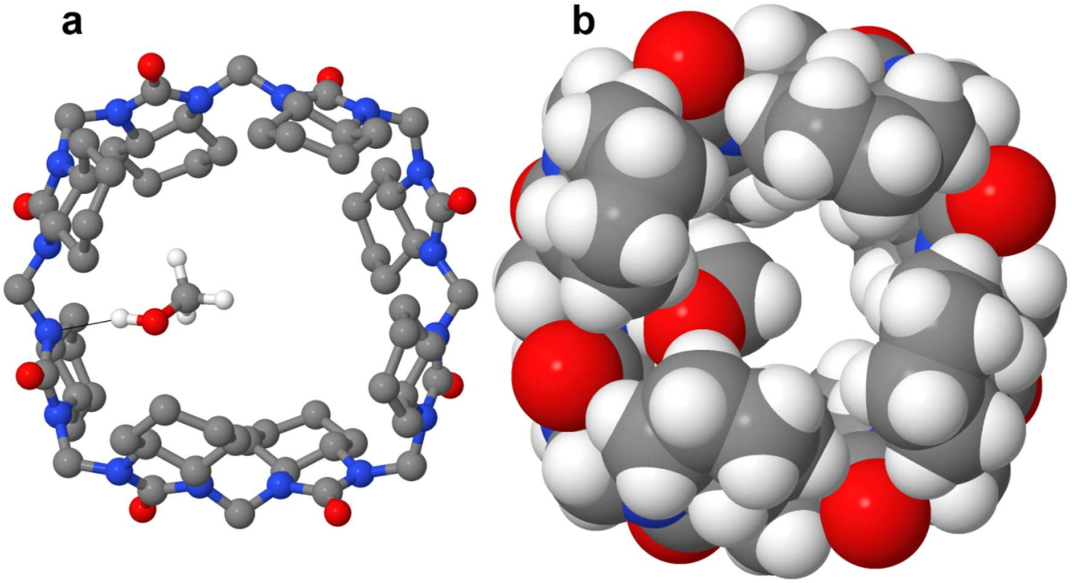

Figure S23. (a) MeOH@**cycHC[8]** complex, where **cycHC[8]** is visualized without hydrogen atoms and hydrogen bond as black line; (b) MeOH@**cycHC[8]** complex visualized with van der Waals radii.

A search for binding positions of second methanol was conducted and eight positions of two methanol molecules were considered as depicted on Figure S24.

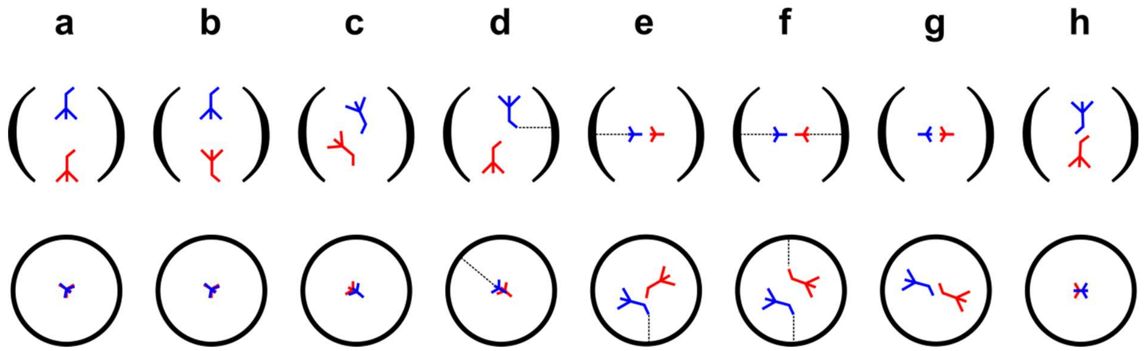

Figure S24. Potential solvent positions within the cavity of **cycHC[8]**. Methanol molecules in the cavity of **cycHC[8]** are blue and red. The top row (molecules in parentheses) is the **cycHC[8]** viewed from side. The bottom row (molecules inside a circle) is the **cycHC[8]** viewed from top. Dashed lines represent a hydrogen bond between the methanol and **cycHC[8]**.

The geometry where the second methanol molecule would form a hydrogen bond with the first methanol molecule (Figure S24 d) was energetically the most favoured geometry (and most calculations converged into d). The energies can be found in Table S12. The optimized geometry of structure d is depicted on Figure S25.

Table S12. Energy difference of studied cases.

Case	Total Energy (Hartrees)	Difference in kJ mol^{-1}
h	−4203.271087777	0.0
g	−4203.270983310	0.3
e	−4203.268209053	7.6
f	−4203.267332533	9.9
c	−4203.262990100	21.3
a	−4203.262137327	23.5
b	−4203.256909710	37.2
d	−4203.234933006	94.9

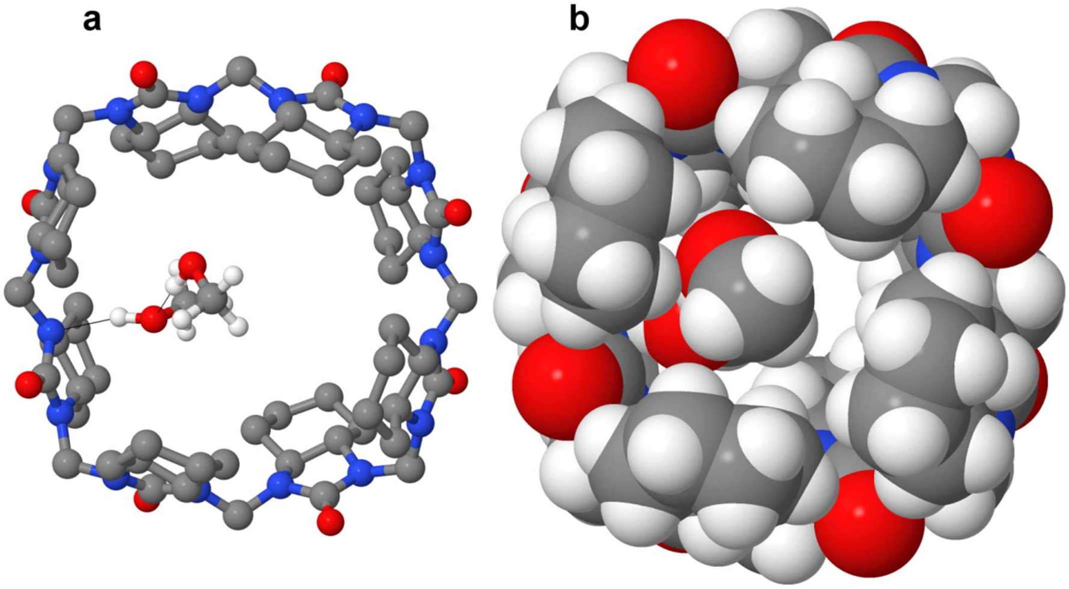

Figure S25. (a) (2MeOH)@**cycHC[8]** complex, where **cycHC[8]** is visualized without hydrogen atoms and hydrogen bonds as black lines; (b) (2MeOH)@**cycHC[8]** complex visualized with van der Waals radii.

As can be seen from the results (Figure S25 b), the hydrogen bond chain is preferred, thus the third methanol molecule would be connected *via* a hydrogen bond to the second one as depicted on Figure S26.[23]

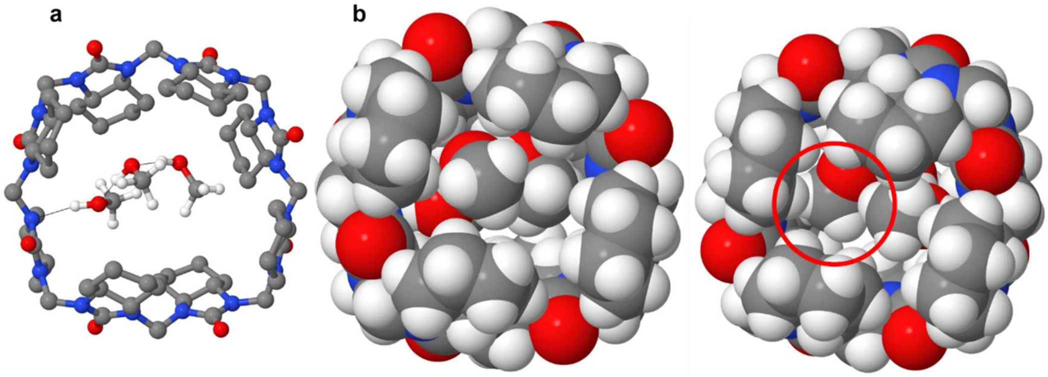

Figure S26. (a) (3MeOH)@**cycHC[8]** complex, where **cycHC[8]** is visualized without hydrogen atoms and hydrogen bonds as black lines; (b) (3MeOH)@**cycHC[8]** complex visualized with van der Waals viewed form the both portals. The red circle shows a cavity for a potential fourth MeOH molecule.

Although no fourth solvent molecule was observed in the experiment, the existence of a fourth solvent molecule was tested since the cavity potentially had room for a fourth solvent molecule (Figure S26 b).

Two cases were considered: the fourth solvent molecule is going to form a hydrogen bond with the third solvent molecule; the four solvent molecules form a hydrogen bond chain through the cavity as depicted on Figure S27. The second option was energetically favoured (Table S13).

Table S13. Energy difference of (4MeOH)@**cycHC[8]** complexes.

Complex	Energy (Hartrees)	Gibbs Correction (Hartrees)	Total Energy (Hartrees)	Energy difference in kJ mol^{-1}
Hydrogen bond chain trough cavity	−4434.540169381	1.644982	−4432.895187381	0.0
Hydrogen bond chain which is connected to cycHC[8]	−4434.545639636	1.650585	−4432.895054636	0.3

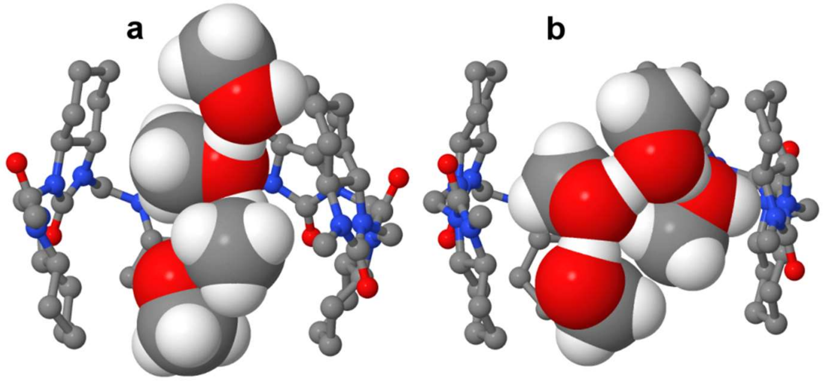

Figure S27. (a) the cross-section of (4MeOH)@**cycHC[8]** complex where four solvent molecules form a hydrogen bond chain through the cavity; (b) the cross-section view of (4MeOH)@**cycHC[8]** complex where the fourth solvent molecule forms a hydrogen bond with the third solvent molecule.

The total energies of the optimized geometries on different temperatures were compared to evaluate the number of solvent molecules in the cavity of **cycHC[8]**. The total energy was composed of the SCF energy and the Gibbs free energy correction (or zero point energy correction, where necessary). The total energies were compared for: (1MeOH)@**cycHC[8]** + 3 MeOH; (2MeOH)@**cycHC[8]** + 2 MeOH; (3MeOH)@**cycHC[8]** + 1 MeOH; (4MeOH)@**cycHC[8]** (Table S14). The results are depicted on Figure S28, where all energies are compared to the geometry of (1MeOH)@**cycHC[8]** on 300 K. At the room temperature the geometry with one solvent molecule was preferred. Thus, the complex with one methanol molecule (Figure S23) was chosen for further studies.

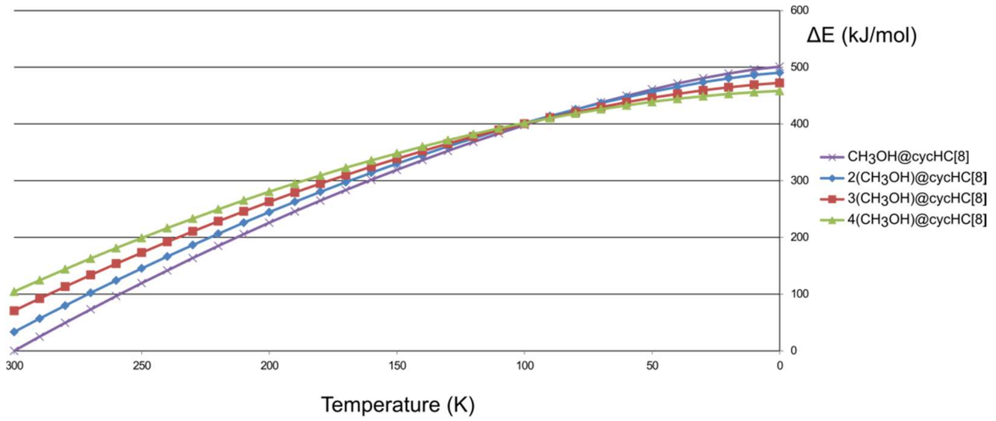

Figure S28. Temperature dependence of the number of solvent molecules in the cavity of **cycHC[8]**.

Table S14. The number of solvent molecules in the cavity of **cycHC[8]**. The total energies include the Gibbs free energy correction (except in the case of ZPE, where zero point energy correction was used). Total energies of solvents all include three solvent molecules (e.g. in the case of CH_3OH@**cycHC[8]** the total energy is obtained by adding together the total energy of CH_3OH@**cycHC[8]** and the total energy of three CH_3OH molecules.

Temperature	Total Energy of CH_3OH	Total energy of (CH_3OH) @cycHC[8]	Difference in kJ mol^{-1}	Total Energy of 2(CH_3OH) @cycHC[8]	Difference in kJ mol^{-1}	Total Energy of 3(CH_3OH) @cycHC[8]	Difference in kJ mol^{-1}	Total Energy of 4(CH_3OH) @cycHC[8]	Difference in kJ mol^{-1}
ZPE	−115.57908	−4432.74926	501	−4432.75332	490	−4432.76021	472	−4432.76551	458
10	−115.57941	−4432.75113	496	−4432.75487	486	−4432.76143	469	−4432.76640	456
20	−115.57991	−4432.75385	489	−4432.75712	480	−4432.76313	465	−4432.76760	453
30	−115.58048	−4432.75705	481	−4432.75979	473	−4432.76517	459	−4432.76908	449
40	−115.58109	−4432.76064	471	−4432.76281	465	−4432.76754	453	−4432.77085	444
50	−115.58174	−4432.76456	461	−4432.76617	457	−4432.77020	446	−4432.77290	439
60	−115.58241	−4432.76879	450	−4432.76982	447	−4432.77314	438	−4432.77522	433
70	−115.58310	−4432.77332	438	−4432.77377	437	−4432.77637	430	−4432.77782	426
80	−115.58381	−4432.77812	425	−4432.77798	426	−4432.77985	421	−4432.78067	419
90	−115.58453	−4432.78318	412	−4432.78246	414	−4432.78360	411	−4432.78378	410
100	−115.58527	−4432.78849	398	−4432.78718	401	−4432.78759	400	−4432.78714	402
110	−115.58602	−4432.79404	383	−4432.79215	388	−4432.79182	389	−4432.79074	392
120	−115.58679	−4432.79983	368	−4432.79736	375	−4432.79629	378	−4432.79458	382
130	−115.58757	−4432.80585	352	−4432.80280	360	−4432.80099	365	−4432.79865	371
140	−115.58835	−4432.81209	336	−4432.80846	346	−4432.80592	352	−4432.80294	360
150	−115.58915	−4432.81856	319	−4432.81435	330	−4432.81107	339	−4432.80746	348

160	−115.58996	−4432.82523	302	−4432.82045	314	−4432.81644	325	−4432.81220	336
170	−115.59077	−4432.83213	283	−4432.82677	298	−4432.82202	310	−4432.81715	323
180	−115.59160	−4432.83923	265	−4432.83329	280	−4432.82781	295	−4432.82232	309
190	−115.59243	−4432.84653	246	−4432.84003	263	−4432.83381	279	−4432.82770	295
200	−115.59327	−4432.85405	226	−4432.84697	244	−4432.84002	263	−4432.83328	280
210	−115.59411	−4432.86176	206	−4432.85411	226	−4432.84643	246	−4432.83907	265
220	−115.59497	−4432.86967	185	−4432.86145	206	−4432.85305	229	−4432.84507	249
230	−115.59583	−4432.87778	164	−4432.86900	187	−4432.85987	211	−4432.85127	233
240	−115.59670	−4432.88609	142	−4432.87675	166	−4432.86689	192	−4432.85767	216
250	−115.59757	−4432.89460	119	−4432.88469	145	−4432.87411	173	−4432.86427	199
260	−115.59845	−4432.90331	97	−4432.89283	124	−4432.88153	154	−4432.87107	181
270	−115.59934	−4432.91221	73	−4432.90118	102	−4432.88914	134	−4432.87808	163
280	−115.60023	−4432.92131	49	−4432.90972	80	−4432.89696	113	−4432.88528	144
290	−115.60113	−4432.93060	25	−4432.91845	57	−4432.90497	92	−4432.89268	124
300	−115.60204	−4432.94009	0	−4432.92738	33	−4432.91319	71	−4432.90028	105

Solvent molecule ejection and PF_6^- encapsulation to cycHC[8]

To study the guest exchange reaction (the solvent molecule ejection and the PF_6^- ion encapsulation) a series of geometry optimization calculations were done with partially frozen geometry (BP86/def2-SV(P)). The trajectory of the PF_6^- ion is depicted on Figure S29, where the frozen atoms are marked with an asterisk (*). All other atoms, including fluorine atoms were free to move during the optimization. The energy during the trajectory of the PF_6^- anion was calculated starting from the distance of 12.0 Å from the centre of the cavity and with each next calculation, the ion was brought closer to the centre of the cavity by 0.2 Å. The found minima were further studied using the BP86-D dispersion corrected functional.

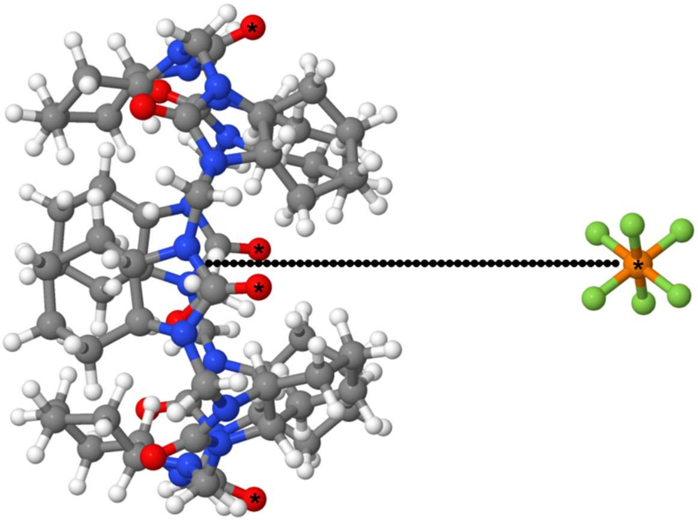

Figure S29. Trajectory of the PF_6^- ion, where frozen (fixed) atoms are marked with an asterisk (*).

Altogether, there were 61 calculations to assess the guest encapsulation path. The energies dependence upon distance between anion and host are depicted on Figure S30. The host and the guest start interacting with each other when the PF_6^- ion is approximately 10.0 Å from the centre of the cavity (Figure S30 a). If the PF_6^- ion overcomes a barrier (Figure S30 b), it forms a complex at the portal of **cycHC[8]** (MeOH@**cycHC[8]**+PF_6^-; Figure S30 c). During the next barrier (Figure S30 d) the hydrogen bond between the methanol and **cycHC[8]** is broken and the methanol is pushed toward the portal of **cycHC[8]** by PF_6^- ion (PF_6^-@**cycHC[8]**+ MeOH; Figure S30 e).

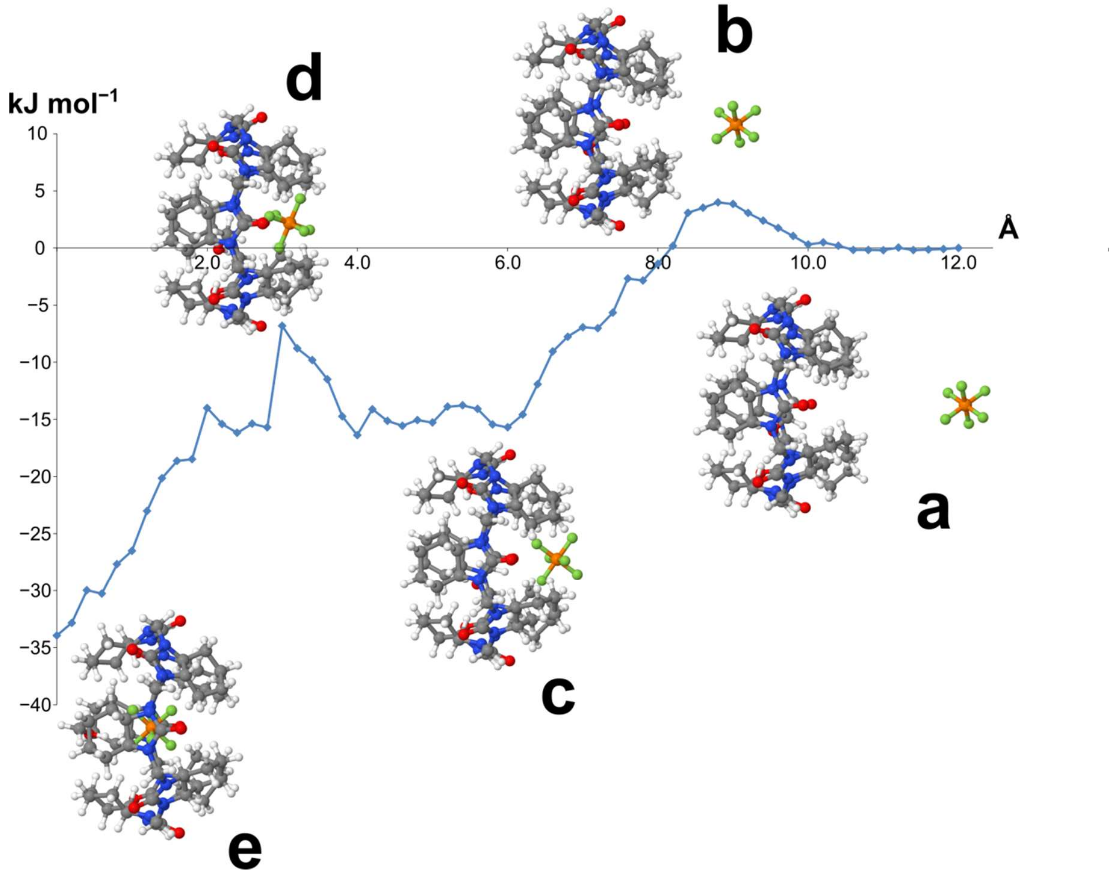

Figure S30. Energy graph of the reaction path: a) the host and the guest start interacting with each; b) the first barrier; c) complex of MeOH@**cycHC[8]**+PF_6^-; d) the second barrier; e) PF_6^-@**cycHC[8]**+ MeOH.

Optimized minima of the reaction

The geometries **a**, **c** and **e** on Figure S30 were taken for further study. They were optimized on BP86/SV(P) level of theory and vibrational analysis was performed to ensure all chosen geometries were at local minima. The energies were refined on BP86-D/TZVPD level of theory and the reaction path (without transition states) is depicted Figure S31. The total energies were obtained by adding the energies calculated on BP86-D/TZVPD level of theory and the Gibbs free energy correction from the vibrational analysis on BP86/SV(P) level of theory. The optimized complex **o-e** on is energetically 22 kJ mol^{-1} lower than **o-a**. The complex **o-c** is energetically 15 kJ mol^{-1} higher than **o-a**.

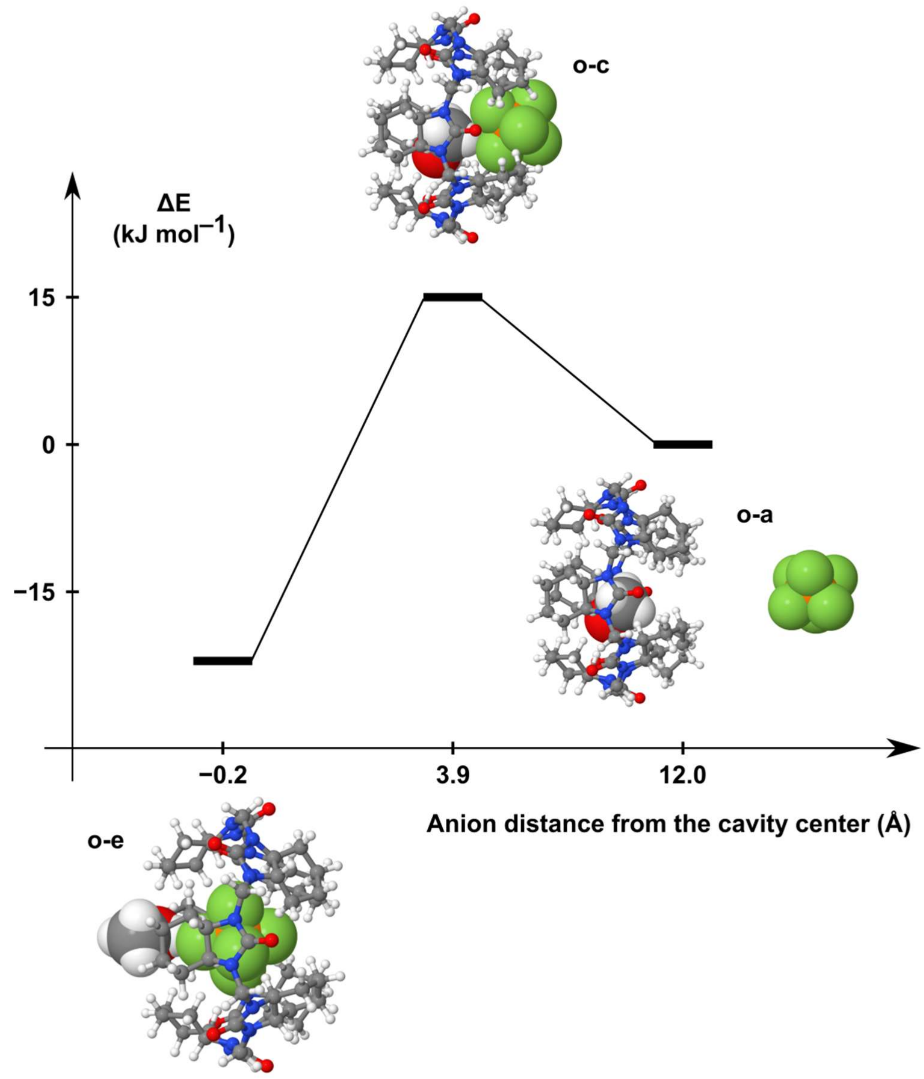

Figure S31. Energy graph of minima **o-a**, **o-c** and **o-e**, without transition states.

The geometry of **o-a** is depicted on Figure S32. During the optimization of **o-a**, the anion distance from the cavity center remained approximately 12.0 Å. According to Figure S30, the anion should not interact with the host. The methanol inside the cavity is sharing a hydrogen bond with the host. The geometry of **o-c** is depicted on Figure S33. The anion is situated on the opening of **cycHC[8]** (3.9 Å from the center of the cavity), while the methanol molecule is in the same position as in **o-a**. The geometry of **o-e** is depicted on Figure S34. After the hydrogen bond between the methanol molecule and the host is broken, the anion moves into the center of the cavity and replaces the methanol molecule. The methanol molecule is pushed out of the cavity, but it forms a new hydrogen bond with the PF_6^- ion and stays on the opening of **cycHC[8]**. The anion adopts the same position as in the crystal structure.

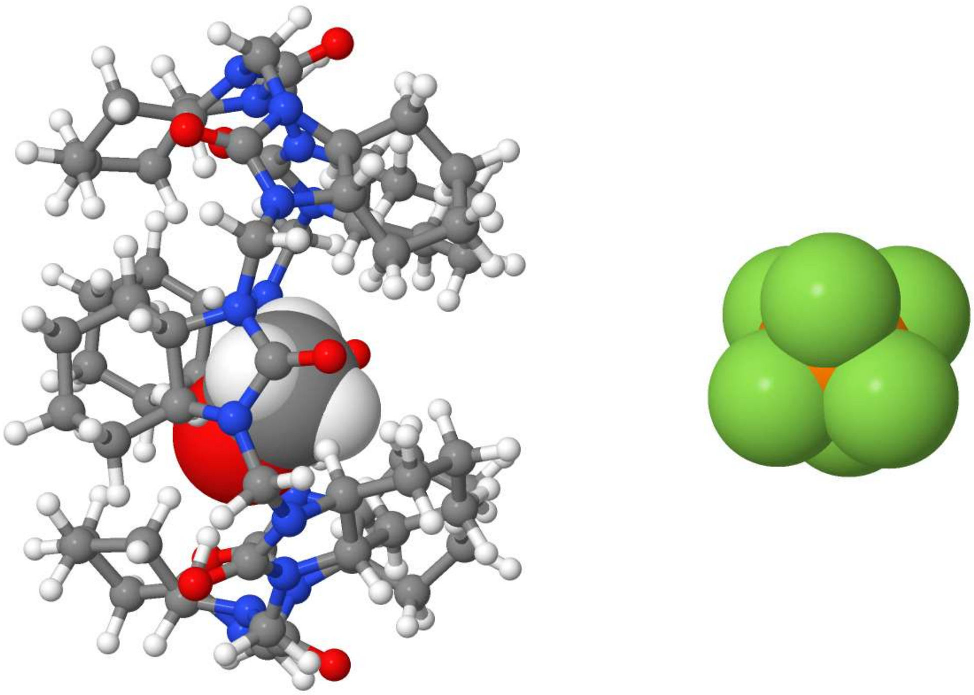

Figure S32. Minima of PF_6^- ion closing in on cycHC[8].

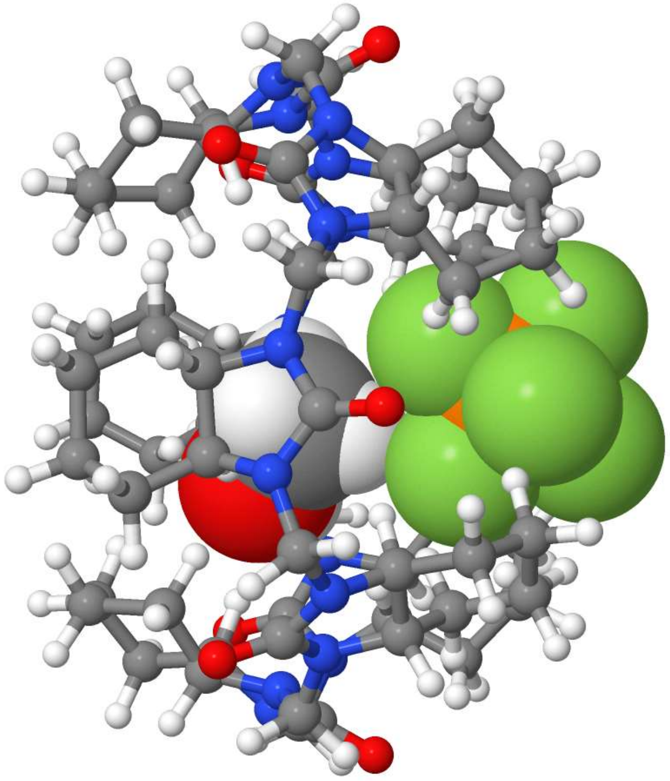

Figure S33. PF_6^- ion on the mouth of cycHC[8].

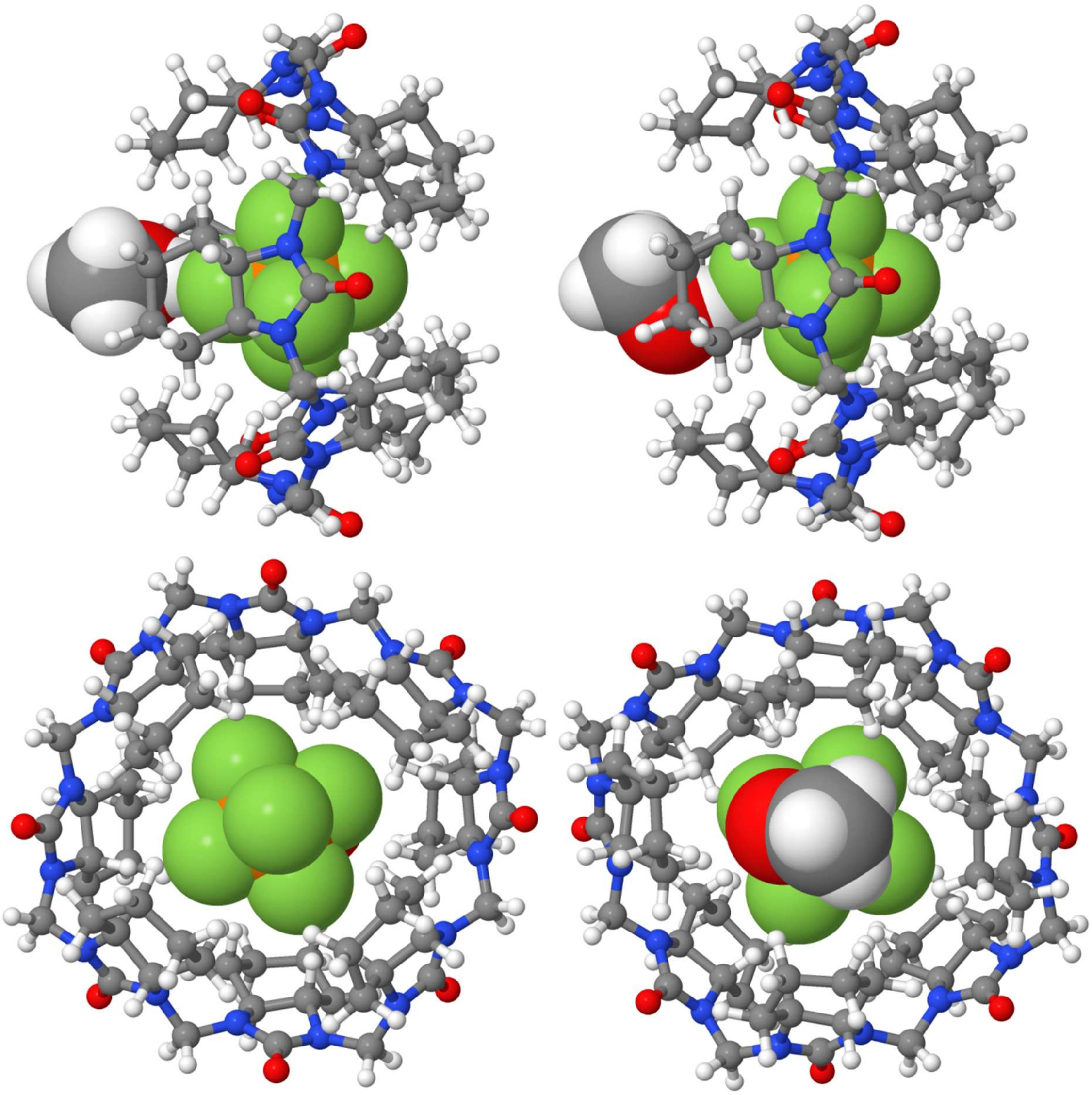

Figure S34. PF_6^- ion in the centre of the **cycHC[8]** cavity from different angles.

Optimized geometries

1MeOH@cycHC[8]

190

C	0.40423	-3.02267	-4.95289
C	-0.77011	-1.04661	-5.35538
H	-0.63705	-0.47799	-4.39698
C	-1.76161	-2.18858	-5.09604
H	-2.06621	-2.61079	-6.08950
C	-3.01943	-1.66478	-4.40904
H	-2.75542	-1.22405	-3.42161
H	-3.73671	-2.49101	-4.21110
C	-3.65072	-0.61724	-5.35856
H	-4.04650	-1.14557	-6.25746
H	-4.52738	-0.14461	-4.86298
C	-2.66034	0.47932	-5.81632
H	-2.41589	1.13331	-4.94660
H	-3.14966	1.13470	-6.57068
C	-1.33671	-0.08078	-6.39423
H	-1.51986	-0.61894	-7.35269
H	-0.62009	0.74468	-6.59349
C	-5.05339	-2.77982	1.74731
C	-3.63353	-1.06926	5.19055
C	-1.76517	-2.41498	4.87097
H	-1.79382	-3.05175	5.79399
C	-1.30250	-1.00907	5.27949
H	-1.03181	-0.44665	4.34657
C	-0.04672	-1.10141	6.14449
H	-0.26251	-1.69113	7.06515
H	0.29422	-0.09061	6.45636
C	1.04154	-1.79519	5.28707
H	1.33948	-1.09930	4.46768
H	1.95366	-1.95893	5.90246
C	0.58744	-3.13869	4.66762
H	0.48533	-3.89662	5.47923
H	1.37765	-3.51867	3.98276
C	-0.76158	-3.04877	3.91105
H	-0.66690	-2.42579	2.99225
H	-1.09730	-4.06048	3.59430
C	-2.66752	0.87179	6.41209
H	-3.71523	0.96276	6.76331
H	-1.98296	0.94606	7.28091
C	-1.06048	2.52668	5.47633
C	-3.06444	2.30606	4.29929
H	-2.82863	1.51107	3.54391
C	-2.35478	3.59784	3.86931
H	-2.78703	4.44521	4.46420
C	-2.62785	3.88976	2.39596
H	-2.26007	3.04786	1.76531
H	-2.09676	4.80982	2.06645
C	-4.16013	4.06506	2.25209
H	-4.42380	4.19787	1.17955
H	-4.45684	5.00903	2.76618
C	-4.97631	2.89078	2.84358
H	-6.06089	3.13542	2.80606
H	-4.83785	1.99166	2.19836
C	-4.57819	2.51573	4.29354
H	-4.85063	3.33014	5.00356
H	-5.10674	1.59103	4.61175
H	-2.32608	-4.69598	-4.58280
C	0.04569	4.37036	4.25099
H	-0.42608	5.37485	4.17877
C	1.02946	5.25197	2.15847
C	2.21536	3.54512	3.19858
H	2.85368	4.12792	3.91394
C	2.77770	3.75202	1.78575
H	2.31233	2.98658	1.10991
C	4.28721	3.51530	1.78388
H	4.78400	4.23219	2.47725
H	4.71053	3.66989	0.76776
C	4.51521	2.05377	2.24435
H	4.14428	1.37159	1.44294
H	5.60616	1.85732	2.33787
C	3.80920	1.69854	3.57470
H	4.31892	2.23235	4.41057
H	3.92799	0.61149	3.77942
C	2.30732	2.07502	3.59820
H	1.72656	1.44880	2.88260
H	1.87803	1.91038	4.61093
C	2.50838	5.79786	0.22934
H	1.98870	6.77291	0.31752
H	3.60090	5.97063	0.15427
C	2.97688	4.31385	-1.71461
C	0.72593	4.82059	-1.36896
H	0.38011	3.97877	-0.71241
C	0.91597	4.26847	-2.78919
H	1.01827	5.13917	-3.48936
C	-0.31560	3.47758	-3.22246
H	-0.48482	2.62306	-2.52803
H	-0.18170	3.05795	-4.24374
C	-1.51216	4.46066	-3.19650
H	-2.45264	3.91293	-3.42707
H	-1.37374	5.20392	-4.01600
C	-1.66703	5.21304	-1.85363
H	-2.47267	5.97476	-1.94498
H	-2.00523	4.49366	-1.07117
C	-0.36362	5.89126	-1.36232
H	-0.07482	6.73055	-2.03615
H	-0.50470	6.30353	-0.33981
C	2.88529	3.00379	-3.81123
H	2.52979	3.47354	-4.75493
H	3.97681	3.18616	-3.69636
C	2.10352	1.04723	-5.10907
C	3.53083	0.57972	-3.33475
H	4.53166	0.67165	-3.83369
C	2.88200	-0.72771	-3.81002
H	2.00813	-0.94501	-3.13851
C	3.86908	-1.88551	-3.67397
H	4.77322	-1.68810	-4.29457
H	3.41238	-2.83642	-4.02287
C	4.24102	-1.98708	-2.17341
H	3.34391	-2.33968	-1.61200
H	5.02069	-2.76810	-2.03322
C	4.72855	-0.65276	-1.55990
H	5.73021	-0.40322	-1.98140
H	4.87376	-0.77812	-0.46399
C	3.77490	0.53831	-1.82824
H	2.80933	0.40801	-1.28851
H	4.23054	1.48835	-1.47289
C	1.71673	-1.21989	-6.06408
H	2.39541	-2.06643	-6.29082
H	1.54260	-0.63460	-6.98951
N	0.45788	-1.80336	-5.62634
N	-0.89070	-3.17405	-4.43531
N	-3.14802	-2.15607	4.45846
N	-2.54812	-0.45041	5.81672
N	-2.35117	1.99505	5.54275
N	-0.99167	3.35762	4.35433
N	0.91887	4.22306	3.09868
N	2.22139	5.06758	1.45378
N	2.10920	5.15266	-1.01182
N	2.22910	3.62553	-2.67450
N	2.63622	1.57743	-3.93023
N	2.37211	-0.32404	-5.12434
O	1.32494	-3.84076	-4.84491
O	-4.81849	-0.72652	5.28017
O	-0.14339	2.31490	6.27878
O	0.22072	6.17370	1.99466
O	4.19588	4.20762	-1.53345
O	1.51375	1.68467	-5.98897
H	-6.07765	-3.11507	2.06133
H	0.65203	4.31344	5.18183
C	-4.04194	-3.22463	4.04620
C	-1.37126	-4.48885	-4.05352
C	-2.93734	-5.01626	-2.20913
C	-0.68815	-5.41293	-1.77898
H	-0.62221	-6.44565	-2.20899
C	-1.46651	-5.50073	-0.46036
H	-1.36351	-4.52167	0.07391
C	-0.87699	-6.59558	0.42729
H	-0.95044	-7.58033	-0.08875
H	-1.43484	-6.66236	1.38573
C	0.60285	-6.22368	0.69058
H	0.62821	-5.31232	1.33315
H	1.09239	-7.03232	1.27700
C	1.41293	-5.95147	-0.59766
H	1.54709	-6.90872	-1.15379
H	2.43364	-5.59932	-0.33027
C	0.73752	-4.92457	-1.53971
H	0.72586	-3.91446	-1.07002
H	1.29348	-4.83906	-2.49821
C	-4.01278	-5.84008	-0.12231
H	-4.86432	-6.00993	-0.81106
H	-3.84865	-6.75083	0.48771
C	-3.81484	-4.71856	2.08857
C	-4.73428	-3.41185	0.38586
H	-3.83399	-2.89877	-0.03804
C	-5.08465	-1.25780	1.62922
H	-4.09612	-0.88514	1.27843
H	-5.29498	-0.78538	2.61415
C	-6.20093	-0.91047	0.61318
H	-5.05850	-2.95353	4.40903
H	-6.21226	0.18680	0.42880
H	-7.18626	-1.16057	1.07257
C	-6.06593	-1.66033	-0.73366
H	-3.72489	-4.18176	4.51694
H	-6.95151	-1.44196	-1.37083
H	-5.17753	-1.26801	-1.28060
C	-5.89872	-3.19303	-0.57843
H	-6.82757	-3.65516	-0.17129
H	-5.68682	-3.65533	-1.56682
H	-0.60779	-5.23519	-4.36189
N	-1.62830	-4.64238	-2.61788
N	-2.84125	-5.61528	-0.96155
N	-4.34823	-4.77024	0.79928
N	-4.07675	-3.44981	2.61251
O	-3.96440	-4.83768	-2.86677
O	-3.22920	-5.63786	2.67448
H	-0.14230	-1.98007	-0.04461
C	-0.74981	-1.34568	-0.73771
H	-0.08670	-1.02325	-1.57988
H	-1.05799	-0.43651	-0.17777
O	-1.92822	-1.98934	-1.18909
H	-1.68591	-2.76843	-1.75837

(2MeOH)@cycHC[8] a

196

C	0.12750	-2.96586	-5.16474
C	-1.10980	-0.98763	-5.17326
H	-0.81640	-0.50737	-4.20127
C	-2.03585	-2.17062	-4.85828
H	-2.52366	-2.49379	-5.81585
C	-3.14641	-1.73994	-3.90335
H	-2.70651	-1.37066	-2.94903
H	-3.81209	-2.59569	-3.65642
C	-3.94475	-0.62171	-4.61741
H	-4.49741	-1.07500	-5.47328
H	-4.71751	-0.21323	-3.92859
C	-3.05942	0.53255	-5.14421
H	-2.66723	1.11519	-4.27745
H	-3.68243	1.23876	-5.73618
C	-1.85687	0.05872	-5.99879
H	-2.20421	-0.39116	-6.95705
H	-1.19392	0.91721	-6.24019
C	-5.26695	-2.99137	1.54838
C	-3.95043	-1.03368	4.91836
C	-2.05627	-2.37887	4.81101
H	-2.15357	-2.97652	5.75550
C	-1.63335	-0.95351	5.19296
H	-1.28212	-0.43231	4.26366
C	-0.45734	-1.00371	6.16618
H	-0.75303	-1.55570	7.08824
H	-0.14617	0.02076	6.46462
C	0.70139	-1.72528	5.43325
H	1.07743	-1.06723	4.61657
H	1.55449	-1.86516	6.13354
C	0.31148	-3.09135	4.81976
H	0.15919	-3.83409	5.63746
H	1.15839	-3.45978	4.20092
C	-0.97659	-3.04182	3.96024
H	-0.81403	-2.45000	3.03004
H	-1.28249	-4.06615	3.65403
C	-3.09204	0.96826	6.12367
H	-4.16884	1.07225	6.36688
H	-2.49470	1.07674	7.05125
C	-1.39873	2.59636	5.29667
C	-3.26948	2.32485	3.92914
H	-2.94613	1.51289	3.22561
C	-2.53041	3.61322	3.53878
H	-3.02671	4.47116	4.06439
C	-2.65441	3.86483	2.03820
H	-2.20965	3.01477	1.47112
H	-2.10585	4.78598	1.74183
C	-4.16578	4.01109	1.73121
H	-4.31835	4.11439	0.63390
H	-4.52707	4.96203	2.18768
C	-5.02306	2.83864	2.26473
H	-6.10077	3.06303	2.10431
H	-4.80322	1.92471	1.66409
C	-4.77622	2.51194	3.75888
H	-5.13133	3.34294	4.41074
H	-5.32562	1.58989	4.04867
H	-2.42517	-4.79558	-4.70170
C	-0.17265	4.40027	4.12521
H	-0.62480	5.41135	4.02068
C	0.96977	5.26709	2.10749
C	2.00798	3.47574	3.16946
H	2.65003	4.00317	3.92447
C	2.63691	3.66530	1.78272
H	2.15033	2.94537	1.07539
C	4.12762	3.33711	1.83654
H	4.64212	4.00370	2.56695
H	4.60152	3.48270	0.84178
C	4.24054	1.85342	2.26498
H	3.85700	1.22316	1.42919
H	5.31119	1.58136	2.39893
C	3.45497	1.50728	3.55319

H	3.97178	1.96049	4.43141
H	3.46855	0.40554	3.70224
C	1.98497	1.99502	3.53759
H	1.38792	1.42801	2.78848
H	1.51399	1.83635	4.53258
C	2.54436	5.72799	0.23941
H	2.10895	6.74458	0.31202
H	3.64811	5.80690	0.17750
C	2.88673	4.19755	-1.69432
C	0.70172	4.97600	-1.42047
H	0.21776	4.18684	-0.78858
C	0.87678	4.41800	-2.83825
H	1.16722	5.26859	-3.50799
C	-0.43613	3.84818	-3.36688
H	-0.79469	3.02539	-2.70720
H	-0.30259	3.42362	-4.38547
C	-1.45761	5.01162	-3.38251
H	-2.45581	4.62916	-3.69017
H	-1.14844	5.74470	-4.16348
C	-1.57609	5.73963	-2.02350
H	-2.25203	6.61712	-2.12622
H	-2.06155	5.05648	-1.28704
C	-0.21557	6.19779	-1.44327
H	0.23553	6.99641	-2.07586
H	-0.35003	6.60384	-0.41775
C	2.72779	2.98745	-3.84693
H	2.37589	3.52941	-4.75151
H	3.82008	3.14667	-3.72227
C	1.74324	1.14433	-5.16518
C	3.49034	0.54852	-3.75315
H	4.35644	0.71682	-4.44608
C	2.76459	-0.72335	-4.21089
H	2.04143	-1.01710	-3.40596
C	3.75835	-1.87014	-4.38497
H	4.51416	-1.60272	-5.15802
H	3.23739	-2.79415	-4.71709
C	4.43539	-2.09711	-3.01090
H	3.67436	-2.50945	-2.30646
H	5.22401	-2.87578	-3.10725
C	5.04320	-0.81270	-2.39921
H	5.92941	-0.50726	-3.00404
H	5.42454	-1.03112	-1.37710
C	4.04979	0.37354	-2.34415
H	3.21939	0.18375	-1.62841
H	4.56526	1.29807	-2.00334
C	1.22719	-1.02516	-6.27666
H	1.87449	-1.82448	-6.69038
H	0.90673	-0.35412	-7.09874
N	0.05774	-1.68586	-5.71984
N	-1.06639	-3.20218	-4.47593
N	-3.40520	-2.15232	4.28036
N	-2.92058	-0.37580	5.59412
N	-2.68736	2.05761	5.24830
N	-1.22265	3.39643	4.16448
N	0.76257	4.23767	3.02757
N	2.18166	5.02467	1.45559
N	2.10303	5.11203	-1.00669
N	2.06842	3.56133	-2.66784
N	2.47393	1.57279	-4.04650
N	2.00743	-0.21003	-5.35823
O	1.06953	-3.75933	-5.27502
O	-5.14085	-0.69813	4.90023
O	-0.56494	2.41240	6.19125
O	0.21923	6.23261	1.91677
O	4.08475	3.96835	-1.51574
O	1.01293	1.85509	-5.86496
H	-6.26225	-3.39580	1.87139
H	0.37090	4.33774	5.09403
C	-4.26107	-3.25453	3.87519
C	-1.47959	-4.56014	-4.16418

C	-2.97632	-5.22841	-2.30959
C	-0.69868	-5.35861	-1.86732
H	-0.47098	-6.39866	-2.22074
C	-1.46932	-5.46484	-0.54404
H	-1.48260	-4.44968	-0.06690
C	-0.74801	-6.41619	0.40912
H	-0.68292	-7.43109	-0.04563
H	-1.29830	-6.50234	1.37060
C	0.66548	-5.82982	0.65191
H	0.56087	-4.89544	1.25234
H	1.25952	-6.53328	1.27599
C	1.44171	-5.50899	-0.64730
H	1.72439	-6.46430	-1.14822
H	2.39699	-4.99780	-0.39390
C	0.63586	-4.64939	-1.65294
H	0.46323	-3.62306	-1.25403
H	1.19165	-4.54555	-2.61049
C	-3.96228	-6.04343	-0.17402
H	-4.79903	-6.32171	-0.84535
H	-3.72363	-6.89974	0.48798
C	-3.88193	-4.80610	1.98656
C	-4.89184	-3.66249	0.21996
H	-4.03855	-3.09097	-0.23092
C	-5.42709	-1.48510	1.35809
H	-4.47282	-1.04460	0.98875
H	-5.67578	-0.98706	2.32090
C	-6.56738	-1.28265	0.32962
H	-5.29677	-2.99729	4.18918
H	-6.67042	-0.20097	0.09088
H	-7.52822	-1.59107	0.80440
C	-6.37080	-2.08481	-0.97881
H	-3.93729	-4.18302	4.39530
H	-7.27358	-1.97810	-1.61958
H	-5.52614	-1.63829	-1.55474
C	-6.06670	-3.58716	-0.75389
H	-6.95060	-4.11037	-0.32256
H	-5.81131	-4.07682	-1.71832
H	-0.67314	-5.23664	-4.52380
N	-1.72313	-4.79570	-2.75222
N	-2.81872	-5.77044	-1.03177
N	-4.38901	-4.95655	0.69377
N	-4.24638	-3.53744	2.44935
O	-4.03381	-5.15700	-2.94669
O	-3.23921	-5.65160	2.62029
C	-0.06031	0.89158	-0.82776
O	1.24795	1.41058	-0.67579
H	-0.19363	0.33049	-1.78668
H	-0.85388	1.67939	-0.77504
H	-0.23732	0.18312	0.00950
H	1.46479	1.99493	-1.45149
H	2.16132	-0.59275	0.92064
C	2.15992	-1.66849	1.22368
H	2.60858	-2.26723	0.39158
H	1.10274	-1.98957	1.34140
O	2.80324	-1.90531	2.46936
H	3.74649	-1.65010	2.37858

(2MeOH)@cycHC[8] b

196

C	0.28746	-2.93874	-5.23449
C	-0.92708	-0.95173	-5.38042
H	-0.67657	-0.43386	-4.41616
C	-1.87908	-2.11400	-5.06424
H	-2.32573	-2.46909	-6.03002
C	-3.02760	-1.63588	-4.17961
H	-2.62283	-1.23187	-3.22245
H	-3.71143	-2.47490	-3.92537
C	-3.78152	-0.54059	-4.97358

H	-4.29891	-1.02529	-5.83398
H	-4.58087	-0.09750	-4.33892
C	-2.86152	0.58310	-5.50726
H	-2.50488	1.20008	-4.64871
H	-3.44982	1.26847	-6.15637
C	-1.62398	0.06545	-6.28317
H	-1.92941	-0.42074	-7.23796
H	-0.94238	0.90846	-6.52779
C	-5.18902	-2.83106	1.47591
C	-3.88863	-0.98839	4.88378
C	-2.04557	-2.40505	4.84747
H	-2.20066	-2.99064	5.79268
C	-1.58039	-0.99416	5.23416
H	-1.17815	-0.49506	4.31492
C	-0.44756	-1.07965	6.25502
H	-0.80583	-1.59930	7.17350
H	-0.10270	-0.06368	6.54530
C	0.70771	-1.86616	5.58715
H	1.15666	-1.23746	4.78462
H	1.51948	-2.03824	6.32810
C	0.28117	-3.22015	4.97131
H	0.04960	-3.94177	5.78923
H	1.14187	-3.64169	4.40742
C	-0.95870	-3.11911	4.04746
H	-0.72417	-2.53973	3.12432
H	-1.29480	-4.13006	3.72866
C	-2.99027	0.98669	6.10871
H	-4.06144	1.12158	6.36133
H	-2.38315	1.08000	7.03148
C	-1.26485	2.55659	5.23111
C	-3.20928	2.39977	3.94831
H	-2.95227	1.60881	3.19839
C	-2.44479	3.67705	3.57136
H	-2.87755	4.53082	4.15775
C	-2.64210	3.99235	2.09091
H	-2.28237	3.13598	1.47635
H	-2.06686	4.89660	1.79287
C	-4.16069	4.22269	1.88640
H	-4.37610	4.38686	0.80698
H	-4.44930	5.16536	2.40779
C	-5.03796	3.06407	2.41872
H	-6.11203	3.34276	2.33811
H	-4.89987	2.17144	1.76453
C	-4.71452	2.65169	3.87655
H	-4.99949	3.46076	4.58821
H	-5.28109	1.73547	4.15139
H	-2.32579	-4.70863	-4.80965
C	-0.03734	4.36108	4.06653
H	-0.46302	5.38772	4.01611
C	1.03463	5.26802	2.02803
C	2.09690	3.44482	3.00658
H	2.76478	3.94742	3.75529
C	2.68869	3.67700	1.60938
H	2.18234	2.97284	0.89711
C	4.17897	3.34099	1.60916
H	4.71343	3.98388	2.34569
H	4.62603	3.52004	0.60728
C	4.29922	1.84517	1.99143
H	3.89543	1.23750	1.14653
H	5.37227	1.56734	2.08500
C	3.55072	1.46263	3.29140
H	4.09308	1.89858	4.16298
H	3.57268	0.35729	3.41941
C	2.08205	1.95382	3.33330
H	1.45537	1.40943	2.59036
H	1.63889	1.76868	4.33645
C	2.55048	5.78015	0.12032
H	2.08397	6.77974	0.23086
H	3.65063	5.89567	0.04802
C	2.94447	4.32462	-1.86305

C	0.72031	4.92505	-1.49424
H	0.33728	4.08383	-0.85634
C	0.87993	4.39984	-2.92810
H	1.01922	5.28216	-3.60697
C	-0.38755	3.67710	-3.37612
H	-0.59046	2.81337	-2.70110
H	-0.27491	3.27487	-4.40680
C	-1.53946	4.71061	-3.31885
H	-2.50478	4.21196	-3.55804
H	-1.37129	5.46692	-4.12026
C	-1.65418	5.43632	-1.95740
H	-2.42604	6.23453	-2.02555
H	-2.02090	4.71479	-1.18952
C	-0.31965	6.04351	-1.45722
H	0.00443	6.88371	-2.11347
H	-0.43771	6.43902	-0.42533
C	2.78688	3.08530	-3.99882
H	2.42069	3.58677	-4.92156
H	3.88327	3.24530	-3.90195
C	1.90813	1.18173	-5.31339
C	3.44963	0.64532	-3.65844
H	4.41627	0.76407	-4.21567
C	2.77572	-0.64410	-4.14759
H	1.95049	-0.89834	-3.43049
C	3.77528	-1.79925	-4.13108
H	4.63354	-1.56713	-4.80239
H	3.30065	-2.73750	-4.49078
C	4.25076	-1.96503	-2.66613
H	3.39693	-2.35385	-2.06217
H	5.04436	-2.74291	-2.61549
C	4.76830	-0.65506	-2.02506
H	5.73790	-0.37625	-2.49964
H	4.99009	-0.82880	-0.94868
C	3.79141	0.53798	-2.17455
H	2.86216	0.37342	-1.58180
H	4.26067	1.47538	-1.80401
C	1.46910	-1.04557	-6.34105
H	2.14220	-1.86505	-6.66425
H	1.21520	-0.41438	-7.21642
N	0.25941	-1.67967	-5.84001
N	-0.94096	-3.13936	-4.59695
N	-3.36495	-2.12952	4.26884
N	-2.85693	-0.36483	5.58797
N	-2.57068	2.06521	5.22828
N	-1.11400	3.38834	4.11639
N	0.85080	4.21375	2.92537
N	2.22232	5.03774	1.32701
N	2.11810	5.18364	-1.13488
N	2.16213	3.69375	-2.83625
N	2.51674	1.66485	-4.14985
N	2.17664	-0.18604	-5.40465
O	1.22565	-3.74369	-5.26906
O	-5.06622	-0.61256	4.83198
O	-0.39540	2.31887	6.07885
O	0.28591	6.24314	1.88993
O	4.15809	4.16052	-1.69205
O	1.26157	1.85402	-6.12507
H	-6.20567	-3.21239	1.76001
H	0.54579	4.24876	5.00713
C	-4.24577	-3.19257	3.81811
C	-1.38440	-4.48284	-4.26140
C	-2.92575	-5.08391	-2.42175
C	-0.65948	-5.28902	-1.95126
H	-0.46044	-6.33451	-2.30577
C	-1.44984	-5.37558	-0.63832
H	-1.43403	-4.36357	-0.15443
C	-0.77394	-6.35741	0.31745
H	-0.73727	-7.37068	-0.14400
H	-1.33969	-6.43175	1.27100
C	0.65522	-5.82150	0.58238

H	0.57417	-4.88903	1.18945
H	1.21763	-6.54924	1.20794
C	1.45831	-5.51634	-0.70419
H	1.71811	-6.47652	-1.20802
H	2.42601	-5.03754	-0.43444
C	0.69457	-4.62502	-1.71527
H	0.54846	-3.59570	-1.31287
H	1.26541	-4.53392	-2.66511
C	-3.96285	-5.88256	-0.30159
H	-4.80092	-6.11738	-0.98790
H	-3.76105	-6.76076	0.34423
C	-3.87913	-4.70589	1.89484
C	-4.80075	-3.47595	0.13841
H	-3.91408	-2.92294	-0.26870
C	-5.29012	-1.31526	1.32790
H	-4.30878	-0.89373	1.01089
H	-5.55547	-0.84145	2.29861
C	-6.39133	-1.04237	0.27303
H	-5.27970	-2.91075	4.11687
H	-6.45139	0.04938	0.06563
H	-7.37781	-1.33182	0.70596
C	-6.18285	-1.80937	-1.05446
H	-3.96276	-4.14376	4.32046
H	-7.05954	-1.64923	-1.72014
H	-5.30365	-1.37830	-1.58929
C	-5.94195	-3.32808	-0.86648
H	-6.85778	-3.82821	-0.47597
H	-5.67796	-3.80075	-1.83717
H	-0.58695	-5.18311	-4.59399
N	-1.65283	-4.68999	-2.84849
N	-2.80218	-5.63176	-1.14341
N	-4.35973	-4.80137	0.58583
N	-4.21229	-3.44078	2.38578
O	-3.97137	-4.97967	-3.07370
O	-3.28155	-5.59241	2.51737
C	-1.31482	0.39600	-0.26483
O	-2.36163	0.65545	0.65798
H	-0.74818	1.31927	-0.54429
H	-1.68067	-0.08821	-1.20389
H	-0.60781	-0.30113	0.23257
H	-3.04143	1.20405	0.21080
H	3.00397	-1.29584	1.22472
C	2.70091	-2.18901	1.82681
H	3.01720	-3.10711	1.27320
H	1.59195	-2.19270	1.89900
O	3.20306	-2.15503	3.15320
H	4.18388	-2.18420	3.10826

(2MeOH)@cycHC[8] c

196

C	0.12750	-2.96586	-5.16474
C	-1.14798	-1.02170	-5.35097
H	-0.97307	-0.51246	-4.36549
C	-2.07217	-2.22337	-5.10825
H	-2.43676	-2.58540	-6.10529
C	-3.29795	-1.80483	-4.30254
H	-2.97858	-1.39555	-3.31657
H	-3.96167	-2.67326	-4.09880
C	-4.03988	-0.73046	-5.13538
H	-4.47134	-1.22207	-6.03813
H	-4.90175	-0.33180	-4.55565
C	-3.13103	0.43895	-5.58369
H	-2.85959	1.05189	-4.69160
H	-3.69994	1.11482	-6.25944
C	-1.82385	-0.01503	-6.28026
H	-2.04413	-0.49342	-7.26209
H	-1.16016	0.85761	-6.46209
C	-5.38377	-3.07474	1.59567

C	-3.95641	-1.05339	4.92265
C	-2.08745	-2.43287	4.84518
H	-2.20583	-3.00858	5.80098
C	-1.63969	-1.00932	5.20299
H	-1.28049	-0.51037	4.26461
C	-0.46533	-1.05861	6.17878
H	-0.76958	-1.58563	7.11229
H	-0.13715	-0.03309	6.45411
C	0.68206	-1.81771	5.46582
H	1.05999	-1.17952	4.63224
H	1.53527	-1.94928	6.16711
C	0.26142	-3.19385	4.89655
H	0.07547	-3.89599	5.74232
H	1.10226	-3.62794	4.31118
C	-1.01408	-3.13702	4.01994
H	-0.82646	-2.56871	3.07949
H	-1.33925	-4.15970	3.73019
C	-3.06776	0.94254	6.12040
H	-4.14049	1.06010	6.37524
H	-2.45959	1.04460	7.04160
C	-1.36605	2.54897	5.26465
C	-3.27401	2.32140	3.94054
H	-2.97879	1.51864	3.21513
C	-2.52889	3.60726	3.55372
H	-2.99917	4.46199	4.10774
C	-2.69197	3.88564	2.06192
H	-2.28337	3.03152	1.47427
H	-2.13558	4.80190	1.76449
C	-4.20925	4.06471	1.80430
H	-4.39525	4.19685	0.71500
H	-4.54116	5.01057	2.29240
C	-5.06847	2.89364	2.33734
H	-6.14699	3.13616	2.21337
H	-4.87987	1.99008	1.71061
C	-4.78244	2.53042	3.81579
H	-5.10914	3.35135	4.49465
H	-5.33498	1.60874	4.10068
H	-2.47189	-4.81586	-4.75189
C	-0.14970	4.35947	4.09571
H	-0.59719	5.37488	4.01565
C	0.96929	5.26230	2.08301
C	2.01913	3.45372	3.09611
H	2.66504	3.97552	3.85194
C	2.63468	3.67345	1.70662
H	2.14195	2.96313	0.99442
C	4.12560	3.34243	1.73443
H	4.64981	4.00275	2.46372
H	4.58400	3.50231	0.73420
C	4.24711	1.85379	2.14626
H	3.83988	1.22976	1.31774
H	5.32075	1.58244	2.25552
C	3.48834	1.50539	3.44914
H	4.00581	1.98292	4.31469
H	3.53738	0.40728	3.62360
C	2.00980	1.96662	3.43916
H	1.43160	1.40871	2.66906
H	1.53886	1.78918	4.43122
C	2.51807	5.79253	0.22185
H	2.07168	6.79950	0.34654
H	3.62015	5.88559	0.14962
C	2.89077	4.34744	-1.76403
C	0.67057	4.93937	-1.36248
H	0.31254	4.11495	-0.68864
C	0.80126	4.36935	-2.78200
H	0.89987	5.22980	-3.49522
C	-0.46520	3.60873	-3.16827
H	-0.62694	2.76501	-2.45504
H	-0.37251	3.16443	-4.18331
C	-1.63334	4.62371	-3.11828
H	-2.59747	4.10374	-3.31397
H	-1.49828	5.35007	-3.95268
C	-1.72208	5.40172	-1.78417
H	-2.50888	6.18417	-1.86071
H	-2.05984	4.70702	-0.97725
C	-0.38463	6.04486	-1.34033
H	-0.09044	6.86373	-2.03599
H	-0.48313	6.47476	-0.32076
C	2.69850	3.06154	-3.86711
H	2.32816	3.56018	-4.79029
H	3.79735	3.20830	-3.78270
C	1.72042	1.17135	-5.11429
C	3.36383	0.61710	-3.56378
H	4.29512	0.73057	-4.18068
C	2.65151	-0.66424	-4.01913
H	1.86759	-0.91251	-3.25736
C	3.64159	-1.82594	-4.06221
H	4.46646	-1.59590	-4.77510
H	3.14243	-2.75856	-4.40296
C	4.19069	-2.00105	-2.62390
H	3.36494	-2.38060	-1.97689
H	4.97729	-2.78818	-2.61786
C	4.75511	-0.69774	-2.00822
H	5.70321	-0.43234	-2.53355
H	5.02901	-0.87815	-0.94501
C	3.78414	0.50616	-2.10115
H	2.89019	0.36759	-1.45071
H	4.29249	1.43679	-1.76529
C	1.29628	-1.03015	-6.20644
H	1.98668	-1.82814	-6.54711
H	1.04349	-0.37629	-7.06517
N	0.08782	-1.70067	-5.75269
N	-1.12232	-3.21214	-4.58711
N	-3.42721	-2.18848	4.29747
N	-2.91747	-0.40536	5.59380
N	-2.66161	2.02634	5.23994
N	-1.20708	3.36314	4.13943
N	0.76696	4.21073	2.97942
N	2.17396	5.03805	1.41504
N	2.07214	5.22159	-1.04138
N	2.09606	3.69159	-2.70626
N	2.41066	1.64482	-3.99690
N	1.98391	-0.19240	-5.23898
O	1.08954	-3.74312	-5.16772
O	-5.14085	-0.69813	4.90023
O	-0.51408	2.34398	6.13733
O	0.21923	6.23261	1.91677
O	4.10808	4.19777	-1.61088
O	1.01293	1.85509	-5.86496
H	-6.34119	-3.55481	1.92909
H	0.41070	4.27771	5.05340
C	-4.29832	-3.28225	3.89684
C	-1.52424	-4.56168	-4.22892
C	-2.99956	-5.19154	-2.33980
C	-0.71525	-5.31538	-1.92906
H	-0.49257	-6.35866	-2.27640
C	-1.46562	-5.41121	-0.59386
H	-1.47044	-4.39311	-0.12276
C	-0.72962	-6.35616	0.35479
H	-0.66947	-7.37356	-0.09506
H	-1.26629	-6.43749	1.32432
C	0.68662	-5.76696	0.57383
H	0.58892	-4.82873	1.16944
H	1.28979	-6.46564	1.19455
C	1.44381	-5.45425	-0.73854
H	1.72233	-6.41285	-1.23559
H	2.40085	-4.93761	-0.50307
C	0.62210	-4.60490	-1.73936
H	0.45346	-3.57611	-1.34380
H	1.16177	-4.50514	-2.70611
C	-3.94516	-6.02854	-0.18742
H	-4.78351	-6.32537	-0.84889
H	-3.67267	-6.88407	0.46265
C	-3.87961	-4.80202	1.98546
C	-4.98586	-3.69957	0.25129
H	-4.17725	-3.06629	-0.20156
C	-5.65716	-1.58223	1.43272
H	-4.74385	-1.06884	1.05441
H	-5.91986	-1.11490	2.40688
C	-6.82527	-1.44702	0.42484
H	-5.32826	-3.02050	4.22332
H	-7.01470	-0.37199	0.20964
H	-7.75222	-1.83693	0.90632
C	-6.58727	-2.20738	-0.90141
H	-3.97383	-4.21298	4.41208
H	-7.50241	-2.15354	-1.53100
H	-5.78435	-1.68978	-1.47801
C	-6.17407	-3.68788	-0.70862
H	-7.01394	-4.28035	-0.27874
H	-5.89518	-4.14042	-1.68460
H	-0.71889	-5.24845	-4.57110
N	-1.75253	-4.76073	-2.80593
N	-2.82183	-5.71882	-1.05850
N	-4.39429	-4.96443	0.69751
N	-4.30785	-3.55969	2.46863
O	-4.06622	-5.13125	-2.96251
O	-3.18417	-5.61818	2.60127
C	-2.48636	0.19994	-0.61751
O	-1.48448	1.11317	-0.15917
H	-2.36984	-0.03960	-1.70051
H	-3.51217	0.59567	-0.44181
H	-2.35953	-0.73308	-0.03174
H	-1.59230	1.97306	-0.61983
H	0.51372	-1.09495	1.72348
C	1.08832	-0.99543	0.76637
H	0.62897	-1.69380	0.01889
H	2.12466	-1.34728	0.95929
O	1.15585	0.33491	0.30145
H	0.22719	0.64713	0.11562

(2MeOH)@cycHC[8] d

196

C	0.47454	-3.07780	-5.27994
C	-0.71890	-1.12082	-5.72651
H	-0.56407	-0.51547	-4.79453
C	-1.69941	-2.25609	-5.39807
H	-2.05062	-2.69813	-6.36918
C	-2.93029	-1.70782	-4.67946
H	-2.62164	-1.20686	-3.73278
H	-3.63270	-2.52602	-4.40817
C	-3.60177	-0.70156	-5.64626
H	-4.03072	-1.26993	-6.50476
H	-4.46409	-0.21190	-5.14136
C	-2.63381	0.37724	-6.18841
H	-2.36712	1.07468	-5.35998
H	-3.15324	0.99476	-6.95464
C	-1.32144	-0.19426	-6.78165
H	-1.52575	-0.76604	-7.71657
H	-0.61887	0.63123	-7.02535
C	-5.16352	-2.62095	1.29874
C	-3.78013	-0.97764	4.84953
C	-2.02560	-2.49919	4.66946
H	-2.20425	-3.13657	5.57830
C	-1.46751	-1.14860	5.14009
H	-1.06954	-0.60840	4.24450
C	-0.30078	-1.36198	6.10208
H	-0.64662	-1.92111	7.00295
H	0.11453	-0.38650	6.43550
C	0.78006	-2.16022	5.32920

H	1.18696	-1.50683	4.52452
H	1.62417	-2.40155	6.01325
C	0.24597	-3.45777	4.67791
H	-0.01002	-4.19721	5.47336
H	1.05136	-3.92478	4.06810
C	-1.00432	-3.22252	3.79485
H	-0.73859	-2.59007	2.91829
H	-1.41232	-4.18859	3.42365
C	-2.76717	0.78253	6.28154
H	-3.78963	0.87379	6.70001
H	-2.02943	0.77248	7.10921
C	-1.18788	2.39129	5.23547
C	-3.39072	2.53656	4.47991
H	-3.40378	1.85988	3.58566
C	-2.63169	3.81624	4.09938
H	-2.81304	4.57129	4.91099
C	-3.18177	4.40302	2.80161
H	-3.07862	3.66209	1.97757
H	-2.61710	5.30912	2.49271
C	-4.67342	4.73208	3.05255
H	-5.13919	5.10400	2.11313
H	-4.73469	5.57441	3.78173
C	-5.48206	3.53002	3.59571
H	-6.51783	3.85427	3.83983
H	-5.58105	2.76417	2.79083
C	-4.84215	2.85489	4.83369
H	-4.87798	3.53275	5.71777
H	-5.39030	1.92138	5.08456
H	-2.21197	-4.78441	-4.92441
C	-0.11184	4.26116	4.05187
H	-0.50146	5.29728	3.95667
C	0.74233	4.98616	1.85123
C	2.15093	3.59423	3.07753
H	2.63816	4.38606	3.70636
C	2.70582	3.73295	1.65319
H	2.38878	2.83654	1.06317
C	4.23321	3.76736	1.67903
H	4.58294	4.64431	2.27229
H	4.63946	3.86357	0.64854
C	4.70837	2.44394	2.32732
H	4.46941	1.60636	1.63058
H	5.81618	2.45517	2.42954
C	4.05736	2.15696	3.70042
H	4.44431	2.89057	4.44714
H	4.37153	1.15303	4.06133
C	2.51162	2.23801	3.67611
H	2.08517	1.41042	3.06623
H	2.09004	2.12783	4.69820
C	2.20311	5.63228	-0.05264
H	1.60727	6.56486	0.01361
H	3.28246	5.88151	-0.10317
C	2.81562	4.13744	-1.94275
C	0.53039	4.55353	-1.69903
H	0.20428	3.68996	-1.06179
C	0.80648	4.02436	-3.11407
H	0.89993	4.90909	-3.80057
C	-0.36868	3.18690	-3.61280
H	-0.52905	2.31766	-2.93455
H	-0.16675	2.78473	-4.63004
C	-1.60721	4.11591	-3.62940
H	-2.51219	3.53361	-3.91355
H	-1.46642	4.87667	-4.43271
C	-1.85541	4.84186	-2.28608
H	-2.69400	5.56425	-2.39964
H	-2.19039	4.09515	-1.52773
C	-0.60796	5.57213	-1.72935
H	-0.33031	6.43208	-2.38215
H	-0.81495	5.96635	-0.71086
C	2.87071	2.86800	-4.06560
H	2.54006	3.35325	-5.01194
H	3.94643	3.08816	-3.88493
C	2.10967	0.93905	-5.41567
C	3.63835	0.45320	-3.72859
H	4.61395	0.59403	-4.26863
C	3.00911	-0.85625	-4.22695
H	2.17396	-1.12461	-3.52750
C	4.03205	-1.98942	-4.17813
H	4.89871	-1.74692	-4.83597
H	3.58090	-2.94047	-4.53422
C	4.48185	-2.13098	-2.70241
H	3.62462	-2.53396	-2.11377
H	5.29059	-2.89175	-2.62828
C	4.95572	-0.80473	-2.06017
H	5.92920	-0.50640	-2.51586
H	5.15924	-0.96526	-0.97798
C	3.95282	0.36224	-2.23744
H	3.01693	0.17429	-1.66349
H	4.38659	1.31395	-1.85928
C	1.75752	-1.29506	-6.44582
H	2.42964	-2.14141	-6.69275
H	1.57042	-0.69099	-7.35674
N	0.50264	-1.88065	-6.00426
N	-0.81566	-3.22700	-4.74950
N	-3.33355	-2.09994	4.13754
N	-2.70126	-0.48934	5.59079
N	-2.49144	1.97471	5.49074
N	-1.23739	3.35721	4.20436
N	0.73720	3.99370	2.88024
N	1.95033	4.89963	1.18070
N	1.88030	4.95048	-1.28905
N	2.13609	3.43497	-2.94726
N	2.68592	1.44005	-4.24017
N	2.43351	-0.41920	-5.50232
O	1.40800	-3.87066	-5.14319
O	-4.92346	-0.51419	4.83009
O	-0.16864	2.01764	5.81383
O	-0.17557	5.77483	1.63214
O	4.01923	4.06369	-1.69275
O	1.44672	1.58736	-6.22779
H	-6.21431	-2.93082	1.54850
H	0.52555	4.17531	4.95841
C	-4.29287	-3.07627	3.65408
C	-1.28728	-4.54354	-4.35313
C	-2.92746	-4.97990	-2.55836
C	-0.69412	-5.33600	-2.00558
H	-0.54501	-6.39623	-2.34586
C	-1.53773	-5.36951	-0.72280
H	-1.47311	-4.35952	-0.23871
C	-0.95910	-6.38741	0.25955
H	-0.96421	-7.40213	-0.20149
H	-1.56571	-6.42838	1.18990
C	0.48771	-5.93492	0.57902
H	0.43611	-4.99699	1.18011
H	0.98096	-6.69068	1.23002
C	1.35788	-5.68299	-0.67561
H	1.58207	-6.65993	-1.16567
H	2.34092	-5.26049	-0.37021
C	0.68859	-4.75426	-1.71948
H	0.58801	-3.71734	-1.32322
H	1.29826	-4.69812	-2.64784
C	-4.08983	-5.72297	-0.48921
H	-4.90944	-5.89250	-1.21646
H	-3.97193	-6.61686	0.15696
C	-3.98896	-4.58526	1.71866
C	-4.78618	-3.27405	-0.03979
H	-3.85227	-2.77694	-0.41427
C	-5.15757	-1.09885	1.17175
H	-4.14030	-0.74998	0.87986
H	-5.40951	-0.61620	2.14227
C	-6.20373	-0.73326	0.08962
H	-5.30162	-2.70675	3.94595
H	-6.18450	0.36319	-0.10059
H	-7.21946	-0.95742	0.49250
C	-6.01123	-1.49589	-1.24382
H	-4.09878	-4.06094	4.13707
H	-6.85449	-1.26332	-1.93136
H	-5.08955	-1.12066	-1.74826
C	-5.88232	-3.03058	-1.07591
H	-6.84262	-3.47175	-0.72201
H	-5.62272	-3.50647	-2.04661
H	-0.48351	-5.26760	-4.61419
N	-1.61065	-4.68242	-2.94312
N	-2.88264	-5.53998	-1.27814
N	-4.45373	-4.63087	0.39824
N	-4.25389	-3.30264	2.21949
O	-3.93721	-4.79303	-3.24034
O	-3.45589	-5.50732	2.33685
C	-1.40891	1.52462	0.82659
O	-0.06231	1.74128	1.22458
H	-1.83584	2.41554	0.30665
H	-2.07095	1.26125	1.68571
H	-1.41134	0.67285	0.11392
H	0.00040	2.48516	1.89495
H	1.34449	-1.17793	0.08387
C	1.83831	-1.09295	1.08656
H	2.74948	-0.45198	0.95952
H	2.18415	-2.10962	1.37674
O	0.96830	-0.62271	2.09164
H	0.60782	0.26915	1.80668

(2MeOH)@cycHC[8] e

196

C	0.39634	-3.19859	-5.28329
C	-0.97422	-1.42543	-5.93626
H	-0.99863	-0.77141	-5.02877
C	-1.83622	-2.66132	-5.64459
H	-2.02698	-3.19263	-6.61424
C	-3.19050	-2.23755	-5.08115
H	-3.04847	-1.65759	-4.14011
H	-3.81619	-3.12070	-4.82851
C	-3.87324	-1.36896	-6.16620
H	-4.12953	-2.02371	-7.03180
H	-4.83729	-0.97234	-5.77803
C	-2.99373	-0.19571	-6.66062
H	-2.90275	0.55888	-5.84431
H	-3.50100	0.31982	-7.50574
C	-1.56697	-0.62085	-7.09079
H	-1.59616	-1.24098	-8.01577
H	-0.95154	0.28132	-7.29669
C	-4.87361	-2.56624	1.41083
C	-3.76052	-0.91701	5.03777
C	-1.92639	-2.33943	4.92283
H	-2.11288	-2.99364	5.81556
C	-1.46132	-0.96647	5.42890
H	-1.04840	-0.39586	4.55693
C	-0.33395	-1.13136	6.44617
H	-0.69098	-1.72707	7.31749
H	0.00650	-0.14128	6.81882
C	0.82614	-1.85656	5.71835
H	1.25429	-1.15978	4.95921
H	1.64470	-2.07008	6.44040
C	0.39982	-3.16758	5.01562
H	0.15903	-3.93248	5.78985
H	1.25618	-3.57265	4.43206
C	-0.83178	-3.00286	4.09107
H	-0.58614	-2.36986	3.20793
H	-1.16722	-3.99238	3.71029
C	-2.88381	0.94330	6.43987

H	-3.95370	1.05040	6.70925
H	-2.27024	0.97156	7.36209
C	-1.16548	2.55970	5.64490
C	-3.12145	2.47419	4.37404
H	-2.87486	1.71124	3.59114
C	-2.34210	3.74993	4.02593
H	-2.76049	4.59484	4.63457
C	-2.54093	4.10019	2.55230
H	-2.19003	3.26407	1.90354
H	-1.95789	5.00651	2.27847
C	-4.05748	4.34992	2.35794
H	-4.27116	4.53974	1.28246
H	-4.33858	5.28270	2.90145
C	-4.94379	3.18655	2.86211
H	-6.01633	3.47310	2.78741
H	-4.80653	2.30869	2.18794
C	-4.62420	2.74116	4.31104
H	-4.89936	3.53694	5.04103
H	-5.19722	1.82353	4.56560
H	-2.15343	-5.15867	-4.87066
C	0.09083	4.37172	4.51521
H	-0.31212	5.40742	4.46920
C	1.07287	5.18244	2.38993
C	2.24953	3.48133	3.44873
H	2.94433	4.07967	4.09525
C	2.73691	3.59489	1.99755
H	2.19935	2.82020	1.39017
C	4.23274	3.29511	1.91770
H	4.80019	4.02778	2.53638
H	4.59488	3.37576	0.86997
C	4.43595	1.85318	2.44570
H	3.98791	1.14302	1.71095
H	5.52262	1.61782	2.48151
C	3.80514	1.60356	3.83587
H	4.38253	2.16785	4.60504
H	3.90098	0.52711	4.09999
C	2.32030	2.03280	3.92369
H	1.68247	1.38771	3.27743
H	1.94199	1.93317	4.96460
C	2.44043	5.56142	0.35002
H	1.98746	6.57073	0.42066
H	3.53066	5.66140	0.17659
C	2.65517	3.98394	-1.56473
C	0.47552	4.68974	-1.11693
H	0.07332	3.87582	-0.45861
C	0.55690	4.14933	-2.55162
H	0.73633	5.01855	-3.23762
C	-0.76605	3.50643	-2.96008
H	-1.00426	2.66524	-2.27175
H	-0.70833	3.09269	-3.99122
C	-1.84764	4.61113	-2.87860
H	-2.84784	4.17238	-3.09012
H	-1.65381	5.35606	-3.68505
C	-1.87833	5.33861	-1.51392
H	-2.60409	6.18080	-1.55646
H	-2.25869	4.63500	-0.73701
C	-0.49591	5.86739	-1.05719
H	-0.14297	6.68421	-1.72829
H	-0.55841	6.27409	-0.02497
C	2.36911	2.77120	-3.68955
H	1.90026	3.23363	-4.58454
H	3.45988	2.98703	-3.68851
C	1.61354	0.84534	-5.02750
C	3.31070	0.42401	-3.49258
H	4.18466	0.76485	-4.10642
C	2.82414	-0.90973	-4.07260
H	2.10640	-1.37233	-3.34797
C	3.99680	-1.87502	-4.23156
H	4.75509	-1.44594	-4.92636
H	3.64813	-2.84327	-4.64867
C	4.59952	-2.08923	-2.82078
H	3.86224	-2.65818	-2.20669
H	5.50449	-2.73177	-2.89552
C	4.95495	-0.77365	-2.09021
H	5.82613	-0.29927	-2.59939
H	5.28500	-1.00109	-1.05243
C	3.79112	0.24736	-2.05472
H	2.95297	-0.11768	-1.41834
H	4.13595	1.21735	-1.63451
C	1.57031	-1.27979	-6.32610
H	2.36144	-2.00765	-6.59202
H	1.38155	-0.61769	-7.19394
N	0.35809	-2.03109	-6.05125
N	-0.90276	-3.47899	-4.85656
N	-3.22698	-2.00270	4.33229
N	-2.74230	-0.36481	5.81672
N	-2.47991	2.08494	5.63507
N	-1.01322	3.42779	4.56095
N	0.97172	4.19948	3.37434
N	2.21812	4.91807	1.63326
N	1.90047	4.87923	-0.81640
N	1.79891	3.36107	-2.49268
N	2.17534	1.31936	-3.80494
N	2.07487	-0.44104	-5.24498
O	1.40444	-3.87122	-5.03867
O	-4.93430	-0.53164	4.98996
O	-0.29456	2.27759	6.47687
O	0.29718	6.13262	2.22774
O	3.86940	3.77914	-1.46034
O	0.85774	1.49033	-5.75674
H	-5.92967	-2.85782	1.65384
H	0.67194	4.24298	5.45355
C	-4.11374	-3.02291	3.80025
C	-1.24680	-4.79467	-4.34199
C	-2.80458	-5.17809	-2.46074
C	-0.54175	-5.34483	-1.94728
H	-0.33311	-6.41742	-2.20062
C	-1.34892	-5.31340	-0.64184
H	-1.33144	-4.26315	-0.24714
C	-0.68617	-6.20978	0.40390
H	-0.63916	-7.25736	0.02760
H	-1.26742	-6.21254	1.35129
C	0.73684	-5.64643	0.64558
H	0.64297	-4.66623	1.16990
H	1.29124	-6.31674	1.33912
C	1.55815	-5.44715	-0.65051
H	1.83199	-6.44567	-1.06466
H	2.51695	-4.93668	-0.40885
C	0.80467	-4.65272	-1.74592
H	0.64435	-3.59613	-1.42955
H	1.38740	-4.62911	-2.69168
C	-3.86729	-5.74433	-0.28261
H	-4.72127	-5.94197	-0.96170
H	-3.73087	-6.60977	0.39744
C	-3.74679	-4.54154	1.89537
C	-4.48569	-3.25516	0.09411
H	-3.54079	-2.78005	-0.28072
C	-4.84772	-1.04778	1.24531
H	-3.83079	-0.70068	0.95171
H	-5.11259	-0.54100	2.19956
C	-5.88367	-0.70150	0.14636
H	-5.15255	-2.70347	4.03976
H	-5.85036	0.38924	-0.06975
H	-6.90476	-0.91100	0.54297
C	-5.68096	-1.49508	-1.16644
H	-3.90374	-3.99620	4.29724
H	-6.51507	-1.27360	-1.86812
H	-4.75018	-1.13806	-1.66729
C	-5.56922	-3.02628	-0.95855
H	-6.53700	-3.44820	-0.60276
H	-5.30952	-3.53023	-1.91513
H	-0.39533	-5.47563	-4.55932
N	-1.52540	-4.83751	-2.91319
N	-2.69724	-5.60341	-1.13450
N	-4.17411	-4.60647	0.56903
N	-3.99100	-3.24598	2.36808
O	-3.84476	-5.13190	-3.12767
O	-3.25549	-5.46911	2.54940
C	-0.56256	-0.39448	-2.19831
O	0.30942	0.50471	-1.52150
H	-0.01510	-1.24483	-2.66858
H	-1.16716	0.12117	-2.98186
H	-1.26123	-0.80426	-1.43999
H	0.89251	0.95942	-2.19014
H	-0.20163	-0.41007	1.33800
C	-0.89940	0.41766	1.61625
H	-0.36260	1.09708	2.32627
H	-1.75469	-0.03689	2.16637
O	-1.39840	1.10599	0.48423
H	-0.68025	1.11104	-0.21015

(2MeOH)@cycHC[8] f

196

C	0.41444	-2.98219	-5.34107
C	-0.93384	-1.09422	-5.58193
H	-0.69863	-0.50750	-4.65576
C	-1.80009	-2.29445	-5.17483
H	-2.23372	-2.74310	-6.10724
C	-2.96594	-1.84046	-4.29993
H	-2.58156	-1.35301	-3.37505
H	-3.58929	-2.70661	-3.98685
C	-3.80222	-0.84799	-5.14518
H	-4.29500	-1.41465	-5.96946
H	-4.62213	-0.42378	-4.52406
C	-2.96580	0.30244	-5.75389
H	-2.63578	0.98409	-4.93496
H	-3.60686	0.91314	-6.42730
C	-1.70951	-0.17661	-6.52411
H	-1.99620	-0.73058	-7.44742
H	-1.08835	0.69599	-6.81927
C	-5.06531	-2.82034	1.52418
C	-3.92313	-0.87480	5.03623
C	-2.16589	-2.38050	5.27295
H	-2.50809	-2.94787	6.17835
C	-1.69848	-0.99308	5.73239
H	-1.14000	-0.51597	4.88618
C	-0.72703	-1.12292	6.90393
H	-1.22662	-1.62877	7.76164
H	-0.38434	-0.12088	7.23961
C	0.47856	-1.95425	6.39845
H	1.04032	-1.34043	5.65533
H	1.18202	-2.14341	7.23922
C	0.07926	-3.29794	5.74375
H	-0.30203	-3.98693	6.53366
H	0.98210	-3.78601	5.31436
C	-1.00851	-3.15745	4.65016
H	-0.62558	-2.61689	3.75530
H	-1.34620	-4.15843	4.30453
C	-3.13529	1.05381	6.39794
H	-4.21514	1.20809	6.59315
H	-2.57848	1.13743	7.35200
C	-1.31843	2.50337	5.50601
C	-3.29155	2.50086	4.26099
H	-3.10714	1.68449	3.51541
C	-2.43366	3.70854	3.85957
H	-2.78625	4.59772	4.44651
C	-2.62630	4.03225	2.38020
H	-2.32440	3.16072	1.75489

H	-1.99434	4.89544	2.07608
C	-4.12676	4.36299	2.18463
H	-4.33656	4.52946	1.10484
H	-4.34564	5.32850	2.69822
C	-5.07654	3.27240	2.73420
H	-6.12993	3.62093	2.65429
H	-4.99871	2.36468	2.09077
C	-4.77445	2.85944	4.19665
H	-4.99593	3.69489	4.89983
H	-5.40071	1.98729	4.48313
H	-2.03177	-4.91377	-4.69880
C	0.04478	4.17000	4.28174
H	-0.27318	5.23548	4.24329
C	1.12750	4.95960	2.20174
C	1.99902	3.00734	3.11859
H	2.76130	3.41793	3.83290
C	2.54078	3.17284	1.69152
H	1.91218	2.55224	1.00239
C	3.97169	2.64477	1.61069
H	4.62651	3.19933	2.32199
H	4.39020	2.77763	0.58910
C	3.90770	1.14001	1.97357
H	3.35857	0.61066	1.16045
H	4.93509	0.71376	1.99350
C	3.20506	0.85981	3.32306
H	3.85012	1.23128	4.15389
H	3.10792	-0.23860	3.47212
C	1.81281	1.52780	3.44653
H	1.08638	1.07112	2.73723
H	1.40839	1.39507	4.47448
C	2.63576	5.32482	0.25895
H	2.25839	6.35926	0.39095
H	3.74153	5.34590	0.17807
C	2.92573	3.95051	-1.79226
C	0.73385	4.63440	-1.36276
H	0.30684	3.78523	-0.77041
C	0.85451	4.18014	-2.82343
H	1.06702	5.08427	-3.45158
C	-0.46216	3.57825	-3.30581
H	-0.73030	2.70448	-2.67017
H	-0.37708	3.21746	-4.35391
C	-1.53477	4.68874	-3.19033
H	-2.53250	4.27478	-3.45639
H	-1.31037	5.47708	-3.94606
C	-1.59930	5.33681	-1.78699
H	-2.31391	6.18930	-1.80201
H	-2.01193	4.59467	-1.06368
C	-0.22483	5.81983	-1.26079
H	0.15736	6.66946	-1.87220
H	-0.31269	6.16990	-0.20944
C	2.70669	2.90849	-4.02435
H	2.24690	3.45636	-4.87475
H	3.78981	3.15103	-3.96878
C	1.83520	1.05885	-5.40940
C	3.65137	0.53678	-4.05302
H	4.49844	0.82186	-4.73109
C	3.03637	-0.76421	-4.58495
H	2.36000	-1.17878	-3.79309
C	4.12908	-1.80102	-4.83878
H	4.85224	-1.41837	-5.59504
H	3.68891	-2.74570	-5.22275
C	4.83040	-2.05633	-3.48112
H	4.11328	-2.58653	-2.81085
H	5.68810	-2.74944	-3.62825
C	5.31886	-0.76736	-2.77851
H	6.17698	-0.34495	-3.35190
H	5.71254	-1.01981	-1.76902
C	4.22797	0.32495	-2.65547
H	3.41984	0.02036	-1.95264
H	4.66326	1.26793	-2.25966

C	1.44368	-1.09456	-6.59527
H	2.11443	-1.87786	-7.00103
H	1.10160	-0.44544	-7.42565
N	0.28818	-1.77310	-6.02986
N	-0.78318	-3.21696	-4.66018
N	-3.35676	-2.03407	4.48115
N	-2.98411	-0.30385	5.89324
N	-2.66317	2.12420	5.53352
N	-1.12244	3.31203	4.38338
N	0.85596	3.92662	3.10185
N	2.24426	4.59135	1.44828
N	2.14897	4.78720	-1.00464
N	2.08790	3.36966	-2.77829
N	2.55148	1.48723	-4.28000
N	2.21124	-0.25442	-5.68623
O	1.40494	-3.72220	-5.34354
O	-5.06278	-0.45259	4.81371
O	-0.45578	2.19024	6.33470
O	0.49808	6.02044	2.10254
O	4.13667	3.74403	-1.67698
O	1.02906	1.74047	-6.05150
H	-6.03433	-3.30165	1.81680
H	0.65255	3.99907	5.19765
C	-4.21973	-3.05639	3.91643
C	-1.11516	-4.54843	-4.18477
C	-2.58719	-5.12011	-2.28214
C	-0.31858	-4.95951	-1.79364
H	0.03072	-6.00264	-2.01746
C	-1.11034	-4.99106	-0.47844
H	-1.24663	-3.93676	-0.12400
C	-0.31649	-5.73843	0.59159
H	-0.11058	-6.78200	0.25797
H	-0.88359	-5.79078	1.54600
C	1.00191	-4.95064	0.79382
H	0.75081	-3.96762	1.25611
H	1.65242	-5.48708	1.51978
C	1.78348	-4.70643	-0.51872
H	2.19328	-5.67693	-0.88506
H	2.66189	-4.05463	-0.31368
C	0.92190	-4.08206	-1.64447
H	0.62087	-3.04175	-1.38624
H	1.49742	-4.03993	-2.59559
C	-3.52989	-5.82311	-0.09152
H	-4.34016	-6.18141	-0.75818
H	-3.24468	-6.63412	0.60868
C	-3.61847	-4.56286	2.04872
C	-4.58510	-3.47466	0.22162
H	-3.74593	-2.85664	-0.18824
C	-5.33218	-1.33374	1.30872
H	-4.39988	-0.83023	0.96684
H	-5.65148	-0.84378	2.25450
C	-6.43835	-1.22656	0.23080
H	-5.27183	-2.74821	4.09707
H	-6.61504	-0.15701	-0.01928
H	-7.39207	-1.60934	0.66334
C	-6.11632	-2.01490	-1.06017
H	-4.02353	-4.02201	4.43048
H	-6.99028	-1.97816	-1.74731
H	-5.27725	-1.50931	-1.59348
C	-5.71633	-3.48925	-0.80516
H	-6.58047	-4.07028	-0.40817
H	-5.38608	-3.97116	-1.75095
H	-0.25813	-5.20825	-4.44635
N	-1.37194	-4.62125	-2.75723
N	-2.40544	-5.49972	-0.95053
N	-4.02941	-4.73239	0.73490
N	-4.03505	-3.27649	2.47873
O	-3.63406	-5.22320	-2.93363
O	-3.00735	-5.38073	2.74120
C	0.23145	0.07155	-1.72221

O	1.02200	1.05487	-1.07739
H	0.82727	-0.58474	-2.40314
H	-0.60938	0.50907	-2.31725
H	-0.21461	-0.57361	-0.93625
H	1.44436	1.63658	-1.76239
H	-2.48865	0.23258	1.47758
C	-1.55470	-0.33956	1.70683
H	-1.29954	-0.16026	2.78051
H	-0.73804	0.09111	1.09001
O	-1.65802	-1.71657	1.39008
H	-2.39765	-2.11781	1.91854

(2MeOH)@cycHC[8] g

196

C	0.42507	-2.99021	-5.18727
C	-0.81210	-1.03079	-5.45822
H	-0.55520	-0.44328	-4.53724
C	-1.74800	-2.17705	-5.04902
H	-2.19780	-2.60637	-5.98276
C	-2.89510	-1.64963	-4.18998
H	-2.49174	-1.17541	-3.26604
H	-3.56992	-2.47621	-3.87649
C	-3.66699	-0.62036	-5.05279
H	-4.18553	-1.16846	-5.87372
H	-4.46549	-0.14272	-4.44289
C	-2.76252	0.47266	-5.66962
H	-2.40196	1.14726	-4.85747
H	-3.36345	1.10816	-6.35689
C	-1.52851	-0.08638	-6.42184
H	-1.83895	-0.64028	-7.33754
H	-0.85703	0.74384	-6.72994
C	-5.21518	-2.86402	1.59454
C	-3.99883	-1.09853	5.08479
C	-2.33804	-2.70568	5.33856
H	-2.68666	-3.22284	6.27110
C	-1.76906	-1.33636	5.73537
H	-1.20475	-0.92997	4.85875
C	-0.77699	-1.48430	6.88701
H	-1.28702	-1.91357	7.77943
H	-0.35357	-0.49656	7.16963
C	0.35551	-2.41892	6.39210
H	0.94340	-1.87588	5.61521
H	1.06125	-2.62562	7.22661
C	-0.15081	-3.75480	5.79770
H	-0.56615	-4.38584	6.61818
H	0.70683	-4.32115	5.37186
C	-1.24599	-3.57339	4.71740
H	-0.83048	-3.07401	3.81215
H	-1.65031	-4.55669	4.39359
C	-3.03136	0.84157	6.28826
H	-4.07351	1.09224	6.56692
H	-2.37942	0.97346	7.17392
C	-1.25984	2.10485	5.07681
C	-3.39805	2.28987	4.15104
H	-3.41904	1.50462	3.35292
C	-2.52470	3.45424	3.66638
H	-2.67565	4.31089	4.37292
C	-2.94841	3.91465	2.27468
H	-2.83941	3.07671	1.55008
H	-2.30309	4.74920	1.92281
C	-4.42439	4.36986	2.38304
H	-4.80682	4.64236	1.37461
H	-4.46594	5.29988	2.99663
C	-5.34887	3.30435	3.01702
H	-6.37017	3.72477	3.14891
H	-5.45285	2.44728	2.31066
C	-4.83352	2.76096	4.37306
H	-4.85903	3.55563	5.15395

H	-5.47107	1.91694	4.71285
H	-2.14060	-4.76996	-4.64992
C	-0.01770	3.84519	3.83824
H	-0.40425	4.88673	3.86801
C	0.93849	4.81898	1.79064
C	2.10298	3.02409	2.70682
H	2.73226	3.54618	3.47562
C	2.67852	3.33513	1.31792
H	2.22066	2.63032	0.57984
C	4.18966	3.10838	1.32349
H	4.67577	3.76861	2.07904
H	4.62664	3.34198	0.32842
C	4.41617	1.61477	1.66871
H	4.05585	1.00160	0.80985
H	5.50615	1.41222	1.76390
C	3.69079	1.15159	2.95500
H	4.18499	1.61596	3.84045
H	3.80823	0.05121	3.07246
C	2.18795	1.52398	2.98097
H	1.63116	0.97137	2.19219
H	1.73963	1.26710	3.96659
C	2.44426	5.51395	-0.06455
H	1.93405	6.48362	0.10727
H	3.54018	5.67919	-0.09923
C	2.93852	4.22461	-2.13636
C	0.68489	4.71516	-1.78544
H	0.33484	3.79879	-1.24422
C	0.90346	4.33305	-3.25610
H	1.03718	5.27799	-3.84791
C	-0.32980	3.62253	-3.80838
H	-0.53482	2.70191	-3.21771
H	-0.17591	3.31230	-4.86481
C	-1.50940	4.61958	-3.69701
H	-2.45172	4.12389	-4.02028
H	-1.33469	5.45689	-4.41266
C	-1.69068	5.20055	-2.27467
H	-2.48681	5.97811	-2.28823
H	-2.05294	4.39278	-1.59650
C	-0.39063	5.79344	-1.67625
H	-0.07628	6.70398	-2.23782
H	-0.55222	6.08426	-0.61561
C	2.86459	3.07218	-4.33064
H	2.47424	3.53993	-5.26030
H	3.95136	3.28735	-4.23667
C	2.02393	1.09526	-5.57337
C	3.65352	0.65904	-3.97304
H	4.58640	0.77446	-4.58629
C	2.98461	-0.66259	-4.37464
H	2.20151	-0.89976	-3.60837
C	4.00577	-1.79853	-4.35928
H	4.82384	-1.58313	-5.08420
H	3.52917	-2.75940	-4.65057
C	4.55881	-1.89076	-2.91508
H	3.74398	-2.26258	-2.24991
H	5.36774	-2.65360	-2.87212
C	5.08428	-0.54457	-2.36183
H	6.02135	-0.27073	-2.90171
H	5.36576	-0.66705	-1.29242
C	4.07494	0.62150	-2.50604
H	3.18664	0.46983	-1.85252
H	4.54010	1.58414	-2.20222
C	1.56601	-1.18274	-6.46940
H	2.22334	-2.02306	-6.77040
H	1.27326	-0.60762	-7.37094
N	0.37593	-1.77991	-5.88094
N	-0.79352	-3.15539	-4.51745
N	-3.52670	-2.30898	4.57116
N	-3.00303	-0.55852	5.90402
N	-2.59276	1.79974	5.27907
N	-1.16311	2.92061	3.90578
N	0.80434	3.71307	2.65150
N	2.12603	4.67689	1.08316
N	2.06578	4.99807	-1.36857
N	2.20531	3.65957	-3.18075
N	2.67168	1.63547	-4.46035
N	2.31723	-0.27126	-5.62115
O	1.36930	-3.78869	-5.17762
O	-5.10660	-0.59648	4.86472
O	-0.30628	1.74964	5.77083
O	0.14270	5.76253	1.71165
O	4.15391	4.08714	-1.94500
O	1.32929	1.71948	-6.38442
H	-6.24610	-3.25064	1.80872
H	0.60586	3.65310	4.73783
C	-4.44501	-3.27728	3.99390
C	-1.21821	-4.48060	-4.09838
C	-2.77290	-5.06398	-2.26897
C	-0.51530	-5.05024	-1.71556
H	-0.21529	-6.09425	-1.99648
C	-1.34458	-5.12623	-0.42577
H	-1.43407	-4.08774	-0.00934
C	-0.62082	-5.97956	0.61448
H	-0.46854	-7.01021	0.21947
H	-1.21534	-6.05763	1.55036
C	0.73819	-5.29030	0.89437
H	0.54344	-4.33140	1.43079
H	1.33870	-5.91722	1.58982
C	1.56383	-4.99669	-0.38081
H	1.93227	-5.96017	-0.80413
H	2.46854	-4.40802	-0.11073
C	0.76546	-4.25400	-1.48082
H	0.51608	-3.21441	-1.16434
H	1.36525	-4.18247	-2.41446
C	-3.81264	-5.86181	-0.15032
H	-4.61051	-6.15055	-0.86389
H	-3.57540	-6.72489	0.50442
C	-3.91624	-4.73468	2.06759
C	-4.73950	-3.48761	0.27424
H	-3.84010	-2.91540	-0.07733
C	-5.31717	-1.34696	1.45153
H	-4.32416	-0.92375	1.17419
H	-5.62352	-0.87430	2.40998
C	-6.36157	-1.07154	0.34147
H	-5.47823	-2.92215	4.19955
H	-6.41711	0.02146	0.14140
H	-7.36616	-1.37195	0.72063
C	-6.07507	-1.82540	-0.97894
H	-4.28878	-4.25992	4.48858
H	-6.91887	-1.67050	-1.68704
H	-5.17598	-1.37748	-1.46414
C	-5.82437	-3.34297	-0.79240
H	-6.75329	-3.86009	-0.45876
H	-5.50235	-3.80313	-1.75166
H	-0.39587	-5.18237	-4.36219
N	-1.52085	-4.59483	-2.68126
N	-2.64984	-5.52872	-0.95781
N	-4.30176	-4.81020	0.72749
N	-4.29488	-3.48034	2.55964
O	-3.80204	-5.07816	-2.95416
O	-3.35709	-5.62761	2.71496
C	-1.18853	-0.24976	2.02096
O	-1.13166	1.11169	1.61314
H	-2.07409	-0.45851	2.66551
H	-0.26501	-0.57307	2.55938
H	-1.28725	-0.86934	1.10453
H	-1.00812	1.71142	2.40809
H	0.16454	-0.58621	-1.16293
C	0.07512	0.50036	-1.43192
H	-1.00269	0.70219	-1.65886
H	0.65382	0.66103	-2.36755
O	0.59653	1.35959	-0.44134
H	-0.03260	1.35347	0.34267

(2MeOH)@cycHC[8] h

196

C	0.41426	-2.95003	-4.91178
C	-0.78086	-1.02213	-5.46626
H	-0.72467	-0.40525	-4.53345
C	-1.75578	-2.17952	-5.21166
H	-1.97851	-2.66671	-6.19803
C	-3.07235	-1.65549	-4.64626
H	-2.87949	-1.09491	-3.70644
H	-3.77335	-2.48654	-4.41581
C	-3.66814	-0.70647	-5.71425
H	-3.96185	-1.30661	-6.60793
H	-4.60304	-0.24588	-5.32471
C	-2.68436	0.40659	-6.14567
H	-2.52995	1.09737	-5.28549
H	-3.13374	1.00859	-6.96676
C	-1.30097	-0.12951	-6.59147
H	-1.38804	-0.71670	-7.53516
H	-0.60103	0.71427	-6.77367
C	-5.13632	-2.79924	1.83177
C	-3.62845	-1.05171	5.23161
C	-1.75956	-2.39822	4.91946
H	-1.79003	-3.03130	5.84482
C	-1.29739	-0.99107	5.32333
H	-1.02423	-0.43253	4.38870
C	-0.04345	-1.08083	6.19136
H	-0.26212	-1.66599	7.11420
H	0.29785	-0.06893	6.49901
C	1.04621	-1.77993	5.33997
H	1.34741	-1.08819	4.51816
H	1.95657	-1.94156	5.95842
C	0.59239	-3.12579	4.72548
H	0.48614	-3.87973	5.54019
H	1.38425	-3.51034	4.04513
C	-0.75411	-3.03670	3.96464
H	-0.65497	-2.41481	3.04524
H	-1.09122	-4.04786	3.64773
C	-2.66381	0.89580	6.44370
H	-3.71161	0.98896	6.79396
H	-1.97951	0.97472	7.31225
C	-1.05561	2.54264	5.49624
C	-3.06329	2.32304	4.32593
H	-2.82940	1.52657	3.57133
C	-2.35314	3.61318	3.89165
H	-2.78138	4.46198	4.48730
C	-2.63184	3.90507	2.41927
H	-2.26780	3.06240	1.78722
H	-2.10053	4.82368	2.08639
C	-4.16450	4.08249	2.28208
H	-4.43332	4.21956	1.21129
H	-4.45810	5.02530	2.79992
C	-4.97889	2.90756	2.87462
H	-6.06370	3.15121	2.83926
H	-4.84160	2.00824	2.22892
C	-4.57692	2.53300	4.32353
H	-4.84757	3.34812	5.03334
H	-5.10479	1.60873	4.64397
H	-2.32927	-4.62628	-4.53547
C	0.05243	4.37815	4.26106
H	-0.41541	5.38431	4.18683
C	1.03337	5.25246	2.16549
C	2.21523	3.54157	3.20431
H	2.85586	4.12199	3.91958
C	2.77706	3.74767	1.79099
H	2.30993	2.98389	1.11460

C	4.28608	3.50711	1.78864
H	4.78512	4.22150	2.48303
H	4.70917	3.66215	0.77252
C	4.51034	2.04441	2.24727
H	4.13713	1.36420	1.44525
H	5.60076	1.84472	2.34001
C	3.80434	1.68953	3.57770
H	4.31654	2.22028	4.41398
H	3.91977	0.60180	3.78092
C	2.30369	2.07086	3.60302
H	1.72085	1.44709	2.88695
H	1.87490	1.90646	4.61600
C	2.51528	5.80089	0.23962
H	1.99885	6.77724	0.33402
H	3.60873	5.97027	0.16974
C	2.98627	4.33428	-1.71653
C	0.73534	4.85718	-1.38808
H	0.37062	4.00607	-0.75497
C	0.93844	4.33301	-2.81687
H	1.06773	5.22014	-3.49271
C	-0.29585	3.57216	-3.29239
H	-0.50932	2.69852	-2.63587
H	-0.14726	3.17814	-4.32173
C	-1.47061	4.58161	-3.26983
H	-2.41622	4.05994	-3.53701
H	-1.29982	5.34698	-4.06292
C	-1.64228	5.29932	-1.90997
H	-2.43479	6.07585	-1.99410
H	-2.00563	4.56256	-1.15524
C	-0.33873	5.94316	-1.37492
H	-0.02387	6.79451	-2.02171
H	-0.49363	6.33296	-0.34556
C	2.90312	3.05183	-3.83067
H	2.54709	3.52062	-4.77437
H	3.99417	3.23728	-3.71492
C	2.12289	1.08760	-5.12200
C	3.55343	0.63213	-3.34665
H	4.55532	0.72070	-3.84477
C	2.90594	-0.67857	-3.81351
H	2.03234	-0.89248	-3.14153
C	3.89467	-1.83425	-3.66996
H	4.79871	-1.64068	-4.29196
H	3.43661	-2.78712	-4.01092
C	4.26728	-1.92602	-2.16895
H	3.37062	-2.27741	-1.60561
H	5.04834	-2.70488	-2.02382
C	4.75141	-0.58725	-1.56264
H	5.75337	-0.33861	-1.98412
H	4.89531	-0.70577	-0.46571
C	3.79589	0.60028	-1.83951
H	2.82921	0.47156	-1.30128
H	4.24826	1.55355	-1.48879
C	1.74296	-1.18735	-6.06299
H	2.42296	-2.03675	-6.27455
H	1.57618	-0.61374	-6.99697
N	0.47883	-1.76204	-5.63156
N	-0.90119	-3.10158	-4.44083
N	-3.14196	-2.14084	4.50348
N	-2.54391	-0.42967	5.85560
N	-2.34733	2.01414	5.56826
N	-0.98877	3.37012	4.37164
N	0.92045	4.22315	3.10599
N	2.22337	5.06448	1.45977
N	2.11836	5.16732	-1.00706
N	2.24316	3.67206	-2.69603
N	2.65996	1.62519	-3.94957
N	2.39633	-0.28330	-5.13032
O	1.33840	-3.75252	-4.73417
O	-4.81397	-0.71031	5.32034
O	-0.13649	2.33088	6.29617
O	0.22745	6.17686	2.00388
O	4.20292	4.21522	-1.52391
O	1.53005	1.71733	-6.00551
H	-6.12859	-3.20173	2.16692
H	0.66155	4.32181	5.19013
C	-4.03430	-3.21244	4.09575
C	-1.36542	-4.41166	-4.02714
C	-2.91291	-4.87758	-2.15619
C	-0.68539	-5.42516	-1.79323
H	-0.70206	-6.43830	-2.27064
C	-1.44036	-5.52844	-0.46239
H	-1.26125	-4.59123	0.12361
C	-0.90872	-6.70283	0.35799
H	-1.06521	-7.65517	-0.19880
H	-1.44663	-6.77347	1.32733
C	0.59997	-6.44955	0.59859
H	0.70745	-5.57635	1.28460
H	1.04293	-7.31942	1.13184
C	1.39650	-6.17148	-0.69665
H	1.44573	-7.10617	-1.30271
H	2.44660	-5.90813	-0.44086
C	0.77610	-5.05102	-1.56667
H	0.84884	-4.07030	-1.04446
H	1.31381	-4.95066	-2.53364
C	-3.99834	-5.77902	-0.10530
H	-4.85067	-5.94241	-0.79437
H	-3.82394	-6.70161	0.48330
C	-3.78449	-4.66963	2.11244
C	-4.82076	-3.39468	0.45278
H	-3.96328	-2.82512	0.01059
C	-5.26947	-1.28149	1.73843
H	-4.32137	-0.83956	1.35548
H	-5.46974	-0.83578	2.73766
C	-6.44528	-0.99808	0.77067
H	-5.04697	-2.95740	4.47928
H	-6.53976	0.09787	0.60380
H	-7.39211	-1.32145	1.26331
C	-6.31118	-1.71886	-0.59212
H	-3.69747	-4.16999	4.55097
H	-7.23528	-1.55832	-1.19019
H	-5.48036	-1.24988	-1.16877
C	-6.02456	-3.23664	-0.47452
H	-6.90366	-3.77497	-0.05170
H	-5.80697	-3.66145	-1.47808
H	-0.60869	-5.16426	-4.33597
N	-1.58937	-4.55338	-2.57848
N	-2.82800	-5.52989	-0.93770
N	-4.34750	-4.73197	0.83628
N	-4.09739	-3.42200	2.65973
O	-3.93910	-4.62401	-2.78864
O	-3.13820	-5.56532	2.66918
C	-3.30938	1.13110	-1.68219
O	-2.08734	0.64443	-2.19636
H	-3.37152	1.06374	-0.56524
H	-4.20234	0.60155	-2.10351
H	-3.39832	2.20449	-1.95840
H	-1.98772	-0.31206	-1.91679
H	-0.32665	-2.39986	0.35837
C	-0.47763	-1.61788	-0.42283
H	0.45016	-1.54624	-1.03988
H	-0.62884	-0.64214	0.08429
O	-1.63674	-1.86541	-1.21325
H	-1.50469	-2.68196	-1.77579

(3MeOH)@cycHC[8]

202

C	0.50117	-3.07860	-4.87186
C	-0.68401	-1.15881	-5.47156
H	-0.67588	-0.54868	-4.53198
C	-1.66126	-2.32434	-5.26919
H	-1.84473	-2.80102	-6.26820
C	-2.99921	-1.80835	-4.74977
H	-2.84091	-1.27802	-3.78558
H	-3.70852	-2.64032	-4.55572
C	-3.55663	-0.84068	-5.82161
H	-3.82638	-1.43246	-6.72789
H	-4.50190	-0.38101	-5.45654
C	-2.55863	0.27226	-6.21970
H	-2.43632	0.97239	-5.36085
H	-2.98008	0.87119	-7.05741
C	-1.15924	-0.26188	-6.61329
H	-1.21062	-0.84633	-7.56103
H	-0.45265	0.58208	-6.76707
C	-5.06848	-2.64606	1.71244
C	-3.52565	-0.98261	5.16281
C	-1.68796	-2.34845	4.76256
H	-1.71261	-3.01029	5.66838
C	-1.19216	-0.96377	5.20189
H	-0.93147	-0.37736	4.28142
C	0.07869	-1.10409	6.03758
H	-0.12921	-1.71639	6.94522
H	0.44373	-0.10811	6.36799
C	1.13669	-1.79229	5.13915
H	1.42915	-1.07952	4.33259
H	2.05850	-1.98775	5.73059
C	0.64658	-3.11074	4.49443
H	0.54521	-3.88779	5.28785
H	1.41631	-3.48725	3.78478
C	-0.71475	-2.97530	3.76761
H	-0.62517	-2.32841	2.86493
H	-1.07611	-3.97146	3.43026
C	-2.50269	0.86877	6.48387
H	-3.52299	0.92440	6.91359
H	-1.76078	0.87376	7.30786
C	-0.96915	2.58004	5.52190
C	-3.11658	2.54408	4.60804
H	-3.00868	1.85135	3.73190
C	-2.42523	3.86878	4.25498
H	-2.72696	4.62811	5.02381
C	-2.90361	4.38059	2.89933
H	-2.67125	3.63341	2.10807
H	-2.38513	5.32302	2.61848
C	-4.43119	4.60280	3.01654
H	-4.84323	4.90736	2.02910
H	-4.61374	5.45732	3.70928
C	-5.19389	3.36045	3.53539
H	-6.26570	3.61628	3.68774
H	-5.17304	2.56753	2.75036
C	-4.60935	2.76985	4.84289
H	-4.75730	3.47217	5.69495
H	-5.11547	1.81219	5.09232
H	-2.25421	-4.77658	-4.63838
C	0.06626	4.48090	4.31490
H	-0.36692	5.50214	4.25615
C	0.92089	5.27756	2.13399
C	2.20167	3.64984	3.19037
H	2.84762	4.28773	3.85026
C	2.69643	3.81129	1.74639
H	2.23346	3.00404	1.12235
C	4.21469	3.64021	1.69835
H	4.70357	4.40993	2.33939
H	4.59392	3.76800	0.66123
C	4.52752	2.21460	2.21661
H	4.15395	1.48011	1.46460
H	5.62929	2.06948	2.27310
C	3.89000	1.89568	3.58953
H	4.40434	2.49406	4.37787
H	4.06733	0.82695	3.84302

C	2.37391	2.20385	3.64888
H	1.80738	1.52030	2.97638
H	1.97995	2.06155	4.67853
C	2.34103	5.82801	0.15637
H	1.77750	6.77909	0.24274
H	3.42566	6.05085	0.09071
C	2.89857	4.39380	-1.80451
C	0.62253	4.80151	-1.49548
H	0.30670	3.90372	-0.90391
C	0.87104	4.34190	-2.93950
H	0.97407	5.25583	-3.58415
C	-0.32065	3.54568	-3.46170
H	-0.48886	2.64330	-2.83373
H	-0.14312	3.19457	-4.50189
C	-1.54277	4.49643	-3.41676
H	-2.46010	3.94498	-3.72153
H	-1.39644	5.30067	-4.17560
C	-1.76625	5.14879	-2.03156
H	-2.59688	5.88645	-2.09567
H	-2.09905	4.36485	-1.31106
C	-0.50172	5.83330	-1.45579
H	-0.22432	6.72419	-2.06641
H	-0.68783	6.17733	-0.41500
C	2.88249	3.07382	-3.90555
H	2.54340	3.49571	-4.87700
H	3.96992	3.27363	-3.77652
C	2.17683	1.03603	-5.13224
C	3.49711	0.68088	-3.25068
H	4.52685	0.73226	-3.69513
C	2.86487	-0.65165	-3.67508
H	1.95080	-0.81619	-3.04529
C	3.83120	-1.80380	-3.40487
H	4.77169	-1.65473	-3.98348
H	3.38594	-2.77345	-3.71571
C	4.11518	-1.80595	-1.88193
H	3.18284	-2.11422	-1.35226
H	4.87751	-2.58041	-1.64375
C	4.57549	-0.43533	-1.33131
H	5.60286	-0.22095	-1.70876
H	4.65625	-0.48934	-0.22298
C	3.64963	0.74062	-1.73243
H	2.65355	0.66555	-1.24072
H	4.10001	1.70776	-1.41901
C	1.86028	-1.29488	-5.96636
H	2.56739	-2.13767	-6.10423
H	1.73431	-0.76472	-6.93234
N	0.58782	-1.88466	-5.58013
N	-0.83676	-3.24998	-4.47205
N	-3.07313	-2.05326	4.38229
N	-2.41778	-0.40316	5.78226
N	-2.25609	2.06604	5.69572
N	-1.01581	3.52443	4.49168
N	0.87607	4.28062	3.12064
N	2.08006	5.09217	1.38365
N	1.98628	5.17276	-1.09045
N	2.19785	3.73042	-2.81118
N	2.65007	1.63982	-3.96636
N	2.43954	-0.33507	-5.04193
O	1.42290	-3.87160	-4.64915
O	-4.70337	-0.62709	5.29120
O	0.03135	2.27212	6.18052
O	0.08739	6.17915	1.98236
O	4.11743	4.31916	-1.59757
O	1.63964	1.61380	-6.08507
H	-6.07872	-3.00366	2.04529
H	0.72485	4.41056	5.20829
C	-3.99066	-3.10449	3.97696
C	-1.30869	-4.56933	-4.09359
C	-2.92381	-4.91219	-2.24303
C	-0.78465	-5.73843	-1.88836
H	-0.93781	-6.72952	-2.38754
C	-1.54821	-5.76852	-0.55812
H	-1.24980	-4.87522	0.04703
C	-1.17724	-7.01880	0.23860
H	-1.46466	-7.93092	-0.33319
H	-1.71605	-7.03241	1.20966
C	0.35279	-6.97936	0.47656
H	0.58055	-6.14446	1.18003
H	0.67278	-7.91346	0.98897
C	1.17664	-6.78514	-0.81705
H	1.09556	-7.70375	-1.44397
H	2.25361	-6.67393	-0.56195
C	0.71344	-5.56976	-1.65657
H	0.91606	-4.62238	-1.10906
H	1.26383	-5.51574	-2.61951
C	-4.11982	-5.70072	-0.20694
H	-4.98091	-5.78703	-0.89943
H	-4.04160	-6.63087	0.39016
C	-3.80982	-4.57895	1.99707
C	-4.77900	-3.25656	0.33419
H	-3.89117	-2.73189	-0.10397
C	-5.13236	-1.12386	1.62270
H	-4.16141	-0.72435	1.24985
H	-5.31863	-0.67172	2.62189
C	-6.28799	-0.78341	0.64905
H	-4.99589	-2.82423	4.36303
H	-6.33094	0.31601	0.48394
H	-7.25139	-1.06326	1.13605
C	-6.18123	-1.50811	-0.71433
H	-3.67764	-4.07097	4.43156
H	-7.09271	-1.30184	-1.31761
H	-5.32550	-1.08010	-1.28606
C	-5.96961	-3.03818	-0.59766
H	-6.87578	-3.53276	-0.17901
H	-5.76951	-3.47197	-1.60101
H	-0.54161	-5.31542	-4.38901
N	-1.56645	-4.73519	-2.65113
N	-2.92395	-5.58226	-1.03241
N	-4.37483	-4.61513	0.72100
N	-4.05961	-3.31458	2.54112
O	-3.90839	-4.53528	-2.87931
O	-3.20908	-5.50443	2.55500
C	-2.96862	0.86403	-1.83404
O	-1.63264	0.42469	-2.03939
H	-3.35061	0.61038	-0.81571
H	-3.66994	0.43037	-2.58663
H	-2.98945	1.96866	-1.94941
H	-1.57912	-0.57610	-1.89097
H	-0.21754	-2.77112	0.37516
C	-0.13644	-2.06693	-0.48608
H	0.78843	-2.30830	-1.06002
H	-0.03181	-1.03196	-0.09850
O	-1.30176	-2.10385	-1.31079
H	-1.30702	-2.93836	-1.86613
C	-0.88941	1.52809	1.00210
H	-1.79805	2.12661	0.74080
H	-1.23970	0.53225	1.38351
H	-0.37804	2.05197	1.83755
O	0.01321	1.41429	-0.08098
H	-0.51215	1.08907	-0.87445

(4MeOH)@cycHC[8] – Hydrogen bond chain trough cavity

208

C	-0.49263	1.65733	10.04354
C	0.46049	0.46825	9.96827
C	0.28122	2.67910	7.88368
C	-0.91554	2.09640	8.63467
N	-2.08010	2.93257	8.94789
N	-1.79504	1.54804	10.71139
C	-2.65953	2.48343	10.13645
O	-3.73671	2.86481	10.61148
H	-1.26321	1.18721	8.07911
H	0.06555	2.50805	10.51838
C	-1.91587	1.19385	12.11555
N	-2.24064	-0.20190	12.36716
H	-2.71308	1.83736	12.54929
H	-0.95212	1.40376	12.62914
C	-1.38282	-0.99262	13.14149
N	-2.11302	-2.07927	13.62121
O	-0.19134	-0.75320	13.37643
C	-3.42429	-2.11439	12.96119
C	-4.64627	-2.65260	13.70303
C	-4.72657	-0.45693	11.59429
C	-3.62149	-0.62839	12.63341
H	-3.95933	-0.10964	13.57087
H	-3.33453	-2.67677	11.99761
C	-1.48779	-3.20884	14.28842
N	-1.92169	-8.24612	5.96619
C	-2.90937	-7.93248	5.03235
N	-2.43602	-6.87898	4.23642
O	-4.00202	-8.49688	4.90748
C	-0.99233	-6.74550	4.47000
C	-0.27798	-5.40808	4.29271
C	0.58316	-7.59858	6.22829
C	-0.86799	-7.23062	5.92103
H	-1.15620	-6.38314	6.59901
H	-0.46984	-7.49021	3.81320
C	-3.00341	-6.66779	2.91123
N	-3.59995	-5.35940	2.70440
C	-3.05527	-4.47099	1.77159
N	-4.02125	-3.50722	1.47703
O	-1.92205	-4.54127	1.28053
C	-5.14422	-3.65187	2.41098
C	-6.56485	-3.28938	1.98310
C	-5.86717	-5.47495	3.99050
C	-5.05290	-5.14778	2.74208
H	-5.50661	-5.71757	1.88873
H	-4.91296	-3.07286	3.34247
H	-3.77878	-7.44893	2.75713
H	-2.20065	-6.79132	2.15113
C	-3.69308	-2.28310	0.76250
N	-3.16380	-1.18338	1.55438
C	-3.99482	-0.26396	2.19893
N	-3.20409	0.49362	3.07308
O	-5.20968	-0.12803	2.01747
C	-1.79682	0.26154	2.73533
C	-0.70387	0.39805	3.79146
C	-0.58074	-1.47790	1.39297
C	-1.84496	-1.17399	2.19554
H	-1.86595	-1.87416	3.07169
H	-1.52660	0.94363	1.88615
C	-3.66399	1.79144	3.54768
N	-3.76472	1.91067	4.99202
H	-4.66466	1.96831	3.09703
H	-2.95785	2.57568	3.19539
C	-2.93744	2.79822	5.68910
N	-3.51659	3.04345	6.93300
O	-1.88624	3.29470	5.26279
C	-4.63690	2.11740	7.13735
C	-5.85086	2.53892	7.96284
C	-5.93439	0.59702	5.62137
C	-5.05766	1.84488	5.68650
C	-2.81090	3.74794	7.99173
H	-2.10458	4.43931	7.48832
H	-3.54043	4.33875	8.58190
C	0.63364	0.06080	3.08678
C	0.62131	-1.30169	2.35420

H	-0.50195	-0.78095	0.52756
H	-0.60596	-2.51760	1.00344
H	-0.91138	-0.30454	4.63211
H	-0.68297	1.42604	4.21359
H	1.47989	0.09184	3.81482
H	0.85847	0.87053	2.35538
H	1.57269	-1.43235	1.79352
H	0.59001	-2.11753	3.11468
C	1.43265	-6.32252	6.00802
H	0.91712	-8.41635	5.54934
H	0.68761	-7.95880	7.27488
C	1.21338	-5.65430	4.62997
H	-0.71598	-4.64118	4.97295
H	-0.38918	-5.03028	3.25267
H	1.77080	-4.69312	4.58859
H	1.65278	-6.30440	3.83785
H	2.51077	-6.56301	6.14004
H	1.17759	-5.59073	6.81087
C	1.36604	1.57463	7.81869
H	0.66818	3.57453	8.42206
H	-0.01153	2.99648	6.85936
C	1.70948	0.95664	9.19512
H	-0.03107	-0.37202	9.42881
H	0.73859	0.11210	10.98455
H	2.42519	0.11531	9.05795
H	2.24111	1.71673	9.81417
H	2.28751	1.98393	7.34885
H	1.00562	0.75950	7.14740
C	-5.86506	-2.45767	12.76711
C	-6.02134	-1.01144	12.23991
H	-4.48529	-1.03197	10.67202
H	-4.85105	0.61287	11.31792
H	-6.79483	-2.76814	13.29432
H	-5.74299	-3.13862	11.89491
H	-6.85298	-0.97571	11.50137
H	-6.32039	-0.34217	13.08160
H	-4.78478	-2.09905	14.66098
H	-4.51526	-3.73028	13.94025
C	-7.50061	-3.64491	3.16560
C	-7.33608	-5.09495	3.67944
H	-7.96338	-5.24227	4.58624
H	-7.72807	-5.80049	2.91003
H	-8.55891	-3.47209	2.86931
H	-7.29369	-2.93687	4.00258
H	-5.48103	-4.89683	4.86036
H	-5.78514	-6.55269	4.25114
H	-6.84593	-3.86110	1.06894
H	-6.63784	-2.20505	1.75149
C	-7.19531	0.89879	6.46800
C	-6.86813	1.37227	7.90455
H	-7.80706	1.66974	8.42254
H	-6.44963	0.51357	8.48053
H	-6.29225	3.47022	7.53892
H	-5.55950	2.74453	9.01562
H	-4.23933	1.16225	7.56562
H	-7.84315	-0.00475	6.51386
H	-7.79248	1.68621	5.95061
H	-5.37894	-0.27108	6.04030
H	-6.21331	0.35413	4.57249
H	-5.67803	2.71175	5.33399
H	-7.21499	-7.79806	8.46358
H	-6.33495	-7.86724	10.77811
H	-6.82569	-6.19544	10.39026
C	-6.07715	-6.98208	10.14821
C	-6.20477	-7.37333	8.65690
H	-6.12863	-6.45061	8.03568
H	-5.26213	-9.36679	8.67092
C	-5.12200	-8.37438	8.18291
C	-4.65822	-6.49133	10.53166
H	-4.58639	-6.30943	11.62600

H	-5.18472	-8.52374	7.08297
H	-4.03659	-8.54653	10.58110
C	-3.69580	-7.59295	10.09622
H	-4.42494	-5.52975	10.01922
C	-3.76462	-7.79269	8.57608
O	-2.97787	-5.60504	13.52858
H	-2.44369	-7.69406	12.38661
H	-3.66547	-6.78805	8.08803
N	-2.24454	-7.48384	10.31078
N	-2.53556	-8.55629	8.34072
C	-1.87210	-5.33888	13.04451
C	-1.67544	-7.37892	11.64749
C	-2.15659	-9.15851	7.07474
C	-1.60209	-8.24867	9.33180
N	-1.23099	-6.04610	12.02129
N	-1.02697	-4.29999	13.44281
H	-0.80887	-8.07298	11.69840
O	-0.41881	-8.61109	9.35406
C	0.18562	-5.65958	12.01952
C	0.09559	-4.20628	12.50455
H	-0.21279	-3.56688	11.63629
H	0.70803	-6.25460	12.81502
C	1.03396	-5.75604	10.75476
H	0.57939	-5.14233	9.94467
H	1.08044	-6.80147	10.38147
C	1.46306	-3.71804	12.97994
H	1.81428	-4.34247	13.83342
C	2.44577	-5.23432	11.11960
C	2.43407	-3.83433	11.77890
H	2.14088	-3.07508	11.01562
H	2.92089	-5.96127	11.81901
H	3.08687	-5.21414	10.21054
H	3.46386	-3.56465	12.10216
H	1.40215	-2.66291	13.32294
H	-2.21531	-3.64510	15.00269
H	-0.62153	-2.80971	14.85326
H	-2.94609	-2.55177	-0.01069
H	-4.61020	-1.90865	0.26564
H	-2.95829	-9.84851	6.74162
H	-1.23286	-9.74029	7.27070
C	-3.75286	-2.66777	6.02451
H	-3.36835	-2.14994	5.11235
H	-3.24785	-3.66197	6.10304
H	-4.83981	-2.84519	5.88009
O	-3.58973	-1.86909	7.18541
H	-2.59425	-1.74868	7.33572
H	0.05641	-2.98101	9.16180
C	-0.82862	-3.08500	8.49050
H	-0.22372	-1.85159	7.03887
O	-1.00187	-1.93340	7.67561
H	-0.71090	-4.01855	7.88700
H	-1.73221	-3.19825	9.12523
H	-6.58700	-2.03292	8.80388
C	-6.20496	-3.08411	8.87510
H	-4.32515	-2.64523	8.50770
O	-4.83677	-3.15245	9.21204
H	-6.78853	-3.59795	9.67054
H	-6.44613	-3.59400	7.90519
H	3.10196	-2.41840	5.61776
C	2.41935	-2.22273	6.47364
H	1.20026	-1.02749	5.45465
O	1.11585	-1.82943	6.01561
H	2.28946	-3.16482	7.04364
H	2.87736	-1.45706	7.14052

(4MeOH)@cycHC[8] – Hydrogen bond chain which is connected to cycHC[8]

208

C	0.43904	-3.06070	-4.86382
C	-0.77255	-1.15324	-5.45252
H	-0.78589	-0.55310	-4.50671
C	-1.73214	-2.33757	-5.27599
H	-1.89470	-2.80484	-6.28301
C	-3.08646	-1.85106	-4.76997
H	-2.95373	-1.33126	-3.79566
H	-3.78418	-2.69714	-4.59576
C	-3.64564	-0.88025	-5.83822
H	-3.89178	-1.46644	-6.75473
H	-4.60409	-0.44185	-5.48179
C	-2.66164	0.25379	-6.21027
H	-2.56311	0.94775	-5.34304
H	-3.08214	0.85468	-7.04701
C	-1.24853	-0.25238	-6.59084
H	-1.27880	-0.82740	-7.54522
H	-0.55381	0.60435	-6.72439
C	-5.06472	-2.66539	1.68380
C	-3.53897	-0.96698	5.09358
C	-1.71129	-2.37773	4.83355
H	-1.80339	-3.00843	5.75714
C	-1.21060	-0.99002	5.25760
H	-0.88572	-0.43880	4.33633
C	0.00705	-1.12614	6.16944
H	-0.26680	-1.69643	7.08686
H	0.37725	-0.12580	6.48058
C	1.09822	-1.87188	5.36088
H	1.45641	-1.19596	4.54927
H	1.97778	-2.06707	6.01318
C	0.61435	-3.20090	4.73269
H	0.44530	-3.94721	5.54338
H	1.41602	-3.62087	4.08521
C	-0.69635	-3.05832	3.91972
H	-0.53468	-2.44141	3.00521
H	-1.06158	-4.05501	3.58813
C	-2.55183	0.89105	6.43941
H	-3.58129	0.95466	6.84555
H	-1.82977	0.89606	7.28062
C	-0.99452	2.59631	5.49679
C	-3.14063	2.59047	4.57616
H	-3.04251	1.91354	3.68674
C	-2.43794	3.91548	4.24784
H	-2.73474	4.66227	5.03071
C	-2.91515	4.45639	2.90310
H	-2.69360	3.72091	2.09787
H	-2.38559	5.39593	2.63543
C	-4.44004	4.69189	3.02848
H	-4.85162	5.01799	2.04778
H	-4.61191	5.53607	3.73653
C	-5.21441	3.44854	3.52689
H	-6.28260	3.71331	3.68930
H	-5.20644	2.67104	2.72639
C	-4.63073	2.82603	4.81967
H	-4.76912	3.51214	5.68639
H	-5.14490	1.86813	5.05088
H	-2.30011	-4.80057	-4.68536
C	0.05177	4.52621	4.34401
H	-0.39702	5.54016	4.27605
C	0.90541	5.29172	2.15140
C	2.29255	3.84167	3.32317
H	2.85729	4.57985	3.95351
C	2.80573	3.95955	1.88176
H	2.43824	3.07524	1.30467
C	4.33356	3.92266	1.86697
H	4.74207	4.77716	2.45603
H	4.71550	4.00805	0.82615
C	4.75838	2.56618	2.48410
H	4.44156	1.75896	1.78594
H	5.86806	2.52113	2.56180
C	4.12566	2.29084	3.86911

H	4.56171	2.99091	4.62160
H	4.39764	1.26477	4.20337
C	2.58494	2.44864	3.87235
H	2.12352	1.68117	3.21231
H	2.17024	2.32372	4.89574
C	2.32117	5.84651	0.17115
H	1.72768	6.78198	0.21315
H	3.39793	6.10108	0.09327
C	2.91083	4.35500	-1.74720
C	0.62656	4.74668	-1.45871
H	0.30363	3.86093	-0.85326
C	0.89113	4.26508	-2.89183
H	0.99589	5.17106	-3.54773
C	-0.29322	3.45922	-3.41583
H	-0.47136	2.56951	-2.77196
H	-0.09874	3.08584	-4.44493
C	-1.51457	4.41119	-3.40560
H	-2.42816	3.85678	-3.71639
H	-1.35473	5.20277	-4.17488
C	-1.75757	5.08628	-2.03485
H	-2.58474	5.82526	-2.12335
H	-2.10537	4.31495	-1.30749
C	-0.50097	5.77666	-1.44872
H	-0.21456	6.65996	-2.06599
H	-0.70149	6.13135	-0.41442
C	2.92211	3.05260	-3.86211
H	2.56263	3.49876	-4.81557
H	4.00292	3.27983	-3.73309
C	2.12918	1.04251	-5.08601
C	3.70189	0.65026	-3.42279
H	4.64513	0.73083	-4.02678
C	3.02773	-0.67846	-3.78895
H	2.21790	-0.87270	-3.03793
C	4.03104	-1.82659	-3.70180
H	4.86475	-1.65798	-4.42178
H	3.54422	-2.79292	-3.95699
C	4.56088	-1.85924	-2.24625
H	3.72977	-2.18641	-1.57738
H	5.35527	-2.63305	-2.15405
C	5.09817	-0.49654	-1.74713
H	6.04554	-0.26172	-2.28826
H	5.36422	-0.57379	-0.66978
C	4.10116	0.67094	-1.95192
H	3.21723	0.55617	-1.28635
H	4.57640	1.63931	-1.68241
C	1.77139	-1.26288	-5.97989
H	2.46760	-2.10438	-6.17012
H	1.59779	-0.71586	-6.92895
N	0.51294	-1.85561	-5.55175
N	-0.90254	-3.25922	-4.47891
N	-3.06623	-2.06660	4.36382
N	-2.45426	-0.38768	5.75098
N	-2.28431	2.08425	5.65289
N	-1.02953	3.55746	4.47870
N	0.91402	4.34094	3.18419
N	2.09012	5.16037	1.43170
N	1.98352	5.12868	-1.04651
N	2.22054	3.66167	-2.74633
N	2.73858	1.61493	-3.96835
N	2.40859	-0.32660	-5.07153
O	1.37037	-3.84245	-4.64373
O	-4.71565	-0.59149	5.15470
O	-0.00222	2.27714	6.16108
O	0.01067	6.12139	1.94357
O	4.13023	4.31069	-1.54216
O	1.47723	1.64385	-5.94930
H	-6.07001	-3.02154	2.03300
H	0.67863	4.46209	5.25957
C	-3.98330	-3.11428	3.94458
C	-1.36017	-4.59333	-4.13114

C	-2.97847	-4.97037	-2.28033
C	-0.83502	-5.79347	-1.93972
H	-0.98937	-6.78193	-2.44323
C	-1.59208	-5.83124	-0.60609
H	-1.29123	-4.94175	0.00321
C	-1.21687	-7.08520	0.18268
H	-1.50684	-7.99461	-0.39208
H	-1.75037	-7.10371	1.15665
C	0.31418	-7.04512	0.41332
H	0.54406	-6.21226	1.11861
H	0.63807	-7.98023	0.92132
C	1.13140	-6.84504	-0.88359
H	1.04644	-7.76043	-1.51466
H	2.20969	-6.73533	-0.63356
C	0.66435	-5.62549	-1.71482
H	0.87049	-4.68074	-1.16471
H	1.21066	-5.56531	-2.67965
C	-4.15353	-5.73843	-0.22236
H	-5.02824	-5.82167	-0.89794
H	-4.07523	-6.66369	0.38230
C	-3.80120	-4.59486	1.96843
C	-4.79316	-3.28632	0.30698
H	-3.91372	-2.76299	-0.14888
C	-5.13275	-1.14423	1.58177
H	-4.16744	-0.74769	1.19094
H	-5.30366	-0.68378	2.57971
C	-6.30251	-0.81367	0.62158
H	-4.99279	-2.83384	4.31958
H	-6.34915	0.28414	0.44646
H	-7.25841	-1.08915	1.12555
C	-6.21598	-1.55142	-0.73644
H	-3.67577	-4.08061	4.40217
H	-7.13742	-1.35300	-1.32701
H	-5.37097	-1.12717	-1.32671
C	-5.99854	-3.07968	-0.60862
H	-6.89699	-3.57248	-0.17162
H	-5.81306	-3.52221	-1.61097
H	-0.58314	-5.32499	-4.43655
N	-1.62297	-4.78809	-2.69336
N	-2.97066	-5.64305	-1.07058
N	-4.37927	-4.64029	0.69853
N	-4.04439	-3.32486	2.50671
O	-3.96605	-4.58927	-2.90919
O	-3.19671	-5.51689	2.52749
C	-3.03150	0.87489	-1.81420
O	-1.70114	0.38413	-1.93847
H	-3.49972	0.59709	-0.83940
H	-3.68593	0.50135	-2.63618
H	-2.99778	1.98259	-1.88396
H	-1.69782	-0.62770	-1.86832
H	-0.27148	-2.90037	0.24257
C	-0.24230	-2.15704	-0.58889
H	0.66535	-2.34289	-1.20896
H	-0.15374	-1.14242	-0.15102
O	-1.43548	-2.18446	-1.36873
H	-1.43687	-3.00464	-1.94785
C	-0.99818	1.46025	1.12649
H	-1.87345	2.09198	0.84471
H	-1.38524	0.49055	1.53082
H	-0.44893	1.98607	1.93562
O	-0.11680	1.27758	0.02676
C	2.33546	-0.82986	1.37411
H	2.40058	-1.54103	0.50943
H	1.44810	-1.12617	1.99051
O	2.30693	0.52153	0.97794
H	3.24144	-0.99022	1.99904
H	1.43175	0.73839	0.53374
H	-0.65549	0.95349	-0.77191

Optimized geometry of non-interacting host and guest

197

P	12.00368	0.00661	-0.00261
C	0.54207	0.14992	-5.81553
C	-1.47789	1.09194	-5.12583
H	-1.31075	1.26306	-4.02986
C	-1.66412	-0.41490	-5.34719
H	-2.04946	-0.56323	-6.39025
C	-2.70446	-0.97671	-4.38250
H	-2.36211	-0.83413	-3.33325
H	-2.83473	-2.07075	-4.53301
C	-4.03134	-0.23000	-4.66371
H	-4.40853	-0.54122	-5.66622
H	-4.80284	-0.54735	-3.92754
C	-3.89174	1.31032	-4.63619
H	-3.70583	1.63950	-3.58660
H	-4.85293	1.77852	-4.94373
C	-2.74389	1.84631	-5.52728
H	-2.96439	1.67046	-6.60523
H	-2.61421	2.93938	-5.37466
C	-1.74171	-5.07610	0.69634
C	-0.20611	-4.15756	4.38623
C	1.83854	-4.00128	3.29122
H	2.42477	-4.80755	3.80626
C	1.70149	-2.81971	4.26227
H	1.34338	-1.93061	3.67885
C	3.06481	-2.46636	4.85429
H	3.47581	-3.34141	5.40806
H	2.97896	-1.61797	5.56728
C	3.98502	-2.08160	3.66860
H	3.62607	-1.11410	3.24491
H	5.01596	-1.89340	4.04154
C	4.02861	-3.14058	2.54112
H	4.57430	-4.04071	2.90905
H	4.61857	-2.74708	1.68387
C	2.62738	-3.58486	2.05221
H	2.10270	-2.75364	1.52757
H	2.71624	-4.42917	1.33365
C	0.09696	-2.56497	6.27374
H	-0.68460	-3.20891	6.72522
H	0.91680	-2.41717	7.00539
C	0.32931	-0.09462	6.08420
C	-1.60464	-0.98847	5.13179
H	-1.29484	-1.18722	4.07182
C	-1.76978	0.52819	5.30327
H	-2.31644	0.71057	6.26606
C	-2.62688	1.10129	4.17738
H	-2.14684	0.90035	3.19225
H	-2.73253	2.20376	4.27831
C	-4.01047	0.41140	4.27359
H	-4.65102	0.73297	3.42270
H	-4.51826	0.76859	5.19994
C	-3.93072	-1.13364	4.30570
H	-4.94465	-1.55501	4.48439
H	-3.61353	-1.50133	3.30143
C	-2.94285	-1.68913	5.36219
H	-3.30677	-1.48039	6.39434
H	-2.83722	-2.78993	5.25077
H	-0.77302	-2.78713	-6.02070
C	-0.03168	2.33365	5.77977
H	-0.90634	2.84571	6.23796
C	-0.34188	4.27448	4.27815
C	1.76644	3.29486	4.24881
H	2.27317	3.84137	5.08764
C	1.64815	4.25728	3.05891
H	1.38019	3.65606	2.15033
C	2.99556	4.92629	2.79147

H	3.31798	5.50112	3.68978
H	2.92287	5.63391	1.93756
C	4.00431	3.79555	2.46853
H	3.73165	3.35341	1.48128
H	5.02217	4.22688	2.34469
C	4.04437	2.67045	3.52993
H	4.51000	3.06683	4.46244
H	4.70548	1.84866	3.17605
C	2.64746	2.10264	3.88419
H	2.20758	1.55348	3.02033
H	2.72084	1.38764	4.73273
C	-0.04129	6.18213	2.70611
H	-0.88282	6.59126	3.30080
H	0.74941	6.95375	2.61284
C	0.35652	5.99954	0.25601
C	-1.59938	4.98639	1.02447
H	-1.26706	3.93603	1.23717
C	-1.67582	5.16129	-0.49905
H	-2.23514	6.11047	-0.71174
C	-2.46471	4.01586	-1.12857
H	-1.96873	3.04334	-0.90698
H	-2.51041	4.12355	-2.23464
C	-3.88898	4.06556	-0.52126
H	-4.48361	3.19943	-0.88730
H	-4.40174	4.98016	-0.90093
C	-3.90211	4.08583	1.02593
H	-4.94444	4.22922	1.38720
H	-3.57556	3.08885	1.40512
C	-2.98333	5.16882	1.64518
H	-3.36759	6.19042	1.42147
H	-2.93923	5.05191	2.74965
C	0.16886	5.67495	-2.18893
H	-0.66274	6.14765	-2.75698
H	1.01659	6.39145	-2.11139
C	-0.01706	4.20328	-4.16833
C	1.98330	4.06802	-2.99197
H	2.57933	4.87669	-3.49178
C	1.89232	2.87945	-3.95893
H	1.51387	1.99311	-3.38279
C	3.27847	2.52604	-4.49430
H	3.70685	3.39671	-5.04175
H	3.22162	1.66892	-5.19939
C	4.15513	2.15791	-3.27126
H	3.78488	1.19366	-2.85002
H	5.19998	1.97036	-3.60363
C	4.15151	3.23002	-2.15600
H	4.70365	4.12985	-2.51500
H	4.71296	2.85090	-1.27357
C	2.73067	3.66868	-1.72224
H	2.19469	2.83800	-1.20861
H	2.78796	4.52083	-1.00996
C	0.39415	2.62480	-6.05155
H	1.26627	2.45975	-6.71570
H	-0.33507	3.28341	-6.56464
N	-0.21358	1.32089	-5.83486
N	-0.26761	-0.87921	-5.31299
N	0.44733	-4.45189	3.18718
N	0.60615	-3.28763	5.11844
N	-0.45810	-1.24623	6.00930
N	-0.37849	0.95311	5.48849
N	0.35839	3.11280	4.61688
N	0.47913	5.04937	3.45556
N	-0.50031	5.89980	1.35477
N	-0.26559	5.38414	-0.83389
N	0.58375	4.50429	-2.94229
N	0.83141	3.33742	-4.86198
O	1.71288	0.02787	-6.19351
O	-1.30393	-4.60253	4.74234
O	1.44925	-0.01036	6.60237
O	-1.48161	4.57005	4.65715
O	1.46431	6.54941	0.24228
O	-1.10076	4.64188	-4.57133
H	-2.31401	-6.02011	0.90126
H	0.80781	2.31349	6.50945
C	0.07056	-5.61891	2.40846
C	0.08842	-2.26547	-5.55147
C	-0.30918	-4.17883	-4.03053
C	1.85502	-3.33416	-4.05479
H	2.24781	-3.92704	-4.92107
C	1.70973	-4.28646	-2.86125
H	1.51369	-3.67328	-1.94422
C	3.00603	-5.06592	-2.64582
H	3.23393	-5.67685	-3.54922
H	2.90843	-5.75387	-1.77855
C	4.12671	-4.02904	-2.38687
H	3.93933	-3.54630	-1.39873
H	5.10461	-4.55145	-2.29737
C	4.22078	-2.93194	-3.47274
H	4.59021	-3.38725	-4.42124
H	4.97805	-2.17408	-3.17366
C	2.86823	-2.23354	-3.75618
H	2.54379	-1.63903	-2.87178
H	2.95568	-1.53151	-4.61350
C	-0.08684	-6.11592	-2.48520
H	-0.93266	-6.49871	-3.09108
H	0.68443	-6.90681	-2.39423
C	0.29368	-5.93065	-0.03522
C	-1.64318	-4.89180	-0.82407
H	-1.29257	-3.84853	-1.03192
C	-2.52357	-3.92299	1.32073
H	-2.01253	-2.95739	1.10579
H	-2.58525	-4.03508	2.42559
C	-3.94017	-3.95301	0.69497
H	-0.76502	-6.11450	2.95138
H	-4.52942	-3.08152	1.05774
H	-4.46910	-4.86320	1.06368
C	-3.93172	-3.96461	-0.85204
H	0.93261	-6.31893	2.34031
H	-4.97147	-4.08874	-1.22854
H	-3.57747	-2.97409	-1.22032
C	-3.02111	-5.06016	-1.46153
H	-3.42346	-6.07718	-1.24618
H	-2.96368	-4.93812	-2.56475
H	0.95467	-2.28170	-6.24775
N	0.44183	-3.01305	-4.34026
N	0.47411	-4.99069	-3.22372
N	-0.55145	-5.82559	-1.14164
N	-0.33849	-5.31656	1.04901
O	-1.45303	-4.41256	-4.42673
O	1.39984	-6.48507	-0.01063
H	1.26794	-1.14659	-0.91431
C	0.27163	-0.72387	-1.19853
H	0.44941	0.18049	-1.83358
H	-0.23084	-0.39158	-0.26462
O	-0.57475	-1.67156	-1.82446
H	-0.19414	-1.92928	-2.70691
F	11.04959	0.88442	-1.02381
F	10.68381	-0.85009	0.49404
F	11.73766	1.11074	1.19473
F	12.95790	-0.87080	1.01850
F	13.32387	0.86384	-0.49942
F	12.26989	-1.09709	-1.19995
C	0.45441	-13.82451	2.69309
H	1.52441	-13.82451	2.69309
H	0.09774	-13.28391	3.54482
H	0.09774	-14.83243	2.73540
H	0.09774	-13.35720	1.79905

PF_6^- ion on the mouth of cycHC[8]

197

P	3.94667	0.12121	0.05340
C	0.53020	0.18053	-5.89145
C	-1.54857	1.04945	-5.28420
H	-1.43149	1.22080	-4.18293
C	-1.67206	-0.46166	-5.51869
H	-1.99889	-0.61747	-6.58134
C	-2.73918	-1.06621	-4.61102
H	-2.45109	-0.92418	-3.54572
H	-2.82435	-2.16256	-4.77650
C	-4.07527	-0.36145	-4.94931
H	-4.39403	-0.67375	-5.97165
H	-4.87042	-0.71174	-4.25400
C	-3.98819	1.18199	-4.90032
H	-3.85758	1.50441	-3.84042
H	-4.94999	1.62256	-5.24501
C	-2.82057	1.76387	-5.73516
H	-2.98846	1.59228	-6.82356
H	-2.73289	2.85800	-5.56396
C	-1.74110	-4.97096	0.70041
C	-0.31221	-4.18407	4.44406
C	1.80388	-4.16589	3.47870
H	2.29324	-5.01739	4.02304
C	1.69011	-2.98442	4.45201
H	1.43872	-2.06790	3.85782
C	3.03291	-2.74220	5.13781
H	3.33242	-3.64860	5.71364
H	2.96319	-1.88956	5.84782
C	4.05726	-2.42402	4.02023
H	3.80793	-1.43470	3.57689
H	5.07276	-2.32347	4.46503
C	4.09501	-3.46738	2.87784
H	4.56229	-4.40957	3.25150
H	4.74423	-3.08244	2.06170
C	2.69719	-3.79827	2.29852
H	2.28975	-2.91966	1.75346
H	2.76708	-4.63886	1.57299
C	-0.01721	-2.61457	6.35445
H	-0.85066	-3.21141	6.77649
H	0.77488	-2.49920	7.12159
C	0.34154	-0.15771	6.12714
C	-1.61300	-0.97884	5.14981
H	-1.29509	-1.20968	4.09969
C	-1.71140	0.54561	5.29564
H	-2.26453	0.76781	6.24696
C	-2.52582	1.13860	4.14843
H	-2.04043	0.90055	3.17394
H	-2.58195	2.24567	4.23241
C	-3.94032	0.51389	4.23427
H	-4.55362	0.85064	3.36900
H	-4.44491	0.90895	5.14721
C	-3.93193	-1.03207	4.29150
H	-4.96658	-1.40437	4.46183
H	-3.61674	-1.42935	3.29799
C	-2.98470	-1.61396	5.37044
H	-3.35371	-1.37263	6.39380
H	-2.92617	-2.71950	5.27592
H	-0.65327	-2.81591	-6.10551
C	0.09015	2.28076	5.78353
H	-0.75747	2.81570	6.26677
C	-0.24204	4.19840	4.25929
C	1.88325	3.25769	4.24944
H	2.36744	3.83877	5.07974
C	1.74882	4.18980	3.03699
H	1.50334	3.56392	2.14101
C	3.07950	4.88999	2.76784
H	3.36725	5.50506	3.65209
H	2.99684	5.56837	1.89064
C	4.12722	3.78215	2.49334
H	3.89108	3.29439	1.52234

H	5.13347	4.24413	2.37729
C	4.18548	2.68379	3.58189
H	4.63135	3.10572	4.51409
H	4.85641	1.87020	3.23416
C	2.79820	2.08317	3.91889
H	2.40876	1.51368	3.04766
H	2.87474	1.38018	4.77792
C	0.02330	6.08340	2.65526
H	-0.81615	6.49205	3.25349
H	0.80538	6.86145	2.54192
C	0.38117	5.92303	0.19453
C	-1.51575	4.82832	1.00130
H	-1.14070	3.79156	1.20714
C	-1.62980	5.00157	-0.51947
H	-2.23678	5.92466	-0.71857
C	-2.38087	3.82257	-1.13380
H	-1.83878	2.87298	-0.91938
H	-2.44795	3.92484	-2.23890
C	-3.79512	3.81561	-0.50178
H	-4.36094	2.92444	-0.85391
H	-4.35164	4.70696	-0.87572
C	-3.78328	3.84266	1.04538
H	-4.82429	3.94869	1.42376
H	-3.41266	2.86069	1.42258
C	-2.89487	4.96112	1.64548
H	-3.32122	5.96692	1.42452
H	-2.82758	4.85195	2.74936
C	0.13058	5.62794	-2.24987
H	-0.74891	6.06476	-2.77297
H	0.93950	6.38930	-2.18763
C	-0.09673	4.14496	-4.21257
C	2.01433	4.22485	-3.24492
H	2.45415	5.09995	-3.79499
C	1.96053	3.03708	-4.21553
H	1.76507	2.10775	-3.62214
C	3.31006	2.87540	-4.91259
H	3.54121	3.79162	-5.50425
H	3.28825	2.00966	-5.60959
C	4.37060	2.63988	-3.80731
H	4.19409	1.63966	-3.35512
H	5.38392	2.61201	-4.26791
C	4.34799	3.68866	-2.66891
H	4.73542	4.66271	-3.05200
H	5.03464	3.35750	-1.86096
C	2.93705	3.91457	-2.07091
H	2.59730	3.00621	-1.52744
H	2.95266	4.75590	-1.34289
C	0.29342	2.64823	-6.16479
H	1.14432	2.52291	-6.86408
H	-0.48107	3.27900	-6.64509
N	-0.26572	1.32341	-5.94307
N	-0.26324	-0.87409	-5.41506
N	0.39105	-4.51499	3.28478
N	0.51058	-3.37630	5.23344
N	-0.49618	-1.27383	6.05309
N	-0.30580	0.91087	5.49827
N	0.47509	3.05360	4.61595
N	0.56075	4.96661	3.41498
N	-0.44615	5.78131	1.31173
N	-0.24037	5.29068	-0.88586
N	0.58569	4.50259	-3.04626
N	0.75954	3.37100	-4.99372
O	1.71660	0.09999	-6.22849
O	-1.45777	-4.55686	4.73007
O	1.45040	-0.11408	6.67306
O	-1.38307	4.48838	4.64356
O	1.46217	6.52338	0.16072
O	-1.25294	4.47688	-4.50654
H	-2.36532	-5.88875	0.87190
H	0.94656	2.22918	6.49131
C	-0.02292	-5.63807	2.46274
C	0.15767	-2.25042	-5.59858
C	-0.25045	-4.13900	-4.05023
C	1.87541	-3.20407	-3.97627
H	2.34401	-3.80014	-4.80362
C	1.70360	-4.13265	-2.76795
H	1.43596	-3.50600	-1.87981
C	3.01870	-4.85018	-2.46789
H	3.31751	-5.47016	-3.34500
H	2.90619	-5.52675	-1.59279
C	4.07625	-3.75703	-2.17368
H	3.81524	-3.25240	-1.21717
H	5.07061	-4.23425	-2.02265
C	4.18093	-2.67428	-3.27335
H	4.63739	-3.11711	-4.19071
H	4.86147	-1.87042	-2.92113
C	2.81349	-2.05085	-3.64390
H	2.42625	-1.45831	-2.78768
H	2.91577	-1.35722	-4.50678
C	-0.03295	-6.03014	-2.44636
H	-0.86061	-6.42739	-3.06856
H	0.74761	-6.80984	-2.33362
C	0.27689	-5.90733	0.02065
C	-1.58449	-4.76967	-0.81267
H	-1.18899	-3.73781	-0.99663
C	-2.48723	-3.78935	1.31570
H	-1.92013	-2.84856	1.13470
H	-2.59316	-3.91403	2.41566
C	-3.88046	-3.73833	0.63993
H	-0.90640	-6.09608	2.96104
H	-4.43678	-2.84180	0.99431
H	-4.46884	-4.62447	0.97656
C	-3.81764	-3.73054	-0.90592
H	0.79991	-6.38522	2.40932
H	-4.84866	-3.79050	-1.32095
H	-3.38808	-2.76122	-1.24738
C	-2.94743	-4.86821	-1.49586
H	-3.40705	-5.86560	-1.30150
H	-2.84860	-4.74392	-2.59583
H	1.06416	-2.24434	-6.24191
N	0.46884	-2.95742	-4.35383
N	0.51895	-4.89809	-3.17845
N	-0.52449	-5.74302	-1.11101
N	-0.36421	-5.28564	1.09582
O	-1.36131	-4.43382	-4.50157
O	1.35346	-6.51586	0.07089
H	0.90369	-0.65565	-0.98226
C	-0.14015	-0.42629	-1.29306
H	-0.12088	0.47542	-1.95534
H	-0.71711	-0.16328	-0.38019
O	-0.80107	-1.53139	-1.90123
H	-0.32288	-1.78259	-2.73356
F	4.90122	1.47150	-0.00558
F	5.23856	-0.80471	-0.40243
F	4.36151	-0.09795	1.64068
F	2.65385	1.04397	0.50828
F	2.99668	-1.23559	0.11138
F	3.53851	0.33762	-1.53517

PF_6^-@cycHC[8]

197

P	-0.19805	-0.01479	0.19625
C	0.47801	0.22379	-5.94933
C	-1.47170	1.13261	-5.04207
H	-1.18808	1.34288	-3.97931
C	-1.63614	-0.38624	-5.19361
H	-2.17244	-0.58638	-6.15987
C	-2.50080	-0.93595	-4.06279
H	-2.03358	-0.70763	-3.07918
H	-2.60416	-2.04066	-4.13944
C	-3.88624	-0.25449	-4.18929
H	-4.39504	-0.64390	-5.10343
H	-4.50787	-0.53389	-3.31214
C	-3.80913	1.28923	-4.26497
H	-3.51309	1.67291	-3.26190
H	-4.82210	1.70257	-4.47176
C	-2.80398	1.82777	-5.31373
H	-3.14609	1.60154	-6.35032
H	-2.70107	2.93052	-5.21861
C	-1.78354	-5.02468	0.74596
C	-0.26948	-4.20236	4.50030
C	1.74092	-4.01292	3.34482
H	2.36271	-4.80825	3.83675
C	1.61106	-2.83320	4.31917
H	1.21799	-1.95622	3.74495
C	2.98590	-2.45951	4.87009
H	3.43196	-3.33016	5.40444
H	2.90905	-1.61699	5.59174
C	3.85702	-2.05206	3.65606
H	3.45826	-1.09522	3.24746
H	4.89650	-1.84227	3.99353
C	3.88179	-3.10613	2.52398
H	4.45845	-3.99703	2.86952
H	4.43426	-2.69682	1.64901
C	2.47289	-3.57182	2.07934
H	1.91131	-2.75004	1.58247
H	2.55799	-4.41252	1.35596
C	0.05160	-2.58573	6.36206
H	-0.74801	-3.21298	6.80598
H	0.86723	-2.45282	7.10199
C	0.34477	-0.12268	6.19333
C	-1.56035	-0.97137	5.14886
H	-1.19598	-1.16513	4.10796
C	-1.70427	0.54752	5.31978
H	-2.29316	0.74104	6.25630
C	-2.48730	1.13919	4.15025
H	-1.95892	0.93312	3.19326
H	-2.58207	2.24231	4.25294
C	-3.88675	0.47615	4.17635
H	-4.47637	0.81348	3.29523
H	-4.43606	0.83558	5.07870
C	-3.83611	-1.06977	4.19795
H	-4.86597	-1.47654	4.31148
H	-3.45897	-1.42861	3.21227
C	-2.92098	-1.64662	5.30676
H	-3.33906	-1.43211	6.31787
H	-2.83138	-2.74950	5.20102
H	-0.71350	-2.73548	-6.07803
C	0.07395	2.31194	5.82682
H	-0.76296	2.86215	6.31183
C	-0.21577	4.25037	4.31608
C	1.79038	3.09063	4.10509
H	2.43543	3.60502	4.86710
C	1.62609	4.03019	2.90126
H	1.20823	3.42977	2.05383
C	2.99017	4.56672	2.47157
H	3.46555	5.11994	3.31445
H	2.89278	5.27055	1.61601
C	3.84225	3.33750	2.06901
H	3.40650	2.89967	1.14166
H	4.87432	3.66382	1.81061
C	3.90176	2.23880	3.15664
H	4.50763	2.61060	4.01697
H	4.43984	1.35088	2.75620
C	2.50799	1.81131	3.68056
H	1.92250	1.28842	2.89235
H	2.61864	1.11439	4.54055
C	0.07051	6.08542	2.66302
H	-0.73878	6.50994	3.29189

H	0.87509	6.83986	2.54122
C	0.37261	6.00803	0.19888
C	-1.46464	4.82240	1.00849
H	-1.03496	3.80157	1.16611
C	-1.62278	5.03446	-0.50408
H	-2.27424	5.93441	-0.66836
C	-2.31646	3.83100	-1.13606
H	-1.70705	2.91491	-0.98184
H	-2.43688	3.97259	-2.23281
C	-3.70470	3.69450	-0.46245
H	-4.18124	2.75142	-0.81056
H	-4.35429	4.53709	-0.79966
C	-3.64647	3.68794	1.08441
H	-4.68044	3.69708	1.49649
H	-3.17908	2.73250	1.41678
C	-2.83220	4.86030	1.68654
H	-3.33340	5.83685	1.49025
H	-2.73621	4.74313	2.78803
C	0.12402	5.70755	-2.24643
H	-0.72852	6.18133	-2.78252
H	0.97009	6.42947	-2.18701
C	-0.08501	4.28294	-4.25903
C	1.88643	4.04325	-3.04792
H	2.53382	4.83427	-3.51300
C	1.76486	2.87858	-4.04133
H	1.33880	2.00045	-3.49169
C	3.14851	2.48775	-4.55745
H	3.62238	3.35628	-5.07052
H	3.07678	1.65334	-5.28880
C	3.98070	2.05486	-3.32522
H	3.55602	1.10116	-2.93587
H	5.02489	1.83047	-3.63774
C	3.99286	3.09751	-2.18285
H	4.58953	3.98399	-2.50482
H	4.51749	2.67234	-1.29854
C	2.57887	3.57615	-1.76921
H	1.99852	2.75324	-1.29535
H	2.65642	4.40667	-1.03335
C	0.26850	2.69234	-6.13821
H	1.11266	2.54109	-6.84145
H	-0.48896	3.35039	-6.61028
N	-0.30881	1.37819	-5.90468
N	-0.24373	-0.81208	-5.36026
N	0.35854	-4.49518	3.29156
N	0.55174	-3.32204	5.21163
N	-0.46807	-1.25500	6.08934
N	-0.31545	0.93808	5.57582
N	0.42297	3.05151	4.62937
N	0.57927	4.93515	3.39334
N	-0.43265	5.81953	1.32510
N	-0.25335	5.39363	-0.88358
N	0.51094	4.54670	-3.02584
N	0.74201	3.39852	-4.95753
O	1.61019	0.13247	-6.44287
O	-1.35461	-4.65832	4.88532
O	1.44852	-0.06564	6.75203
O	-1.28972	4.64644	4.78865
O	1.44640	6.62471	0.16413
O	-1.14915	4.76388	-4.66955
H	-2.38720	-5.96393	0.86775
H	0.94483	2.27791	6.51972
C	-0.03207	-5.64893	2.50391
C	0.12249	-2.19800	-5.57764
C	-0.24801	-4.11242	-4.05643
C	1.78129	-2.99956	-3.81641
H	2.42483	-3.52749	-4.57067
C	1.58024	-3.93660	-2.61668
H	1.16653	-3.33000	-1.77231
C	2.92689	-4.50672	-2.17447
H	3.39820	-5.06812	-3.01441
H	2.80248	-5.21000	-1.32241
C	3.80383	-3.29949	-1.75900
H	3.36814	-2.85417	-0.83509
H	4.82484	-3.65123	-1.49001
C	3.90181	-2.20036	-2.84332
H	4.50703	-2.58552	-3.69821
H	4.45791	-1.32660	-2.43618
C	2.52400	-1.73846	-3.38017
H	1.94439	-1.20680	-2.59326
H	2.65862	-1.04095	-4.23611
C	-0.01758	-5.96350	-2.41144
H	-0.82472	-6.37072	-3.05440
H	0.77540	-6.72983	-2.28804
C	0.25557	-5.90005	0.05678
C	-1.61089	-4.75856	-0.75649
H	-1.22051	-3.71709	-0.87769
C	-2.56437	-3.88915	1.40148
H	-2.03209	-2.92310	1.25786
H	-2.67106	-4.05875	2.49542
C	-3.95479	-3.86979	0.72024
H	-0.88833	-6.12364	3.03239
H	-4.55445	-3.02079	1.11678
H	-4.50379	-4.79947	1.00276
C	-3.87919	-3.77108	-0.82176
H	0.81265	-6.37201	2.44469
H	-4.90299	-3.85169	-1.25113
H	-3.50526	-2.75787	-1.09548
C	-2.96227	-4.84299	-1.46405
H	-3.39751	-5.86168	-1.33549
H	-2.85179	-4.65814	-2.55507
H	1.01344	-2.19581	-6.24522
N	0.42299	-2.92786	-4.36087
N	0.51849	-4.81523	-3.12440
N	-0.53486	-5.70785	-1.07814
N	-0.40517	-5.32661	1.14131
O	-1.32340	-4.48425	-4.54521
O	1.34301	-6.49219	0.09857
C	-5.23878	0.08704	0.22079
O	-4.37425	0.19710	-0.89573
H	-3.44010	0.15336	-0.57250
F	1.43598	0.12833	0.05595
F	-0.40487	0.68541	-1.28701
F	-0.14723	-1.50626	-0.51458
F	-0.02740	-0.71623	1.68344
F	-1.86386	-0.15988	0.34624
F	-0.28345	1.47442	0.91336
H	-6.28393	0.08932	-0.15906
H	-5.13518	0.94174	0.93558
H	-5.08671	-0.86055	0.79448

BF_4^-

5

F	15.3576157	14.2614860	13.9146461
F	14.6157955	12.9819289	12.1332159
F	15.3726883	11.9500903	14.0632785
F	13.3619798	13.0991059	14.0763384
B	14.6771204	13.0729965	13.5468762

$CF_3SO_3^-$

8

C	14.6706459	13.4688462	13.3815462
F	13.3830799	13.7839782	13.6867865
F	14.7357092	13.3617159	12.0268732
F	15.4272980	14.5458921	13.7243453
S	15.2476512	11.8764110	14.2577189
O	14.2856065	10.8641378	13.7241549
O	16.6576786	11.7502153	13.7770779
O	15.0815209	12.2338544	15.6996159

ClO_4^-

5

Cl	5.2697885	12.8122964	6.8187481
O	4.8775544	14.2644552	6.9820136
O	5.1117013	12.4067739	5.3695871
O	6.7118960	12.6294384	7.2386444
O	4.3791669	11.9487393	7.6839770

IO_4^-

5

I	10.3387771	7.5720079	18.2822249
O	10.7969274	9.3607987	18.2294511
O	11.8307620	6.5681933	18.7027377
O	9.0404173	7.3091055	19.5687262
O	9.6871047	7.0459371	16.6344466

PF_6^-

7

F	15.0023558	8.3647377	5.6241584
F	14.8961885	7.8296155	8.8857303
F	14.2620924	9.5835465	7.4763115
F	16.4510422	8.7693781	7.4150824
F	15.6380200	6.6118160	7.0334953
F	13.4482973	7.4259102	7.0953323
P	14.9497295	8.0974899	7.2549939

ReO_4^-

5

Re	5.1109886	12.7265964	6.7658281
O	5.0651588	12.2787051	5.0535167
O	4.3911909	14.3299022	6.9783967
O	4.1988639	11.5386610	7.7112526
O	6.7932761	12.7552583	7.3171094

SbF_6^-

7

Sb	0.2670499	7.0222380	8.1115777
F	2.1359266	6.7862580	7.6015756
F	0.0900154	5.0950598	8.3580431
F	-1.6020772	7.2576485	8.6209681
F	0.7991755	7.2148063	9.9792570
F	-0.2642308	6.8300912	6.2435348
F	0.4435269	8.9495875	7.8659132

5. References

1. E. Prigorchenko, M. Öeren, S. Kaabel, M. Fomitšenko, I. Reile, I. Järving, T. Tamm, F. Topić, K. Rissanen, R. Aav, *Chem. Commun.* 2015, **51**, 10921.
2. *CrysAlisPro*; Agilent Technologies Ltd: Yarnton, Oxfordshire, England, 2014.
3. R. C. Clark, J. S. Reid, *Acta Crystallogr.* 1995, **A51**, 887.
4. G. M. Sheldrick, *Acta Crystallogr.* 2008, **A64**, 112.
5. L. J. Farrugia, *J. Appl. Crystallogr.* 2012, **45**, 849.
6. O. V. Dolomanov, L. J. Bourhis, R. J. Gildea, J. A. K. Howard, H.-J. Puschmann, *J. Appl. Crystallogr.* 2009, **42**, 339.
7. C. F. Macrae, P. R. Edgington, P. McCabe, E. Pidcock, G. P. Shields, R. Taylor, M. Towler, J. van de Streek, *J. Appl. Crystallogr.* 2006, **39**, 453.
8. *Persistence of Vision (TM) Raytracer*; Persistence of Vision Pty. Ltd.: Williamstown, Victoria, Australia, 2004.
9. *CrystalExplorer* (Version 3.1), S. K. Wolff, D. J. Grimwood, J. J. McKinnon, M. J. Turner, D. Jayatilaka, M. A. Spackman, University of Western Australia: Crawley, Australia, 2012.
10. A. L. Spek, *Acta Crystallogr.* 2009, **D65**, 148.
11. M. A. Spackman, D. Jayatilaka, *CrystEngComm*, 2009, **11**, 19.
12. Y. R. Hristova, M. M. J. Smulders, J. K. Clegg, B. Breinera, J. R. Nitschke, *Chem. Sci.*, 2011, **2**, 638.
13. M. Fomitšenko, E. Shmatova, M. Öeren, I. Järving, R. Aav, *Supramol. Chem.*, 2014, **26**, 698.
14. P. A. M. Dirac, *Proc. R. Soc. A*, 1929, **123**, 714; J. C. Slater, *Phys. Rev.*, 1951, **81**, 385; S. H. Vosko, L. Wilk, M. Nusair, *Can. J. Phys.*, 1980, **58**, 1200; A. D. Becke, *Phys. Rev. A: At., Mol., Opt. Phys.*, 1988, **38**, 3098; J. P. Perdew, *Phys. Rev. B: Condens. Matter Mater. Phys.*, 1986, **33**, 8822.
15. F. Weigend, R. Ahlrichs, *Phys. Chem. Chem. Phys.* 2005, **7**, 3297.
16. D. Andrae, U. Haeussermann, M. Dolg, H. Stoll, H. Preuss, *Theor. Chim. Acta*, 1990, **77**, 123; B. Metz, H. Stoll, M. Dolg, *J. Chem. Phys.*, 2000, **113**, 2563; K. A. Peterson, D. Figgen, E. Goll, H. Stoll, M. Dolg, *J. Chem. Phys.*, 2003, **119**, 11113; T. Leininger, A. Nicklass, W. Kuechle, H. Stoll, M. Dolg, A. Bergner, *Chem. Phys. Lett.*, 1996, **255**, 274; M. Kaupp, P. V. Schleyer, H. Stoll, H. Preuss, *J. Chem. Phys.*, 1991, **94**, 1360.
17. A. Klamt, G. Schüürmann, *J. Chem. Soc., Perkin Trans. 2*, 1993, **5**, 799.
18. O. Treutler, R. Ahlrichs, *J. Chem. Phys.*, 1995, **102**, 346; M. Von Arnim, R. Ahlrichs, *J. Comput. Chem.*, 1998, **19**, 1746; R. Ahlrichs, M. Bär, M. Häser, H. Horn, C. Kölmel, *Chem. Phys. Lett.*, 1989, **162**, 165; K. Eichkorn, F. Weigend, O. Treutler, R. Ahlrichs, *Theor. Chem. Acc.*, 1997, **97**, 119.
19. Gaussian 09, Revision C.01, M. J. Frisch, G. W. Trucks, H. B. Schlegel, G. E. Scuseria, M. A. Robb, J. R. Cheeseman, G. Scalmani, V. Barone, B. Mennucci, G. A. Petersson, H. Nakatsuji, M. Caricato, X. Li, H. P. Hratchian, A. F. Izmaylov, J. Bloino, G. Zheng, J. L. Sonnenberg, M. Hada, M. Ehara, K. Toyota, R. Fukuda, J. Hasegawa, M. Ishida, T. Nakajima, Y. Honda, O. Kitao, H. Nakai, T. Vreven, J. A. Montgomery, Jr., J. E. Peralta, F. Ogliaro, M. Bearpark, J. J. Heyd, E. Brothers, K. N. Kudin, V. N. Staroverov, T. Keith, R. Kobayashi, J. Normand, K. Raghavachari, A. Rendell, J. C. Burant, S. S. Iyengar, J. Tomasi, M. Cossi, N. Rega, J. M. Millam, M. Klene, J. E. Knox, J. B. Cross, V. Bakken, C. Adamo, J. Jaramillo, R. Gomperts, R. E. Stratmann, O. Yazyev, A. J. Austin, R. Cammi, C. Pomelli, J. W. Ochterski, R. L. Martin, K. Morokuma, V. G. Zakrzewski, G. A. Voth, P. Salvador, J. J. Dannenberg, S. Dapprich, A. D. Daniels, O. Farkas, J. B. Foresman, J. V. Ortiz, J. Cioslowski, and D. J. Fox, Gaussian, Inc., Wallingford CT, 2010.
20. http://periodic.lanl.gov/list.shtml (entered 6th February 2016)
21. F. Biedermann, V. D. Uzunova, O. A. Scherman, W. M. Nau, A. De Simone, *J. Am. Chem. Soc*, 2012, **134**, 15318.
22. M. Öeren, E. Shmatova, T. Tamm, R. Aav, *Phys. Chem. Chem. Phys.*, 2014, **16**, 19198.
23. D. R. Allan, S. J. Clark, M. J. P. Brugmans, G. J. Ackland, W. L. Vos, *Physical Review B*, 1998, **58**, R11809(R)
